# Time-to-death approach to reveal chronic and cumulative toxicity of a fungicide for honeybees not revealed with the standard ten-day test

**DOI:** 10.1038/s41598-018-24746-9

**Published:** 2018-05-08

**Authors:** Noa Simon-Delso, Gilles San Martin, Etienne Bruneau, Louis Hautier

**Affiliations:** 1 Beekeeping Research and Information Centre (CARI), Place Croix du Sud 4, 1348 Louvain la Neuve, Belgium; 2 Walloon Agricultural Research Centre, Life Sciences Department, Plant Protection and Ecotoxicology Unit, Rue de Liroux, 2, B-5030 Gembloux, Belgium

## Abstract

Synthetic fungicides are pesticides widely used in agriculture to control phytopathogenic fungi. The systemicity, persistency and intense application of some of these fungicides, such as boscalid, leads to long periods of exposure for honeybees via contaminated water, pollen and nectar. We exposed adult honeybees in the lab to food contaminated with boscalid for 33 days instead of the standard 10-day test. Most of the toxic effects were observed after 10 days. The median time to death (LT_50_) ranged from 24.9 days (lowest concentration) to 7.1 days (highest concentration) and was significantly shorter in all cases than with the control (32.0 days). The concentration and dietary doses of boscalid inducing 50% mortality (LC_50_ and LDD_50_, respectively) decreased strongly with the time of exposure: LC_50_ = 14,729 and 1,174 mg/l and LDD_50_ = 0.318 and 0.0301 mg bee^−1^ day^−1^ at days 8 and 25, respectively. We found evidence of reinforced toxicity when exposure is prolonged, but with an unusual pattern: no cumulative toxicity is observed until 17–18 days, when a point of inflexion appears that suggests a reduced capacity of bees to deal with the toxicant. Our results show the importance of time-to-death experiments rather than fixed-duration studies for evaluating chronic toxicity.

## Introduction

Fungicides and bactericides were the most widely used plant protection products in Europe in 2015 (41.76%), followed by herbicides (33.92%)^1^. Thus, it is not surprising to find fungicide residues in the environment and our food, for example in water^2–4^, air^5^, fruits^6,7^, vegetables^6,7^ or processed food^8^. Fungicide contamination has also been found in flower matrices such as nectar^9,10^, or pollen^10,11^. As a result, bees can be exposed to fungicides in flight, when collecting water, nectar or pollen or when consuming them afterwards.

One of the fungicides frequently detected in beekeeping matrices is boscalid, which is found in a range from a few μg/kg (ppb) up to 26.2 mg/kg (ppm), with pollen containing the largest amounts^10,12–24^. This molecule is authorised for a wide variety of uses. It is systemic (Kow = 2.96) and persistent in the soil (DT_50_ = 246 days)^25^. Simon-Delso *et al*.^11,12^ found that boscalid was the most frequent fungicide within beehives and in pollen pellets and contaminated samples of these pollen were found from July to October, ranging from 0.9 to 512 μg/kg. David *et al*.^23^ described the presence of boscalid residues both in oilseed rape pollen (up to 25 μg/kg) and pollen from field margins (up to 38 μg/kg), and proved that bees readily collect this pollen and bring the residues to the hive (up to 21 μg/kg). These results show that honeybees are frequently exposed to boscalid, including over extended periods.

A handful of studies have examined the eco-toxicity of this molecule. Boscalid inhibits mitochondrial respiration (succinate dehydrogenase) and blocks ATP production which affects cell respiration^26^. In mammals, it increases liver enzymes (alanine aminotransferase and gamma-glutamyl transferase), and is proved to induce thyroid adenomas although these reactions are considered adaptive and reversible^3^. Boscalid shows a low acute toxicity for honeybees, with an oral LD_50_ > 11 μg/bee^14^ and 100 μg/bee^27^ and a contact LD_50_ > 100 μg/bee^27^. To our knowledge, the biochemical effects on insects have not yet been described, but recently, boscalid (in co-formulation with pyraclostrobin) was found to be linked to decreased pollen consumption and digestion in bees, as well as lower ATP concentrations in the thoracic muscle tissue and higher virus titres^28^. A recent field study also revealed a significant positive correlation between honeybee colony disorders over the winter period and the presence of fungicides residues (mainly boscalid) in bee hives in the summer and autumn^12^. In addition, boscalid has been shown to interact with neonicotinoid insecticides such as thiamethoxam and clothianidin, whose toxicity to honeybees nearly double^24^. Disruptive effects on nest recognition of solitary bees *Osmia lignaria* and *Megachile rotundata* have been described following exposure to field doses of fungicides, including boscalid^29^. Around 60% of bumble bee queens died when exposed to blueberry plant material treated with Pristine® (pyraclostrobin 12.8% and boscalid 25.2%) at a rate of 1.6 kg/ha^30^. As is the case with chronic toxicity, hardly any of the described interactions of boscalid with other chemicals were considered when boscalid was first authorised.

A more careful examination of the long-term toxicity of boscalid for honeybees is appropriate based on the combination of these first toxicity results and the duration of the exposure of bees to boscalid. A re-examination may also be relevant for other molecules because the chronic toxicity of most pesticides entering the European market before 2014 was not evaluated within the pesticide authorisation procedure (Regulation EU 283/2013)^31^.

In a previous study we found only limited evidence for long-term toxicity of pure boscalid for honeybee larvae^32^, but its impact on adults remains to be investigated. Standard guidelines recommend a ten-day trial for chronic toxicity evaluation on adults^33^. Preliminary results (unpublished data) have shown that the toxicity of this product may appear only after periods longer than ten days. Repeated or prolonged exposure may also induce “time-reinforced toxicity” also called “cumulative toxicity” due to bioaccumulation of the product in the body or to other mechanisms^34^. Several different methodologies are described in the literature for detecting such cumulative toxicity^35–37^.

The main objectives of this study are (1) to evaluate the chronic toxicity of a commercial formulation of boscalid with a time-to-death approach: the study finishes only when the mortality rate in the control reaches 50%; (2) to check for evidences of cumulative toxicity using different methodological approaches. In addition, we carefully examined the kinetics of food consumption not only because this parameter is crucial to evaluating the real exposure, but also because it can provide insights about the physiological changes over the life of the bees.

## Results

### Food consumption and duration of the trial

**Figure 1:**
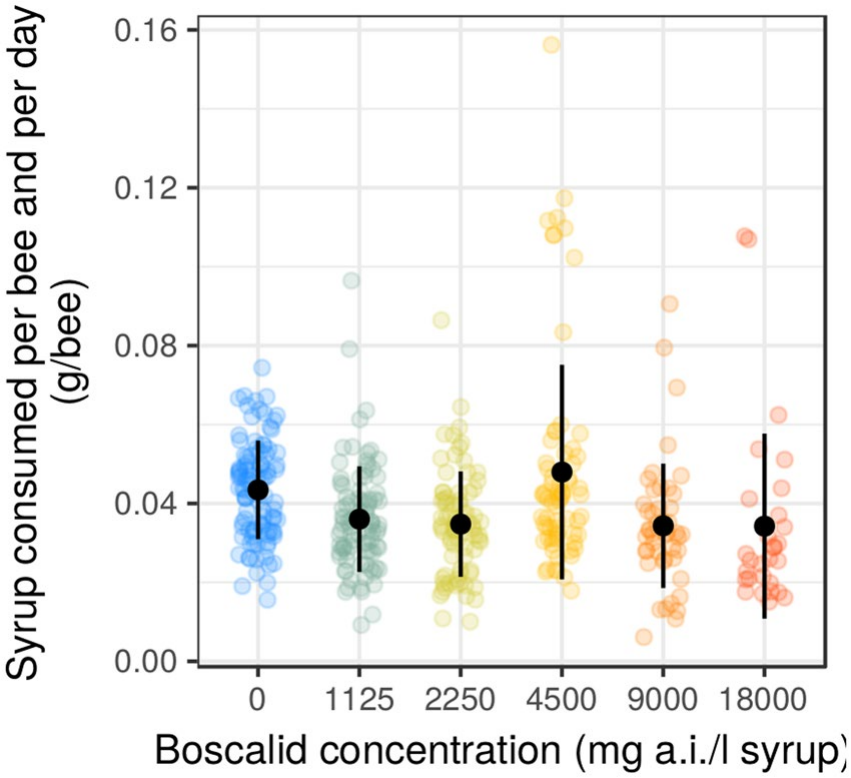
Daily syrup consumption at different boscalid concentrations. The black dots represent the mean value and the bars are the standard deviation. Each transparent coloured point represents the consumption per bee during one day in one cage. There is no significant difference of average consumption between treatments (Gaussian mixed model with cage as random effect: F_5,12.2_ = 2.289, p = 0.1104).

The average daily food consumption per bee over the duration of the trial (33 days) was 39 ± 1.7 mg bee^−1^ day^−1^, and no significant difference was found between the different treatments (Gaussian Mixed model with cage as random effect: F_5,12.2_ = 2.289, p = 0.1104). However, syrup consumption was not constant over time and showed clear differences between treatments once time was taken into account. Firstly, we observe a strong increase of individual food consumption in most cages when there were only 1 to 3 living bees left, with extreme values above 0.08 g syrup bee^−1^ day^−1^ (see Fig. 1 and Supplementary Material (Suppl. Mat.), Section 3.2.1). These extreme values may be partially due to the measurement error induced by syrup evaporation. The difference in syrup weight due to evaporation is divided by the number of living bees. Consequently, the relative importance of evaporation increases when there are only a few living bees left. Nevertheless, after correcting consumption for evaporation (Suppl. Mat., Section 3.2.2) we still obtained some large peaks of consumption when there were only a few living bees left.

**Figure 2:**
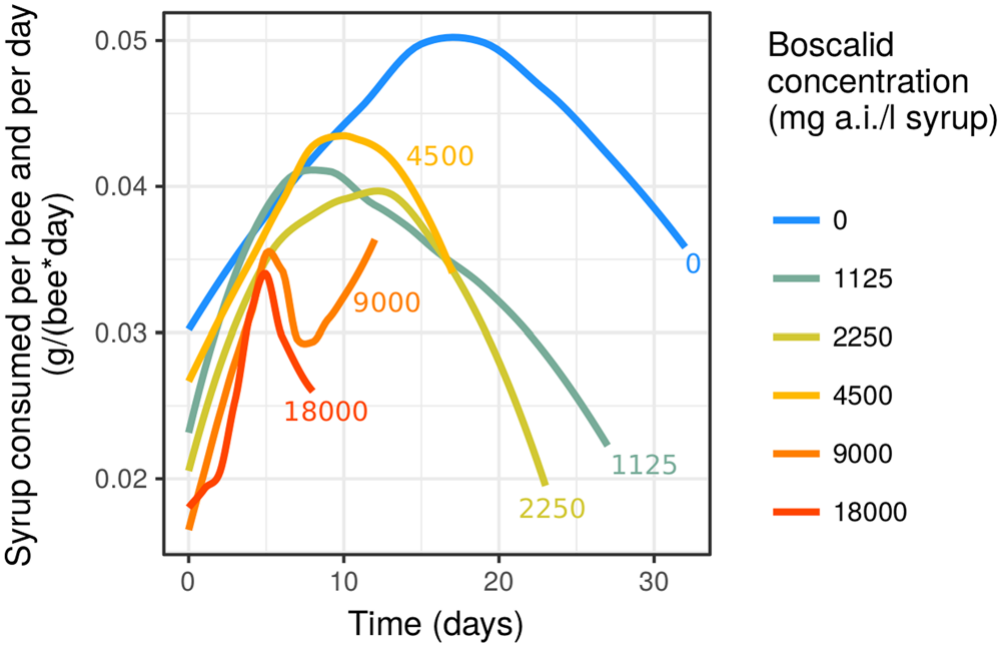
Daily syrup consumption over time until 50% mortality is reached in each cage. The lines for each boscalid concentration are loess trends (locally weighted polynomial regression). See Suppl. Mat., Section 3.2.3 for detailed representation of observed consumption rates and statistical testing with a polynomial mixed model. The model show highly significant time x concentration and main concentration effects: the kinetics of consumption was different between treatments and the average consumption on a given day was also different.

Secondly, when we removed these outliers by only considering consumption data while a minimum of 5 bees remained per cage, we observed that consumption was still not stable over the duration of the test (Fig. 2). An inverted “bell” shape was observed in the control, with individual consumption increasing to a maximum around days 17–18 of the test and then decreasing slowly until the end of the observation period (33 days). A similar pattern in consumption was observed for the bees at the different concentrations, but with differing kinetics: at higher concentrations, the maximum was reached sooner and the increase and decrease were steeper (Fig. 2). At a given point in time, bees tend to significantly reduce their consumption when Cantus® contaminates the food, the reduction being greater at higher concentrations (These visual differences were supported by statistical testing with a polynomial mixed model, see Suppl. Mat., Section 3.2.3).

### Mortality rate and toxicological endpoints

**Figure 3:**
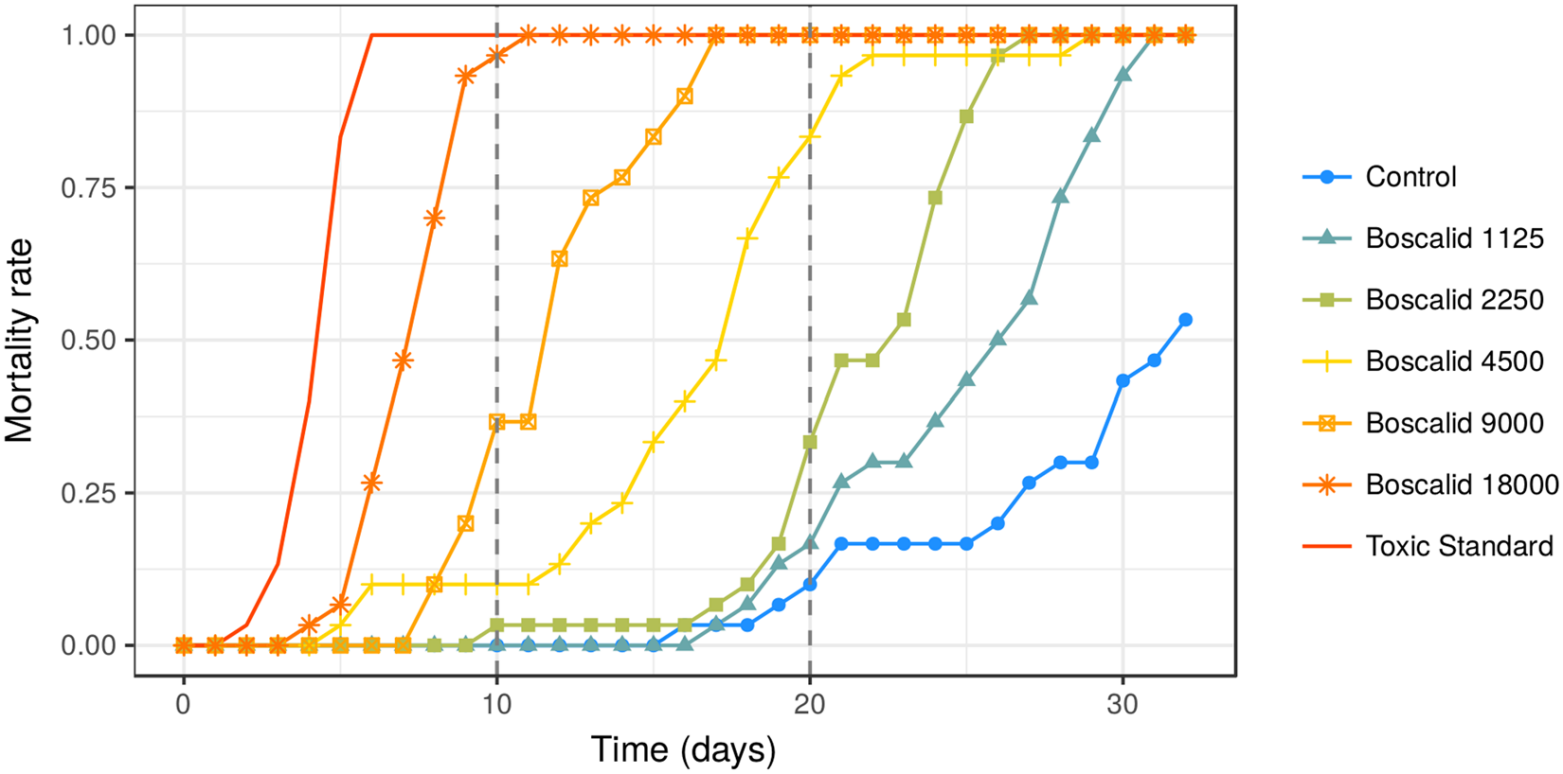
Observed mortality rate over the duration of the test. N = 30 bees per treatment, except for boscalid 9,000 mg/l, n = 29. Dashed vertical lines indicate ten days, i.e. duration of the test currently proposed by international standards, and 20 days, i.e. time at which the mortality in the control reached 15%. Current international standards (ten days) propose as validity criteria up to 15% mortality in the control group. Toxic standard was dimethoate at 1.5 mg/l.

The mortality rates were in agreement with the validity criteria established by OECD draft test guidelines for the evaluation of the toxicity of pesticides over ten-days^33^: (1) the average mortality for the controls was lower than 15% at day 10 of exposure and remained so up to day 20; (2) the average mortality in the toxic standard was higher than 50% at day 10 (Fig. 3).

Before day 10 - i.e. the normal duration of a chronic test - observed mortality was low. A mortality rate higher than 50% was only observed at the highest Cantus® concentration (18,000 mg boscalid/l) from day 8 onwards. On day 10, mortality had reached almost 100% at the highest concentration, but remained below 50% for all other treatments and close to 0% in most cases. On day 20, when mortality in the control had reached 15%, mortality for the two highest doses (9,000 and 4,500 mg boscalid/l) was 100%. On day 31, the mortality rate of all Cantus® treatments (including 2,250 and 1,125 mg boscalid/l) had reached 100% while that for the control was just below 50%.

**Figure 4:**
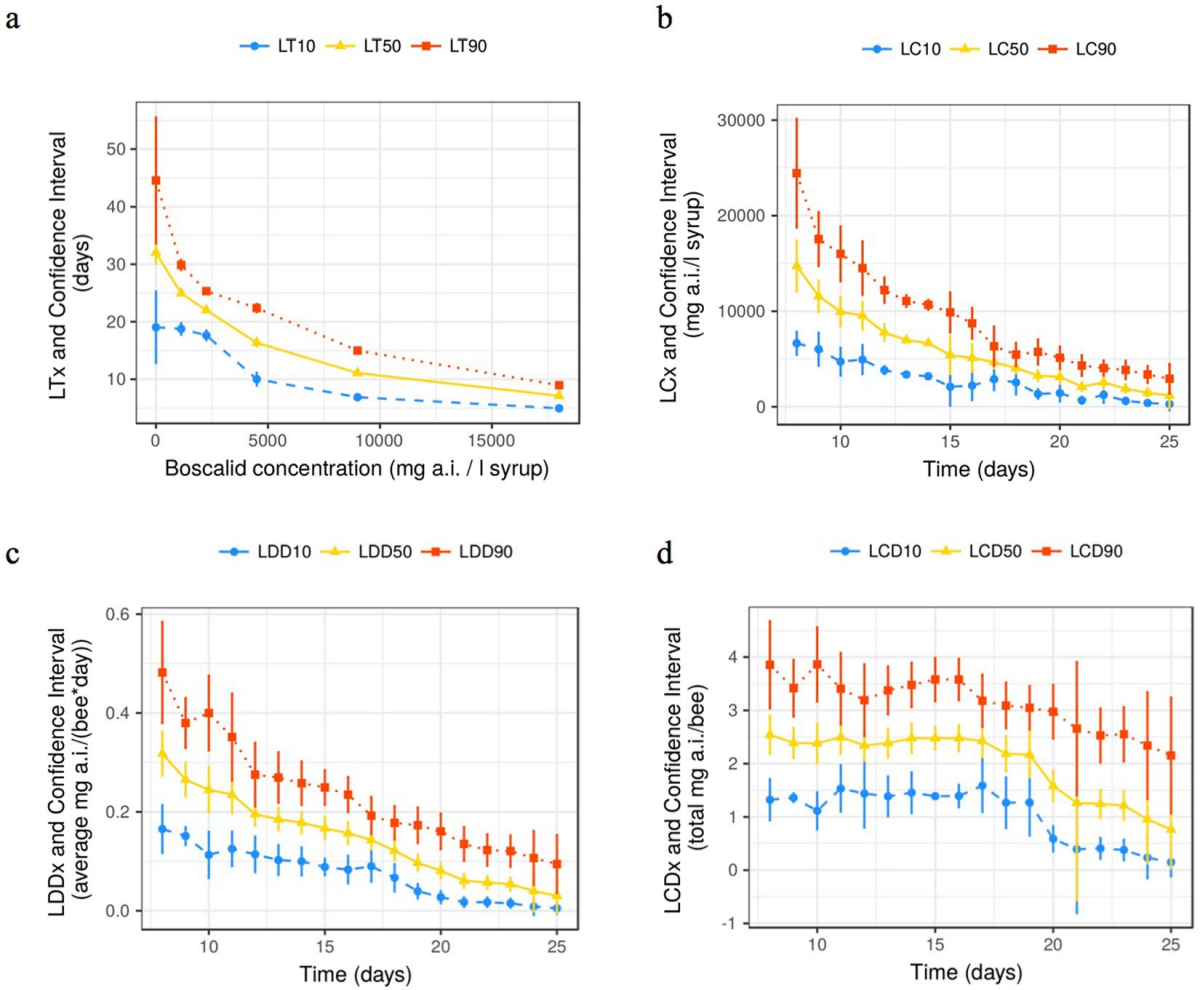
Representations of LTx vs. Concentration (**a**), LCx vs. Time (**b**), LDDx vs. Time (**c**) and LCDx vs. Time (**d**). All graphs represent the toxic endpoint for 10%, 50% and 90% mortality estimated with three parameters Weibull 2 models. See Table 1 for meaning of the abbreviations. See Suppl. Mat., “raw_results” directory to obtain the raw numbers used in these graphs.

The median lethal time (LT_50_) decreased as the concentration increased (Fig. 4a): it was 7 days (95% CI: 6.89, 7.37) at the highest concentration (18,000 mg/l) and 25 days (24.38, 25.47) at the lowest tested concentration (1,125 mg/l, Fig. 4a). The LT_50_ of the latter group was significantly lower than the 32 days (29.84, 34.16) of the control group. The LT_50_ of the toxic standard was 4 days (3.87, 4.4).

Figure 4b–d, summarise the LC_50_, LDD_50_ and LCD_50_ values from day 8 to day 25 of the trial (see values in Supplementary Material and data files in the “raw_results” directory). LCx and LDDx decreased over time. LC_50_ started at 14,728.73 mg/l (12,055.50, 17,401.95) on day 8 and was 1,174.25 mg/l (−150.51, 2,499.01) on day 25. In terms of individual dose, LDD_50_ was 0.32 mg boscalid bee^−1^ day^−1^ (0.27, 0.36) on day 8, and decreased to 0.03 mg boscalid bee^−1^ day^−1^ (−0,004, 0.064) on day 25.

The median lethal cumulative dose (LCD_50_) showed a plateau around 2.4 mg boscalid bee^−1^ until day 17, when LCD_50_ started to decrease, reaching 0.76 mg boscalid bee^−1^ (0.42, 1.480) on day 25 (Fig. 4d).

### Cumulative toxicity - first approach: log-log linear regressions

The slopes of all log-log linear regressions were significantly lower than −1 (Suppl. Mat., Fig. 20) except for the slope of the regression between log (concentration) and log (LT90), which was only at the margin of statistical significance (CI: −3.370, −0.995). The time-concentration relationships diverged clearly from Haber’s law and pointed to an increased toxicity for long time exposure.

**Figure 5:**
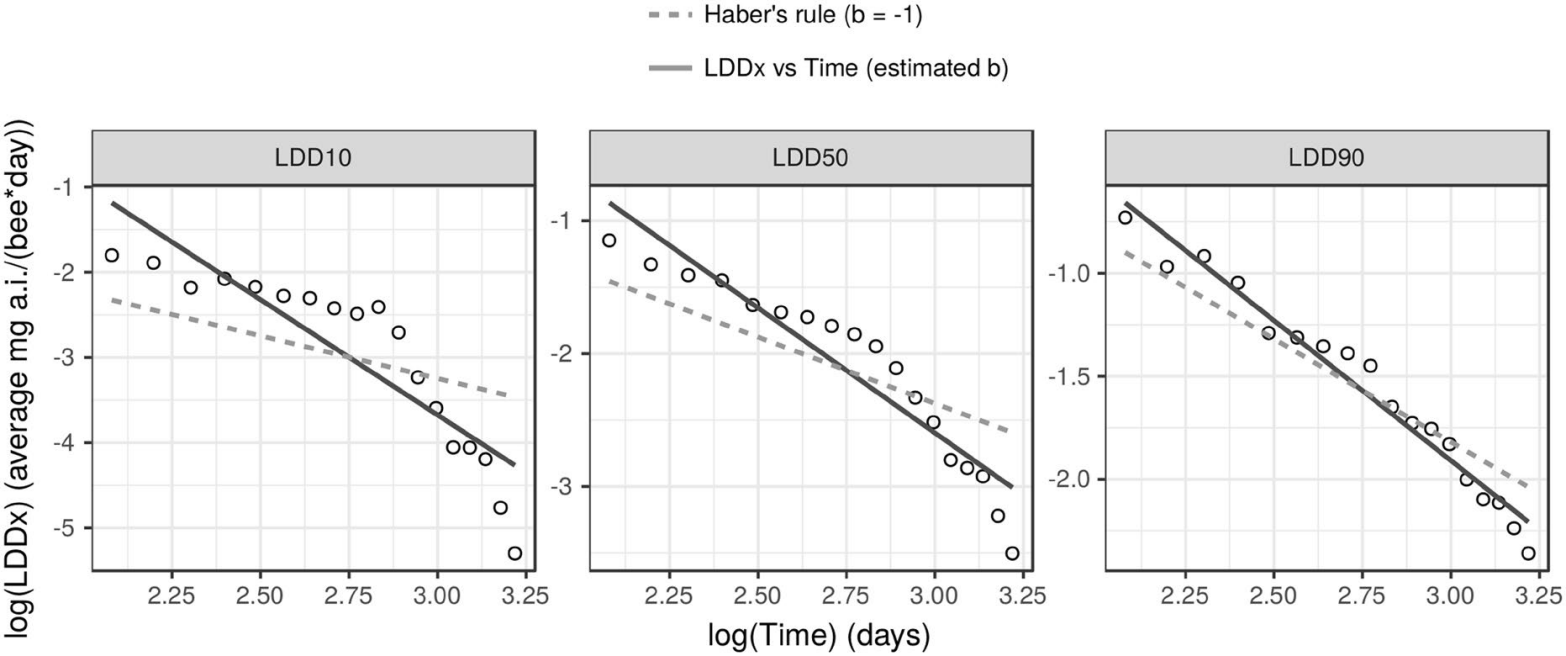
Log-log regression of LDDx vs. Time compared to the regression line expected in the absence of cumulative toxicity. The dashed lines show regression lines with a fixed −1 slope (the models estimate only the intercepts). This is the expected relationship under Haber’s law i.e. without cumulative toxicity. The slopes of the observed linear regression are significantly lower than −1, which implies some level of cumulative toxicity. However the log-log relationship is clearly not linear at least for the lowest mortality rates. The representation of the log-log regressions for the other toxic endpoints can be found in Suppl. Mat., Section 5.1. along with confidence intervals of the slopes (Fig. S20).

However the slope is only a rough summary of this relationship. Plotting the data revealed that the log-log relationship was not always linear and showed interesting patterns (see for example Fig. 5 and Suppl. Mat., Section 5.1 for more details).

More precisely, the log (LDDx) vs log (Time) relationship seemed to follow Haber’s rule closely up to day 17–18 with a slope around −1. At this point the slope abruptly decreased and the relationship clearly deviated from Haber’s rule (Fig. 5). This pattern was more obvious for lower levels of mortality (LDD_10_, LDD_20_, etc) and tended to disappear at higher levels of mortality (LDD_90_). For the LDD_90_, the points were almost perfectly aligned on a straight line without any inflection point. However, even for the LDD_90_, where the observed regression line seemed to be close to the theoretical Haber’s line, the slope was significantly smaller than the expected −1 (estimate = −1.359 with a 95% confidence interval of (−1.492, 1.226)).

This pattern was less marked on the log (LCx) vs log (Time) graph (Suppl. Mat., Figs 24 and 25) and not visible on the log (Concentration) vs log (LTx) graphs (Suppl. Mat., Figs 22 and 23); however, the later graphs were based on five points only, which was probably not enough to visualise this non-linear relationship.

### Cumulative toxicity - second approach: cumulative doses

**Figure 6:**
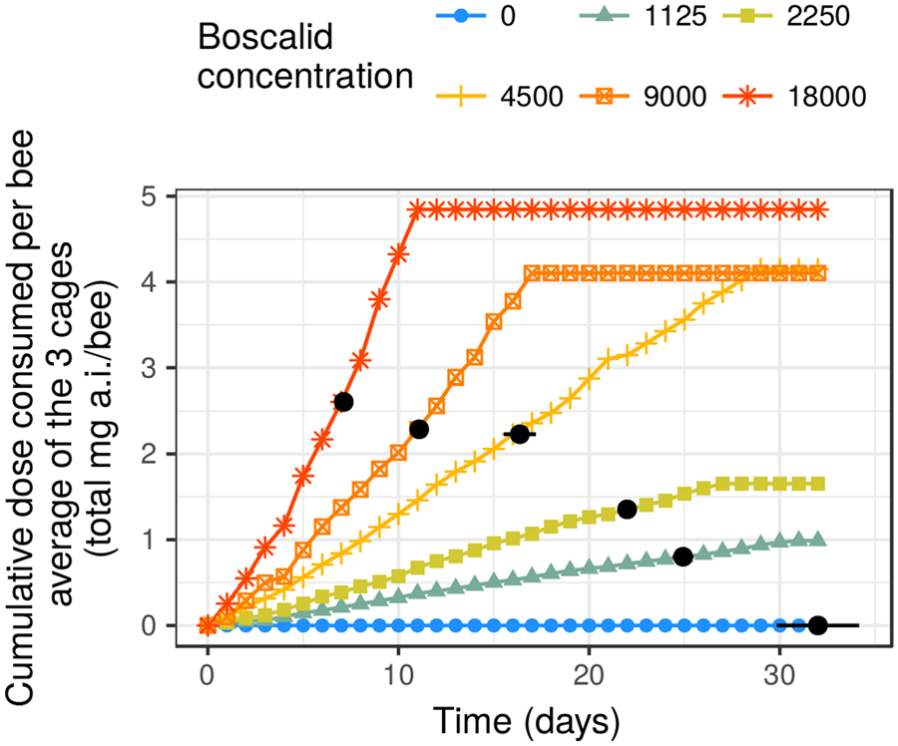
Cumulative dose consumed per bee for each treatment (concentration) over time. The black dots indicate the estimated values of LT_50_ (with 95% confidence intervals) for each concentration. Without cumulative toxicity, the black dots should appear horizontally at approximately the same level of cumulative dose or aligned vertically above the control LT_50_ for the two lowest concentrations.

The cumulative dose necessary to reach 50% mortality was similar (approximatively 2.5 mg a.i./bee) for the three highest-concentration treatments (18,000; 9,000 and 4,500 mg a.i./l syrup – see Suppl. Mat., Fig. 6). The same level of mortality was reached with significantly lower cumulative doses for the two lowest-concentration treatments: 1.38 mg a.i./bee for a concentration of 2,250 mg a.i./l syrup and 0.84 mg a.i./bee for a concentration of 1,125 mg a.i./l syrup (Fig. 6 and Suppl. Mat., Section 5.2). While the time to reach 50% mortality (Fig. 6: black dots - LT_50_) was longer for the lowest concentrations, the cumulative dose necessary was much lower than what we observed for higher-concentration treatments.

Without cumulative toxicity (under Haber’s rule) the bees should be exposed to the same cumulative dose to reach the same level of mortality. Again, these results point to an increased toxicity in the context of a long-term exposure to Cantus®.

## Discussion

### Syrup consumption

Despite having used a standardised methodology, syrup consumption was highly variable within the cages, between the cages and over time. We observed a non-constant individual daily syrup consumption over the duration of the trial, with high consumption values when only a few bees were left in the cage (1 to 3 bees). These extreme values may be in part due to syrup evaporation, which is why it would be worthwhile to include this parameter as a measurement to be performed during chronic toxicity testing. In contrast to our results, Arnold^38^ observed lower levels of consumption when bees were isolated. We also clearly observed a non-constant consumption level over time and a difference between the treatments both in terms of kinetics (the first peak of consumption appeared at a different time) and in term of absolute consumption (the food intake was lower when the concentration was higher whatever the time). In contrast to our observations, a constant daily consumption per bee and no treatment effect were reported in a similar experiment testing chronic mortality rates of imidacloprid and deltamethrin^39^. However, our results show that real consumption differences may be masked when the data are analysed with simple statistical approaches because of complex non-linear consumption *vs* time relationships and differences in kinetics (time x treatment interactions). In addition, the difference in food consumption found between the different doses and over time, mainly at the beginning of the test, could be linked to the perception/taste of the substance by the bees as is the case with other products^40–42^. To our knowledge, no repellent effect has been described for boscalid, but additional trials could be performed to evaluate the preferences of bees between diets containing boscalid or without it.

The variation of syrup consumption over the trial and the food distribution among bees are realities whose impact needs to be considered during the interpretation of toxicity results, as they will have an impact on dose calculation, but also on the implementation of methodologies proposed for regulatory purposes, i.e. cumulative toxicity^36^.

### Toxicity of Cantus^®^ for bees

The mortality rate of the bees in the Cantus® treatments was significantly higher than that of the control group. The chronic toxicity of this pesticide in terms of concentration (LC_50_) was 1,174 mg boscalid/l on day 25. This concentration is far from the highest residue value described in the literature for boscalid in pollen (26 mg/kg)^16^, but lies in the lower range of concentrations applied in the field. In addition, the effects measured here are mortality rates; we could expect sublethal effects at even lower concentrations. Both LC_50_ and LDD_50_ decreased in time, their values being reduced by approximately 90% from day 8 to day 25. Similar results have been found in other studies, mainly with insecticides^39,43–52^. The main differences between our study and others were the level of toxicity described and the order of magnitude of the concentrations tested. These tested concentrations are a reflection of the amount of residues bees are exposed to in natural conditions.

Standard methodologies currently prescribe the testing of chronic toxicity over a period of ten days^33^. These standard methodologies are themselves inspired by previous toxicological studies evaluating chronic effects on bees after 10–14 days exposure^44,50,51^. This duration seems adequate for testing pesticides with high to moderate acute toxicity for bees. The effects we observed here, however, would not have been detected over a duration of ten days. Pesticide exposure longer than ten days is not an unrealistic situation for bees, given that boscalid has been found to contaminate food resources over months^11^. This is not surprising considering that boscalid is systemic, persistent and authorised for a large list of uses, even during the flowering period of many crops^53^. For this reason, we think that a time-to-death approach might be more appropriate for evaluating the risk of chronic exposure to a pesticide. The estimation of the LTx as toxicological endpoint is useful for comparing the effect of chronic exposure to different doses and makes in possible to evaluate the impact of stressors on the lifespan of bees. In our case, even the lowest concentration tested significantly reduced the time to reach 50% mortality. An additional advantage of such an approach is that it can help detect a potential cumulative toxicity.

### Cumulative toxicity

A first clear indication of the existence of cumulative toxicity of Cantus® can be inferred from the relationship between the Lethal Cumulative Dose (LCDx) and Time. The plateau observed in Fig. 4d until day 17–18 indicates that a certain number of bees managed to detoxify the ingested pesticide: whatever the duration of repeated exposure, the same cumulative amount of pesticide was necessary to kill 50% of the bees. From days 17–18 onwards, a decreasing total amount of pesticide was sufficient to kill the same number of bees. Their detoxifying capacity seemed to be exceeded, or the accumulation of boscalid in the bees’ bodies was enough to trigger toxic effects which increased with time. This time-increased-toxicity and sudden change in toxicity profile around 17–18 days were confirmed by several other statistical approaches: (1) the log-log regressions testing whether the relationships between concentration or dose and time followed Haber’s rule^54^ and (2) the so-called EFSA protocol (comparison of cumulative doses to reach 50% of corrected mortality between concentrations)^36^.

Moncharmont *et al*.^39^ observed a delayed toxicity of the insecticide imidacloprid in bees and argued that the effects could be due to age-dependent sensitivity or to the accumulation of the compound in the body. The dynamic of syrup consumption in the control also suggested a naturally occurring change in the physiology of the bees even without consumption of Cantus®: at days 18–19 of their life, bees in the control achieved a maximum syrup consumption, while the mortality rate was slowly increasing. The sudden change of toxicity around 17–18 days could then be explained by higher sensitivity due to the natural ageing process, by physiological fatigue due to repeated exposure to the pesticide during their life, or by a combination of the two processes. The similar patterns of food consumption observed for the different concentrations, but with maximums at earlier dates for higher concentrations, might indicate faster physiological ageing for bees exposed to the pesticide and suggest that ageing alone is not a sufficient hypothesis to explain the patterns observed. The effects of xenobiotics on bees involve responses of detoxification and oxidative and general stress leading to an increase in the insect’s energetic metabolism^55^. These may lead to physiological wear-out and reduction of vitellogenin production, thus shortening the lifespan and immuno-competence of bees^17,56–59^. We did not supply any dietary protein during the test. The already limited detoxification capacity of bees^60^ may therefore have been even more restricted when the bees exhausted their capacity for protein synthesis due to a lack of essential aminoacids^61^.

The toxicity and the sudden change in toxicity could also be explained by mechanisms other than ageing and physiological fatigue. For example, as boscalid is moderately lipophilic (Kow = 2.96)^25^, it could have a certain tendency to accumulate in the bees’ fatty tissue (i.e. bioconcentration). The products contained in Cantus® or their metabolites could accumulate in the bees’ fatty tissue from the onset of exposure up to a level of saturation, when these chemicals could target other tissues, accelerating the toxic effect of these compounds if exposure is prolonged. Unfortunately we did not evaluate the residues of boscalid and metabolites in dead bees. This would have provided very useful information about contamination in the bees and the accumulation process in their bodies according to the concentrations used^47^. The lower food intake when the syrup was contaminated by the pesticide may also have contributed to the weakening of the bees and decreased their capacity to handle the toxicity in the long term. Finally, it must be stressed that the toxicity observed here may have been induced by boscalid itself but also by the co-formulants present in Cantus® or interactions between all these xenobiotics^62^. Limited information is available on co-formulates because few ingredients are revealed in the description of the product. Phyto-pharmaceutical companies regard the composition of their formulation as trade-secret.

### Cumulative toxicity - methodological discussion

The time-to-death approach used here generated a large amount of data that allowed us to explore different methodologies for testing for cumulative toxicity. The main advantage of the protocol proposed by EFSA is that it is less costly: after an acute toxicity test of 48 h, one is required to perform a new test and monitor mortality for only two concentrations (the LC_50_ and ¼ of this concentration) up to 50% mortality and then test whether the cumulative lethal doses are equal. However, in our case, the toxicity after 48 h was too low to estimate the LC_50_ (no acute toxicity). After ten days, the estimated LC_50_ was approximately 10,000 mg/l and could have been compared with a 2,500 mg/l treatment. Instead of running a new test, we monitored mortality for all our initial concentrations until the control reached 50% mortality. Using these data, we were able to show that the cumulative doses to kill 50% of the bees at a concentration of 9,000 mg/l (the concentration closest to the LC_50_) and 2,250 mg/l are significantly different, supporting the hypothesis of cumulative toxicity with an approach close to the simple EFSA protocol. However, using only two concentrations would have masked some of the most interesting patterns observed in this study, which could help us to understand the toxicological effect of the pesticide. The log-log regression between Lethal Dose and Time revealed a clear breaking point in the toxicological effect after 17–18 days which would have gone unnoticed with simpler approaches. The log-log regression between Concentration and Lethal Time was also less interesting for the same reason: the lower number of points (i.e. the number of different concentrations tested) makes such subtle patterns difficult to spot. We also showed that simply estimating the slope of the log-log regression, while useful, was only a rough summary: it was necessary to plot the data to check if we had a simple, constant, deviation from Haber’s rule or a more complex relationship between toxicity and time. The Lethal Dose should also be preferred to the Lethal Concentration in log-log regressions, because the latter does not take into account the differences in food intake between treatments. However, Lethal Concentration estimates are still useful for comparing the lab results with the concentrations observed in beehive matrices or applied in the field by farmers.

## Conclusion

In conclusion, we showed that at field application rates, Cantus® (500 g boscalid/kg) leads to chronic toxicity in honeybees that would have remained undetected with current proposed methodologies for pesticide risk assessment. This fungicide significantly reduced the lethal time for all concentrations tested. Furthermore, a cumulative toxicity potential was detected particularly after 17–18 days of exposure. All in all, we recommend a time-to-death approach rather than fixed-duration studies for exploring the chronic toxicity effects of pollutants that are present over long periods in field conditions.

## Materials and Methods

### Test substances

We tested five concentrations of boscalid: 18,000, 9,000, 4,500, 2,250 and 1,125 mg a.i./l. The test substances were dissolved in a 50% w/v sucrose solution provided as food to the bees *ad libitum*. We used the commercial formulation Cantus® (BASF, 500 g/kg solid boscalid) because the pure active substance has a low water solubility (4.6 mg/l). We had tried to perform earlier experiments with the pure active ingredient, but could not be sure of the exposure due to its low solubility and its flocculation. The concentrations were chosen based on previous studies and the potential concentrations used in the field. Application rates in the field range from 250 to 1,880 g a.i./ha^27^. Based on the maximum application rate and on the typical spray rate per hectare in Belgium (100–200 l/ha), a concentration of 18,800–9,400 mg/l would be in the upper range of concentration expected to be found in the field, while 1,125 mg/l would be in the lower range. Dimethoate (Perfektion®, BASF, 400 g/l dimethoate) was used as toxic standard at 1.5 mg a.i./l as recommended by OECD standards^33^ to ensure that bees were well exposed to the toxicants.

### Bees

Three frames containing capped cells with emerging bees were collected from three healthy queen-right colonies with queens of different origin. They were left in an incubator at 32.8 °C ± 1 °C and 60% ± 20% relative humidity. One-day-old worker bees were transferred without anaesthesia into cages in groups of ten bees per cage.

### Experimental conditions

The bees were kept in cardboard cages with a mobile plastic window, in darkness, within an incubator at 32.8 °C ± 1 °C and 60% ± 20% relative humidity for the duration of the test. They were fed *ad libitum* with a syrup (sucrose solution with different concentrations of test substances) provided through 2 ml plastic syringes with the tip removed.

### Treatments and data gathering

At the beginning of the test, each treatment group was composed of three replicates each containing ten bees. In one cage one of the ten bees died before the start of the experiment (i.e. before the contaminated syrup was provided). The initial number of bees was considered to be nine, instead of ten, in this cage. Bee mortality was recorded every day at approximately the same time as the syringes were changed. Dead bees were removed at each assessment. Syringes containing freshly prepared syrup were replaced every day and weighed just before administration and again the following day to estimate the quantity of syrup consumed by the bees. This consumption value was divided by the number of living bees to obtain the consumption per bee and per day. The test lasted until mortality in the control group reached 50%, instead of stopping the test after ten days as recommended by OECD guidelines^33^.

### Data analysis

All analyses were performed with R^63^ mainly with drc package^64^ for dose response curves (Weibull models) and toxicological endpoints estimations, the lme4 package^65^ for mixed models and the multcomp package^66^ for post-hoc multiple comparisons. All datasets, R code and detailed data analyses are provided as a public figshare repository (https://figshare.com/s/865e87feaad34c095bbd).

### Data analysis – Syrup consumption

We first compared the daily syrup consumption per bee between the different treatments (different Cantus® concentrations) without taking the time into account. We used a Gaussian mixed model with the cage as random effect and the treatment as fixed effect. We then analysed how syrup consumption changed over time for each treatment. We used graphical tools such as loess (locally weighted polynomial regression) to visualise the trends in the noisy data and used mixed models to check if the observed patterns were statistically significant. We used the cage as random effect and the treatment, time (second-order polynomial) and their interactions as fixed effects. The evaporation of syrup was also measured for a subset of the days in cages without bees or with only dead bees (see Suppl. Mat., Section 3.1).

### Data analysis – Toxicological endpoints

We computed four toxicological endpoints (and their 95% confidence intervals): Lethal Time (LTx), Lethal Concentration (LCx), Lethal Dietary Dose (LDDx) and Lethal Cumulative Dose (LCDx) (Table 1). The LTx was computed for each concentration and the LCx, LDDx and LCDx were computed for each day of the test between day 8 and day 25 (outside this range, the mortality was either too low or too high). All “x” levels of mortality between 10% and 90% in 10% increments were calculated.

**Table 1:** Definitions and abbreviations of the toxicological endpoints.

Variable	Abbreviation	Definition	Units	Model
Concentration	C	Quantity of test product within the syrup provided as food to the bees	mg a.i./l syrup	
Dose	D	Quantity of test product actually consumed by the living bees through their food (syrup) on a given day. The calculations were based on syrup consumption, concentration of the test product in the syrup, syrup density (1.23 g/ml) and the number of bees still alive	mg a.i. bee^−1^ day^−1^	
Daily Dietary Dose	DD	Average of the doses consumed during a given day and all preceding days	average mg a.i. bee^−1^ day^−1^	
Cumulative dose	CD	Sum of the doses consumed during a given day and all preceding days	total mg a.i. bee^−1^	
Lethal Time	LTx	Number of days of exposure to cause x% mortality at a given concentration	days	Based on the Weibull 2 model: Mortality vs Time
Lethal Concentration	LCx	Concentration needed to cause x% mortality after a given period of exposure	mg a.i./l syrup	Based on the Weibull 2 model: Mortality vs Concentration
Lethal Dietary Dose	LDDx	Average daily dose consumed per bee needed to cause x% mortality after a given period of exposure	average mg a.i. bee^−1^ day^−1^	Based on the Weibull 2 model: Mortality vs Daily Dietary Dose
Lethal Cumulative Dose	LCDx	Cumulative doses consumed per bee from day 0 needed to cause x% mortality	total mg a.i. bee^−1^	Based on the Weibull 2 model: Mortality vs Cumulative Dose

To estimate these toxicological endpoints, we chose a three-parameters Weibull 2 model (*sensu Ritz*)^67^ to model the relationship between uncorrected mortality and time, concentration or dose. This three-parameters-sigmoid model fixed the higher asymptote (i.e. the mortality is 100% for an infinite dose) and estimated the lower asymptote (i.e. the mortality in the control). The toxicological endpoints were then calculated relative to these two asymptotes. The LCx, LDDx and LCDx estimates were therefore corrected for mortality in the control. For LTx we used the same approach but the lower asymptote in this case estimated the mortality at D0, which was zero. Hence, the LTx values as we calculated them here were not corrected for mortality in the control. See Suppl. Mat., Section 7, for more details.

### Data analyses – Cumulative toxicity estimation

Most of the methods used to evaluate the potential of cumulative toxicity (or time reinforced toxicity) were based on Haber’s rule^54^, which states that the product of exposure concentration (*C*) and exposure duration (*t*) leads to a constant toxic effect. In other words, if there is no cumulative toxicity, when the concentration (or dose) is divided by two the time of exposure to reach the same level of mortality should be doubled. Haber’s rule is a specific case of the Druckrey-Küpfmüller model, for which the exponent (*b*) has an absolute value of 1^34^.

1 $$C{t}^{b}={constant}$$

We used two different approaches to test if Cantus® induced time-reinforced toxicity: (1) log-log linear regression between concentration or dose and time to estimate the slope b from Eq. 1 (Eq. 1)^35^; (2) comparison between treatments of the cumulative dose needed to reach 50% mortality as proposed by the European Food Safety Authority (EFSA)^36^.

For the first approach, we estimated the slope (and 95% Confidence Intervals) of three types of simple linear regressions: (1) log (Concentration) vs log (LTx); (2) log (LCx) vs log (Time); (3) log (LDx) vs log (Time). Should cumulative toxicity exist, these slopes would be significantly smaller than −1. This was repeated for each level of mortality between 10% and 90% in 10% increments.

For the second approach, the EFSA^36^ has suggested evaluating the LC_50_ at 48 h and then running a test with two treatments: one corresponding to the estimated LC_50_ value and another with ¼ of this concentration. The cumulative dose needed to reach 50% mortality in each treatment is then compared. These cumulative doses should be equal if there is no cumulative toxicity.

We did not observe mortality in any treatment at 48 h, but we used a similar idea by comparing the cumulative dose needed to reach 50% mortality in our five concentrations (with two-fold dilutions). We used a one-way ANOVA (each cage in a dose was a replicate) and all pair-wise post-hoc comparisons (multcomp package^66^ to test for differences. For this part only we used corrected mortalities based on Abbott’s formula^68^.

A third approach proposed by Miller^35^ provided unconvincing results which are not detailed here (See Section 5.3 in the Suppl. Mat. for the details).

### Data availability

All datasets, R code and detailed data analyses are provided as a public figshare repository (https://figshare.com/s/865e87feaad34c095bbd) and the Supplementary Material contain enough detail to understand the analyses performed.

## Electronic supplementary material

Supplementary Material
