# Supplementary material for: Time-to-death approach to reveal chronic and cumulative toxicity of a fungicide for honeybees not revealed with the standard ten-day test
Source: Sci Rep. 2018 May 8;8:7241. doi: 10.1038/s41598-018-24746-9 (PMC5940668; doi:10.1038/s41598-018-24746-9)
Supplement: Supplementary file 1 — Supplementary Material [file 41598_2018_24746_MOESM1_ESM.pdf]

Time-to-death approach to reveal chronic and cumulative toxicity of a fungicide for honeybees not revealed with the standard ten-day test

Noa Simon
Gilles San Martin
Etienne Bruneau
Louis Hautier

01 March 2018

Contents

1 Introduction - Context **3**
1.1 Context . . . 3
1.2 Aims of this project . . . 4
1.3 Methods and data analysis . . . 4

2 Raw data and computations **6**

3 Syrup consumption and evaporation **8**
3.1 Evaporation data . . . 8
3.2 Consumption of syrup between doses and over time . . . 10
3.2.1 Kinetics of the consumption without evaporation correction . . . 10
3.2.2 Kinetics of the consumption with evaporation correction . . . 11
3.2.3 Kinetics of the consumption up to 50% of mortality . . . 13
3.2.4 Average consumption (without taking the time into account) . . . 17
3.3 Conclusions . . . 20

4 Kinetics of the toxicity : LTx, LCx, LDDx, LCDx at different time or concentration **21**
4.1 Raw mortality rates . . . 21
4.2 LTx vs Concentration . . . 22
4.3 LCx vs time . . . 22
4.4 LDDx vs Time . . . 23
4.5 LCDx vs Time . . . 23
4.6 Conclusion . . . 24

5 Testing for cumulative toxicity (= "time reinforced toxicity") **25**
5.1 Log-log relationship between concentration and time . . . 25
5.1.1 log(Concentraton) vs log(LTx) . . . 28
5.1.2 log(LCx) vs log(Time) . . . 29
5.1.3 log(LDDx) vs log(Time) . . . 30
5.1.4 log(LCDx) vs log(Time) . . . 31
5.2 Comparing cumulative dose between concentrations (EFSA protocol) . . . 33
5.2.1 With raw mortalities . . . 33
5.2.2 With corrected mortalities . . . 35
5.3 Estimation of α and β . . . 38

6 Comparison of the 4 types of models **43**

6.1 Main results . . . 43
6.2 Lethal concentrations (LCx) at each time . . . 44
6.3 Lethal dietary dose (LDDx) at each time . . . 48
6.4 Lethal cumulative dose (LCDx) at each time . . . 52
6.5 Lethal time (LTx) at each concentration . . . 56

7 Comparison of models with 2 or 3 parameters using corrected mortality or not 60

8 References 61

9 Session Information 63

---

1 Introduction - Context

1.1 Context

The full datasets, R code, and raw results output related to this document are available in a public figshare repository : https://figshare.com/s/865e87feaad34c095bbd

This report presents the detailed analyses performed for the following paper : Chronic and cumulative toxicity for honey bees of the fungicide boscalid revealed with a time-to-death approach but not with the standard 10 days test
by Noa Simon-Delso, Gilles San Martin, Etienne Bruneau, Louis Hautier

The context of this study is twofold :

1. the evaluation of the methodologies used to assess the (chronic) toxicity of pesticides on adult bees
2. a specific fungicide (boscalid) whose toxicity for honey bees has started to attract attention in recent years

Methods to estimate toxicity of pesticides on bees

- It is important to find international agreements on the methodologies in order to make the ecotoxicological studies used for risk assessment comparable between labs and between products.
- However existing methods might need evaluation, validation and improvement.
- It might be argued that it is better (at least from a regulatory point of view) to have a standardized, stable, but maybe imperfect method than to gradually improve existing methods with a continuously changing protocol that makes the studies not comparable. However, even in that case, it might be useful to understand the limitations of existing protocols.

Many questions can arise, in particular concerning oral chronic toxicity tests :

- Is the standard duration of 10 days for chronic tests on adults enough to detect chronic troxicity ?
- How to detect cumulative toxicity or "time reinforced toxicity" caused by bioaccumulation or other mechanisms?
- Instead of a fixed time evaluation (eg mortality after 10 days) how can the kinetics of the toxicity improve our evaluation of the environmental risk of a pesticide ?
- How does the syrup consumption change over time and between treatments and what is the impact on the ecotoxicological results ?
- What is the impact of evaporation on the evaluation of the dose consumed by the bees ?

Boscalid

- Boscalid is a persistent, systemic and widely used fungicide.
- It is one of the most frequently found pesticides within beehives in Belgium.
- It has also been found in pollen pellets collected by bees very late in the season (October) at periods of the year where the pesticide is unlikely to be used in the field.

–> Hence, it seems that bees are frequently exposed and during extended periods to this pesticide. The question of its chronic toxicity is therefore legitimate.

- A former observative field study has shown a positive relationship between unexplained winter colony health problems and the fungicides load (mainly boscalid) found within the beehive.
- It is however only a correlation and it remained unclear whether boscalid itself has a direct, causal effect in the observed relationship.

A few lab studies have been recently performed that allow to explore a potential causal relationship between the presence of boscalid and the health problem observed in the field :

- Only the acute toxicity of boscalid on bees has been evaluated before commercialization
- A recent study has shown no (or limited) chronic toxicity of pure boscalid on honey bees larvae
- A recent study has shown a synergist effect of boscalid on a neo-nicotinoid. Previous monitoring studies done in the region to know the level of contamination of beehives did not detect many residues of neonicotinoids, but the sensitivity of the analyses used was not good enough. Therefore, bearing in mind the amounts of neonicotinoids sold in Belgium (official data, not shown), a potential exposure cannot be excluded.
- Several studies have shown no or limited synergist effect of boscalid on acaricides on honey bees or other arthropods

1.2 Aims of this project

1. Test the chronic oral toxicity of the fungicide boscalid (as a formulated product) on adult bees during a longer period than the classical 10 days duration of standard chronic tests
2. Test potential bioacculation of the product using different methodological approaches proposed in the literature
3. Compare the feeding rate over time, between treatments and evaluate the levels of syrup evaporation and their consequences on the results

1.3 Methods and data analysis

We used Cantus as commercial formulation of boscalid (50% w/w). Hence, the toxicity we observe here could be due to the co-formulants, not boscalid itself… It was not possible to use pure boscalid in this study because it was impossible to dilute it efficiently. In a former study on larvae, we used pure boscalid diluted in royal jelly that showed no or limited toxicity on larvae.

In the whole study, we make the distinction between

- `Concentration` which is the concentration of the product within the syrup provided to the bees. The units are `mg a.i./l syrup`.
- `Dose` which is the quantity of product really consumed by the bees through their food. It can be expressed as `average mg a.i./(bee*day)` for the daily average dose or `total mg a.i./bee` for the total cumulative dose.

We computed 4 types of dose or time to effect (mortality here) statistics.

- `LTx (days)`: Lethal Time based on the model : Mortality vs Time
- `LCx (mg a.i./l syrup)` : Lethal Concentration based on the model : Mortality vs Concentration
- `LDDx (average mg a.i./(bee*day))`: Lethal Dietary Dose based on the model : Mortality vs Average Dose consumed per bee and per day
- `LCDx (total mg a.i./bee)`: Lethal Cumulative Dose based on a model of Mortality vs Cumulative (total) Dose consumed per bee since D0

x represent the intended mortality rate (10%, 20%,…). NB : for the sake of simplicity, we will some time use "EDx" (Effect Dose) as a generic describer of LCx, LDDx, LCDx and even LTx, in line with the terminology used in the `drc` package.

We chose a 3 parameters Weibull 2 model (*sensu* Ritz et al. 2010) to model the uncorrected mortality vs time of concentration/doses. Three other types of models have been tested for all relationships : logistic, log-logistic and Weibull 1 (*sensu* Ritz et al. 2010), all with 3 parameters. The Weibull 2 and logistic models performed most of the time better (better fit, lower AIC) than the two other types of models. The Weibull 2 model was more stable than the logistic models for which the standard errors of the EDx were often

impossible to compute. Both models provided very similar estimates of EDx in any cases.
See section 6 for more details on the comparison of the 4 types of models and on the structure of these models. The `raw_output` directory provides the detailed results for the 4 types of models at each time, each concentration and for ED10 up to ED90 (by step of 10%).

The LCx, LDDx and LCDx estimates are corrected for the mortalities in the control because we use a 3 parameters model that estimates a parameter for the lower asymptote of the sigmoïd dose response curve (estimating the mortality when the concentration/dose is 0) and we compute the LCx, LDDx and LCDx relative to this asymptote.

For LTx we also use a 3 parameters model, however the lower asymptote in that case estimates the mortality at D0 which is always 0. Hence, the LTx values as we calculate them here are not corrected for mortality in the control.

Applying correctly a mortality correction might be difficult because the total number of bees are not exactly equal at the start of the experiment. Abbott's formula is applicable in such unbalanced cases. However binomial models require that the response variable (the % of dead bees - corrected or not) is a ratio of two integers which will most of the time not be the case after Abbott's correction when the sample sizes are unequal. This is the reason why we prefer to use 3 parameters models on uncorrected mortalities rather than the more classical 2 parameters models on corrected mortalities. NB : a 3 parameters model on uncorrected mortalities gives very similar results (in terms of Effect Dose estimates) than a 2 parameters model on corrected mortalities (see section 7 for more details and an empiric demonstration).

2 Raw data and computations

The data from the last day (D33) have been removed because there is no data about syrup consumption on this last day

Raw data :

- Treat : Treatment : CANTUS (= commercial name of the fungicide containing boscalid) - 2CNA (not used here) - TS (Toxic Standard) - CONTROL
- Rep : Replicate
- Conc (mg a.i./l syrup) : concentration of the a.i. in the syrup (sugar solution)
  The syrup is a 50% w/v sugar solution (e.g 1 kg sugar in 1 l water).
  This solution has a density of 1.23 g/ml for : 50% sugar with a purity of 100% at ~25°C, source.
  This Conc value is used to compute the LCx (Lethal Concentration x%).
- Eff (bees) : Total number of Bees on D0 (Effectif in French). Generally Eff=10. In few cases eff = 9 because some bees died btween the moment they were collected for the test and the moment to start the test (and there were no other available bees of the same age)
- Day (days): Experiment day starting at Day 0
- Alive (bees): number of living bees on day d
- Dead (bees): number of dead bees since day D0
- Abnormal (bees) : number of bees showing an abnormal behavior
- Weightt0 (g) : measure on day d of the weight of the new syrup seringue provided
- Weightt1 (g) : measure on day d+1 of the Weight of the old syrup seringue provided on day d

The rest of the columns are calculated on the basis of these first columns

- Time (days) : the day number starting at 0 (numeric version of Day)
- MortRate : mortality rate = Dead/Eff
- AliveRate : "living rate" = Alive/Eff (not used)
- Conso (g/cage): syrup consumption per cage on a specific day = Weightt0-Weightt1 (and = 0 when the data are not available, because all bees were already dead)
- ConsoBee : syrup consumption by the living bees on a specific day = Conso/Alive (g/living bee)
- Dose = (mg a.i./cage) Dose of ai ingested per cage by the living bees on a specific day (mg ai/cage) = `Conc*Conso/(1.23*1000)` (taking into account the syrup density)
- DoseBee (mg ai/living bee) = Dose of ai ingested per living bees on a specific day
  = `Conc*ConsoBee/(1.23*1000)`
- CumDose (total mg ai/ cage) = total amount of a.i. consumed by the bees taht are dead on day d since D0 = `cumsum(Dose) - Dose` for each combination of Treatment, concentration and replicate The dose consumed on day d is consumed by the living bees but not by the dead bees noted on that day. This is the reason why we remove the dose from dau d in the calculations. Otherwise said for day d, we compute the cumulative dose up to day -1 and the dose on D0 is 0
- CumDoseBee (total mg ai/ dead bee) : the same per bee = `cumsum(DoseBee) - DoseBee`. these values are used for the computations of LCDx (Lethal Cumulated Doses x%)
- MeanDose (average mg ai / (cage * day)): Average Dose in a whole cage consumed by the dead bees on day d since D0 (ie this is a kind of cumulated average). Basically this is = `CumDose/Day` for each replicate and each treatment. However the first value is not divided by 0 but by 1 to obtain an average dose = 0 at D0. And once the mortality reaches 100% the mean dose consumed remains constant.
- MeanDoseBee (average mg ai / (bee * day)) : The same but per bee i.e. average dose consumed per bees that are dead on day d since D0. Basically this is = CumDoseBee/Day with the same restrictions as MeanDose.
  MeanDoseBee is used to compute the LDDx (Lethal Dietary Dose x%)

```
##      Treat          Rep          Conc                Eff              Day           Alive      
##  2CNA   :495   Min.   :1   Min.   :    0.000   Min.   : 9.000   D0     : 36   Min.   : 0.00  
##  CANTUS :495   1st Qu.:1   1st Qu.:    1.208   1st Qu.:10.000   D1     : 36   1st Qu.: 2.00  
##  CONTROL: 99   Median :2   Median :  181.500   Median :10.000   D10    : 36   Median : 8.00  
##  TS     : 99   Mean   :2   Mean   : 2936.930   Mean   : 9.972   D11    : 36   Mean   : 6.33  
##                3rd Qu.:3   3rd Qu.: 2812.500   3rd Qu.:10.000   D12    : 36   3rd Qu.:10.00  
##                Max.   :3   Max.   :18000.000   Max.   :10.000   D13    : 36   Max.   :10.00  
##                                                                 (Other):972                  
##       Dead           Abnormal          Weightt0        Weightt1          Time   
##  Min.   : 0.000   Min.   : 0.000   Min.   :3.061   Min.   :2.889   Min.   : 0  
##  1st Qu.: 0.000   1st Qu.: 0.000   1st Qu.:4.365   1st Qu.:3.913   1st Qu.: 8  
##  Median : 1.000   Median : 0.000   Median :4.392   Median :4.029   Median :16  
##  Mean   : 3.642   Mean   : 1.438   Mean   :4.331   Mean   :4.000   Mean   :16  
##  3rd Qu.: 8.000   3rd Qu.: 2.000   3rd Qu.:4.416   3rd Qu.:4.134   3rd Qu.:24  
##  Max.   :10.000   Max.   :10.000   Max.   :4.746   Max.   :4.446   Max.   :32  
##                   NA's   :271      NA's   :271     NA's   :271                
##     MortRate        AliveRate          Conso           ConsoBee            Dose         
##  Min.   :0.0000   Min.   :0.0000   Min.   :0.0000   Min.   :0.00000   Min.   :0.000000  
##  1st Qu.:0.0000   1st Qu.:0.2000   1st Qu.:0.0918   1st Qu.:0.01711   1st Qu.:0.000000  
##  Median :0.1111   Median :0.8889   Median :0.2714   Median :0.03533   Median :0.000806  
##  Mean   :0.3647   Mean   :0.6353   Mean   :0.2555   Mean   :0.03264   Mean   :0.266437  
##  3rd Qu.:0.8000   3rd Qu.:1.0000   3rd Qu.:0.3964   3rd Qu.:0.04691   3rd Qu.:0.161545  
##  Max.   :1.0000   Max.   :1.0000   Max.   :0.8466   Max.   :0.15621   Max.   :6.418829  
##                                                                                         
##     DoseBee             CumDose          CumDoseBee         MeanDose       
##  Min.   :0.0000000   Min.   : 0.00000   Min.   :0.00000   Min.   :0.000000  
##  1st Qu.:0.0000000   1st Qu.: 0.00143   1st Qu.:0.00019   1st Qu.:0.000122  
##  Median :0.0000931   Median : 0.25055   Median :0.02709   Median :0.011834  
##  Mean   :0.0409656   Mean   : 6.43732   Mean   :0.88338   Mean   :0.557201  
##  3rd Qu.:0.0236415   3rd Qu.: 9.22429   3rd Qu.:1.00324   3rd Qu.:0.642262  
##  Max.   :1.5758780   Max.   :33.46229   Max.   :6.42030   Max.   :3.625707  
##                                                                             
##   MeanDoseBee       
##  Min.   :0.0000000  
##  1st Qu.:0.0000123  
##  Median :0.0012209  
##  Mean   :0.0738076  
##  3rd Qu.:0.0673039  
##  Max.   :0.5762436  
## 
```

3 Syrup consumption and evaporation

3.1 Evaporation data

We collected partial data about the difference of syrup weight that might be due to simple evaporation by adding syringes with syrup into cages without bees or with 10 dead bees during some days of the experiment (when cages were free because all bees were dead).

One rather extreme data seems to be a transcription or measurement error. If we don't take this outlier into consideration, it appears that the weight loss due to evaporation is between 0.04 and 0.1 g per cage and per day. There is clear variation from day to day so it appears that a daily measurement would be better to correct for evaporation for each day. The presence of dead bees does not seem to affect the values however the presence of living bees might change the evaporation.

We compared the results with and without correction for evaporation (using the average evaporation value after excluding the outlier). The impact of this correction of the daily consumption is discussed in section 3.2. For the rest of the analyses (ea LCx, LDDx, LCDx,... and cumulative toxicity) there were little differences when we applied the correction or not (results not shown here). In the rest of the document we show the results without evaporation consumption because such kind of ecotoxicilogical studies have historically not corrected for evaporation. A more detailed study about the daily evaporation with different number of living be should be performed separately.

Note : As the consumption is generally measured as a consumption per bee, the measurement error caused by evaporation will have a higher impact when the mortality is high (ie when there are few living bees).

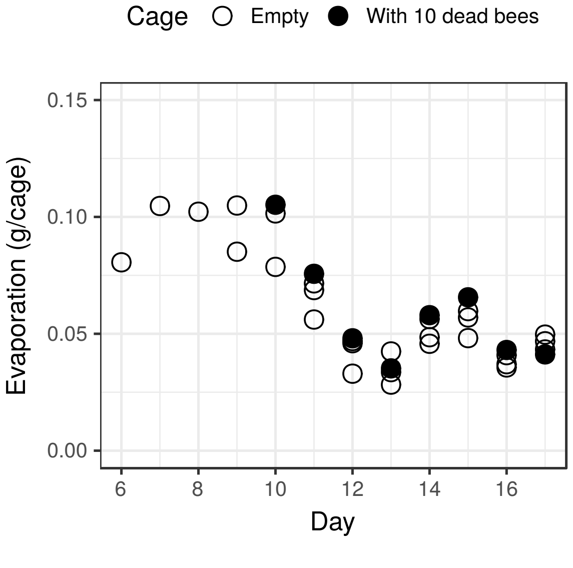

Figure 1:

Graph including the extreme value

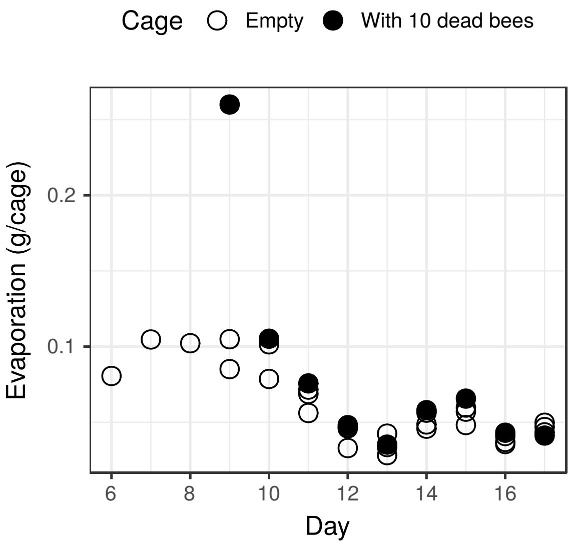
Cage
Empty
With 10 dead bees
Evaporation (g/cage)
0.2
0.1
6
8
10
12
14
16
Day

Figure 2:

3.2 Consumption of syrup between doses and over time

3.2.1 Kinetics of the consumption without evaporation correction

In the following graph, we look at the consumption of syrup (not the dose of a.i.) consumed by the bees (without evaporation correction). Each gray line represent the data of one cage (replicate) for each concentration (each facet of the graph) of boscalid/cantus. The red line is a loess smoother (locally weighted polynomial regression) hat shows the general trend. The data displayed are the mean syrup consumption per bee and per day without evaporation correction.

Main results :

- There is a very large variation of consumption from day to day
- At most concentrations (but not in the control), there is a strong increase of syrup consumption per bee at the end of the experiment, above 0.08 g/bee

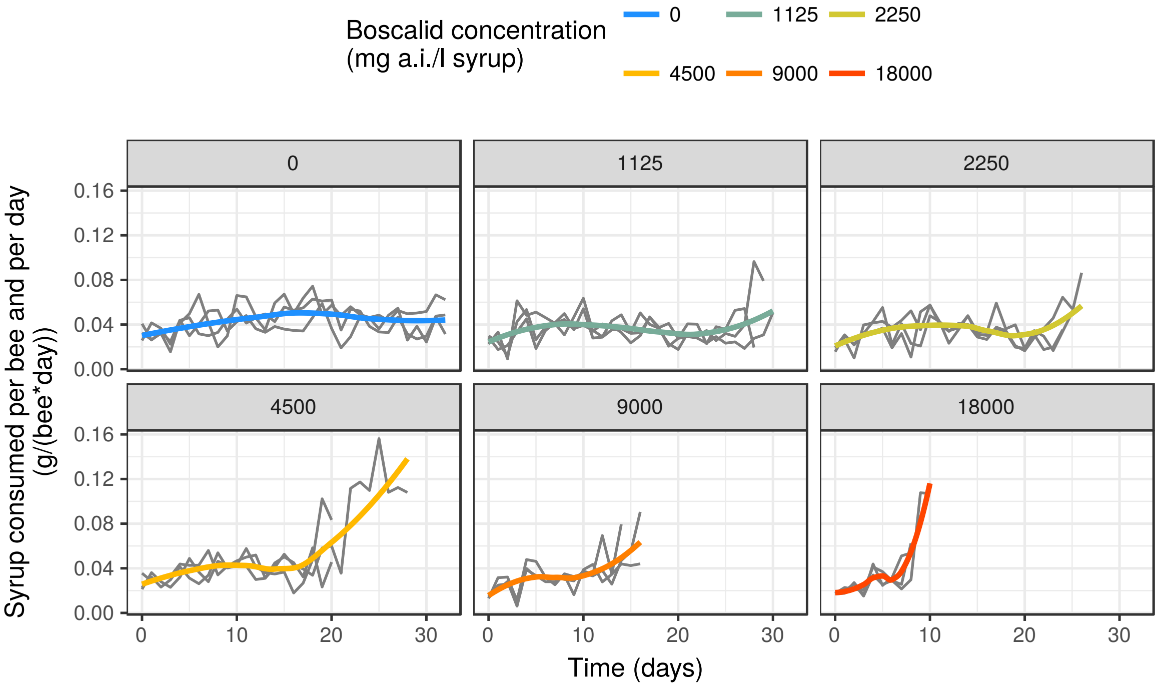

Figure 3:

Here we look at the same response (syrup consumption per bee) but against the number of living bees. The very high levels of syrup consumption (above 0.08 g/bee) occur only when there are only 1 or 2 living bee(s) left (and this case never occurs in the control).
So this increase of consumption at the end of the test could be due to the stress of the only bee left. An important contributing factor could also be the evaporation that has been measured so be between 0.04 and 0.1 g per cage (in empty cages - see section 3.2). When there are 10 bees in the cage, the maximum measurement error (over estimation) of syrup consumption **per bee** due to evaporation would then be between 0.004 and 0.01 g per bee but when there are only one or 2 bee(s) left this can have a stronger effect on the measurement.

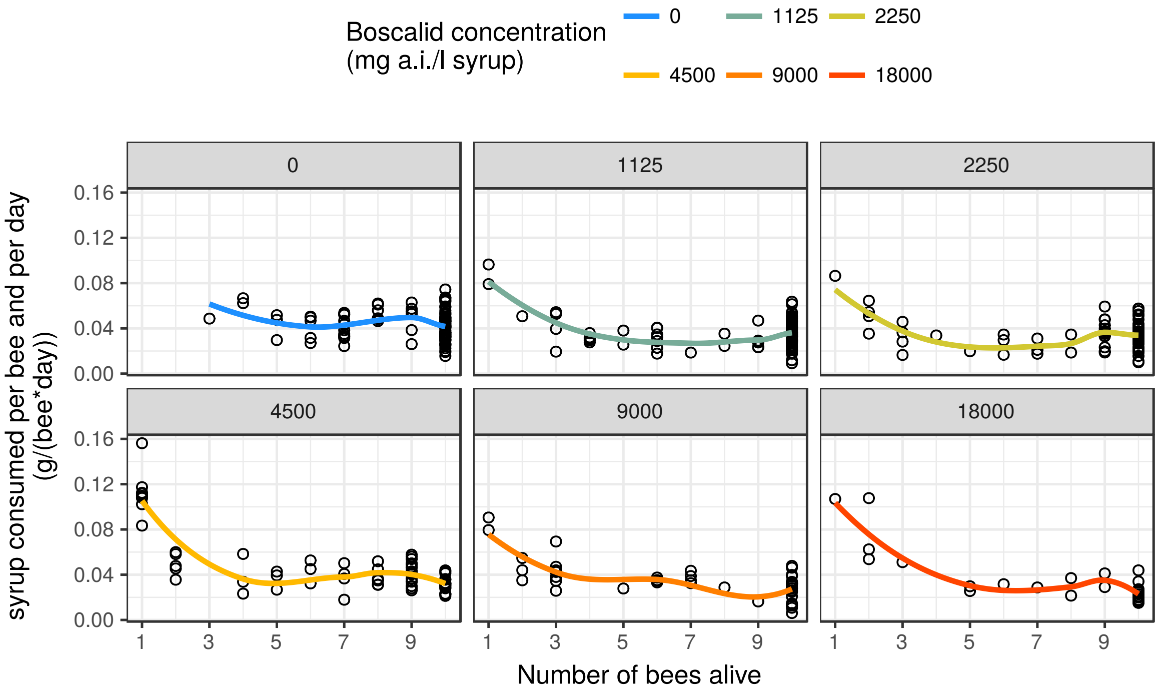

Figure 4:

3.2.2 Kinetics of the consumption with evaporation correction

If we use the data corrected for evaporation, most of the peak consumption at the end of the test disappear. However there are still a few cases of peaking consumption at the doses 4500 mg/l and 18000 mg/l associated with low number of bees. So it seems that these observed peaks might be due to a combination of measurement error due to evaporation (and more important when there are few bees) and the mere fact that the bees are in low number.

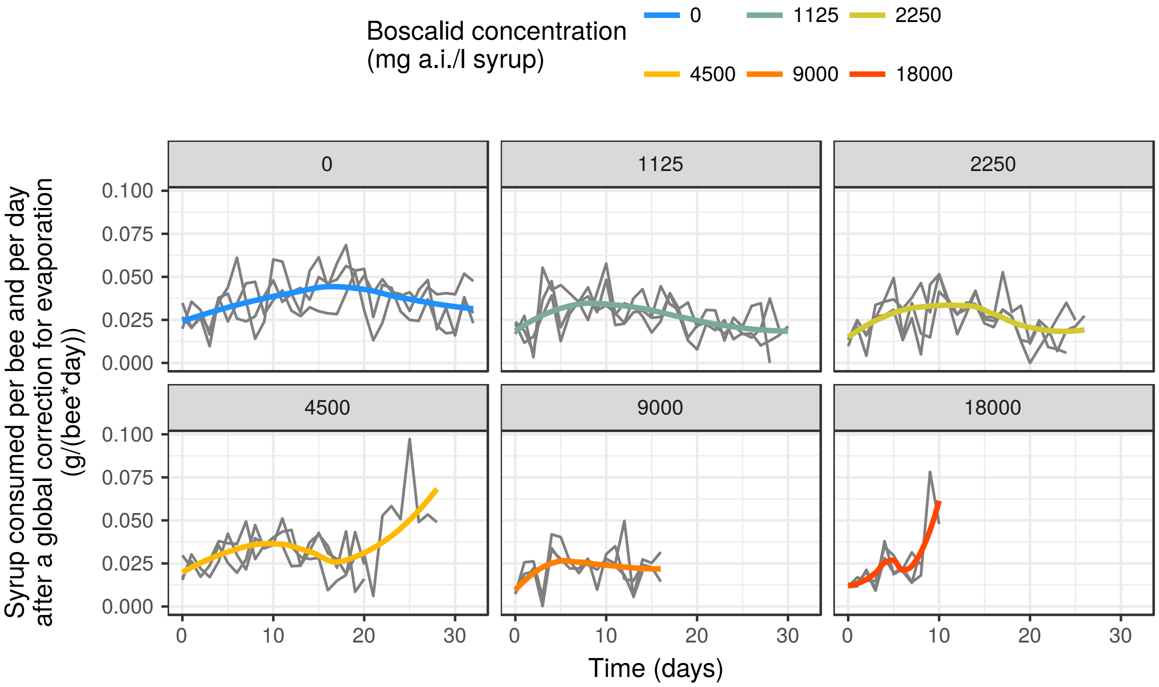

Figure 5:

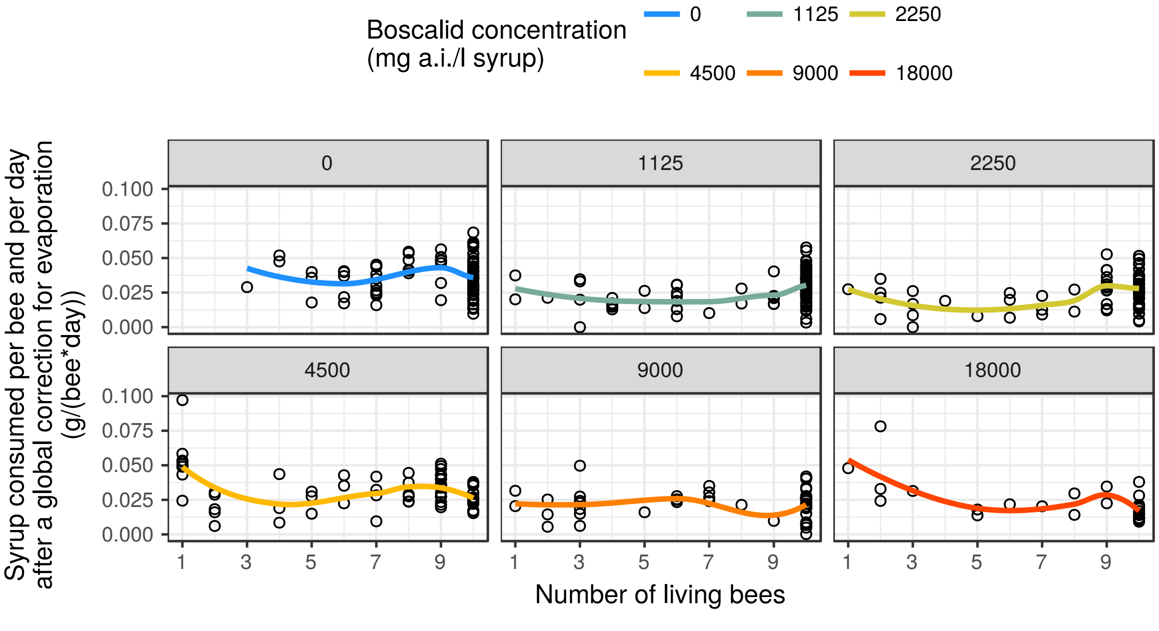

Figure 6:

3.2.3 Kinetics of the consumption up to 50% of mortality

The question of the difference of consumption over time and between doses is mainly important up to 50% mortality (eg for application of the cumulative toxicity test proposed by EFSA, for ED50 calculations . . .). We can then look at the consumption data after removing the data corresponding to more than 50% of mortality. NB : the results hereafter are almost identical with or without correction for evaporation. We show the data without evaporation correction.

There is here a clear pattern of increased consumption at the beginning of the test followed by a decrease. The colored lines are loess smoothers (locally weighted polynomial regression).

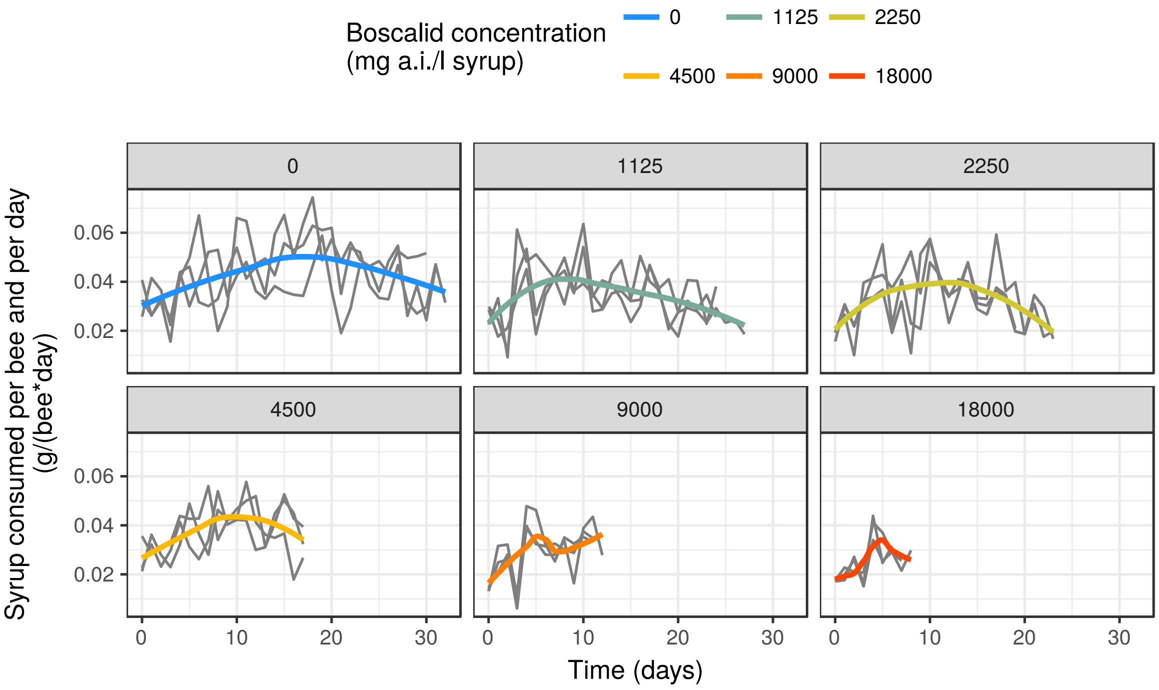

Figure 7:

We can group the loess curves on the same graph. The kinetics of the consumption is clearly different with a peak at different moments and with a trend toward a higher consumption at lower doses

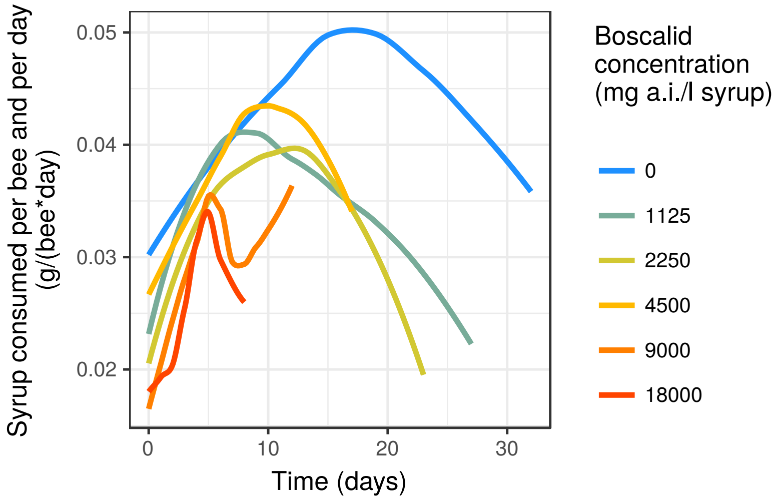

Figure 8:

This visual observation is confirmed by statistical testing.
We used a gaussian mixed model with the consumption per bee per day as response and as fixed explanatory variable the Concentration, a second order polynomial of the time (to take into account the bell shaped relationship) and their first level interactions. The cage was used as random effect. The Time was centered before the analysis (mean day = 12.5) to limit problems of correlations between coefficients. We used only the consumption data when there were at least 5 living bees in the cage (i.e. 50% mortality).

```
library(lme4)
tmp2 <- tmp
tmp2$Time <- scale(tmp2$Time, scale = FALSE)
m <- lmer(ConsoBee ~ Conc + Time + I(Time^2) + Time:Conc + I(Time^2):Conc + (1 |Rep),
          data = tmp2[tmp2$Alive >=5,])
```

The model quality is correct as revealed by residual plots, eg a second order polynomial is sufficient to model the bell shaped relationship. A model without the second order polynomial shows clear problem of non linearity between the consumption and time (as can be expected based the above plots).

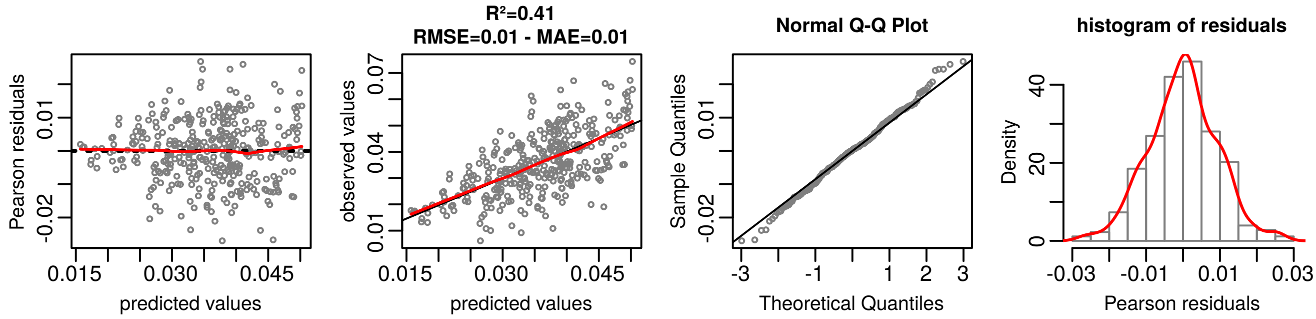

Figure 9:

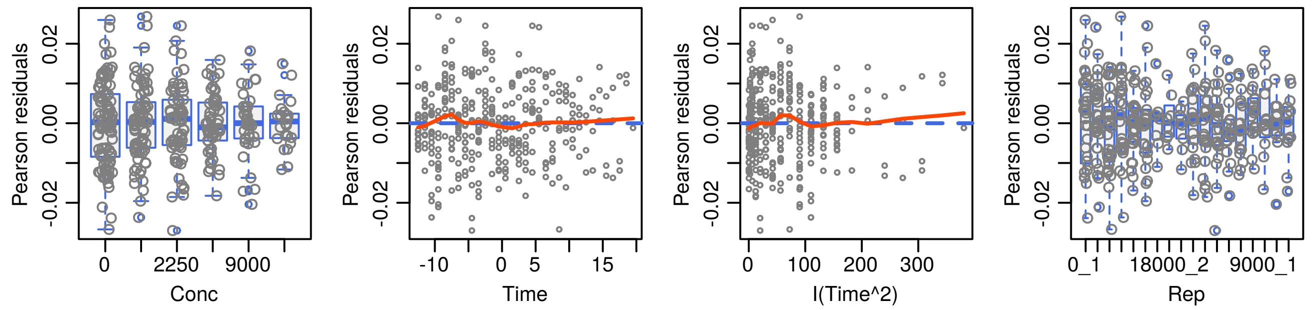

Figure 10:

Likelihood ratio test (Type II)

The time x concentration interactions are significant meaning that the kinetics of the consumption (consumption vs time) is different between the concentrations. The fact that the main Conc (concentration) effect is highly significant means that even without considering these interactions (type II test) the consumption at the average day (~12.5 days) is different between the treatments (concentrations).

NB a model without centering the time still shows clear differences of consumption at day 0 (ie the intercept for non centered data) at least between the control and the 2 highest concentrations.

	LR	df	p(>Chisq)	
**Conc**	30.11	5	1.4e-05	***
**Time**	1.194	1	0.275	
**I(Time^2)**	68.47	1	1.11e-16	***
**Conc:Time**	33.48	5	3.02e-06	***
**Conc:I(Time^2)**	11.87	5	0.0366	*

```
## Linear mixed model fit by REML ['lmerMod']
## Formula: ConsoBee ~ Conc + Time + I(Time^2) + Time:Conc + I(Time^2):Conc +      (1 | Rep)
##    Data: tmp2[tmp2$Alive >= 5, ]
##
## REML criterion at convergence: -2056.4
##
## Scaled residuals:
##      Min       1Q   Median       3Q      Max
## -2.82938 -0.60213  0.02695  0.59812  2.81305
##
## Random effects:
##  Groups   Name        Variance  Std.Dev.
##  Rep      (Intercept) 2.931e-06 0.001712
##  Residual             9.098e-05 0.009539
## Number of obs: 357, groups:  Rep, 18
##
## Fixed effects:
##                       Estimate Std. Error t value
## (Intercept)          4.724e-02  1.720e-03  27.457
## Conc1125            -7.667e-03  2.548e-03  -3.009
## Conc2250            -7.773e-03  2.603e-03  -2.986
## Conc4500            -5.413e-03  2.641e-03  -2.050
## Conc9000            -1.627e-02  6.042e-03  -2.693
## Conc18000           -4.156e-02  2.809e-02  -1.479
## Time                 6.425e-04  1.306e-04   4.921
## I(Time^2)           -6.543e-05  1.267e-05  -5.162
## Conc1125:Time       -8.252e-04  1.937e-04  -4.259
## Conc2250:Time       -1.061e-03  2.316e-04  -4.581
## Conc4500:Time       -1.427e-03  5.163e-04  -2.764
## Conc9000:Time       -2.182e-03  1.923e-03  -1.135
## Conc18000:Time      -7.400e-03  6.622e-03  -1.117
## Conc1125:I(Time^2)  -2.189e-05  2.420e-05  -0.905
## Conc2250:I(Time^2)  -8.110e-05  3.087e-05  -2.627
## Conc4500:I(Time^2)  -1.053e-04  5.563e-05  -1.892
## Conc9000:I(Time^2)  -1.431e-04  1.381e-04  -1.036
## Conc18000:I(Time^2) -4.092e-04  3.718e-04  -1.101
```

However the day to day variability is quite high as can be seen here when we compare the consumption just during the first 4 days of the experiment.

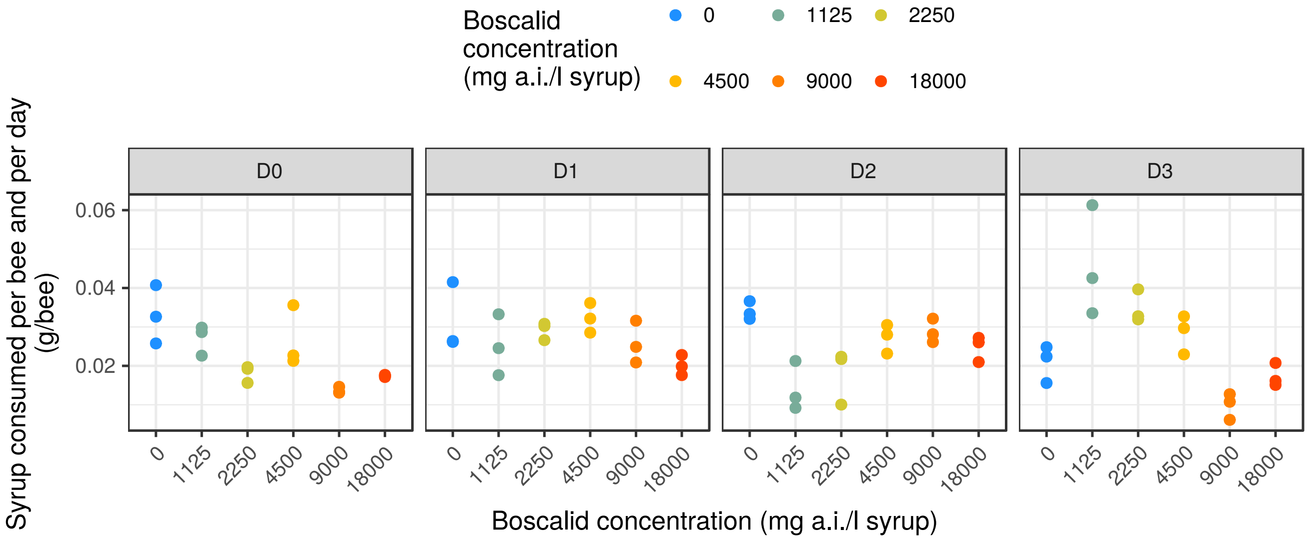

Figure 11:

3.2.4 Average consumption (without taking the time into account)

In most bee tests we compare simply the average consumption between the treatment (without taking into account the time / kinetics).

NB on the following graph the black dots represent the mean and the bars their standard deviation.

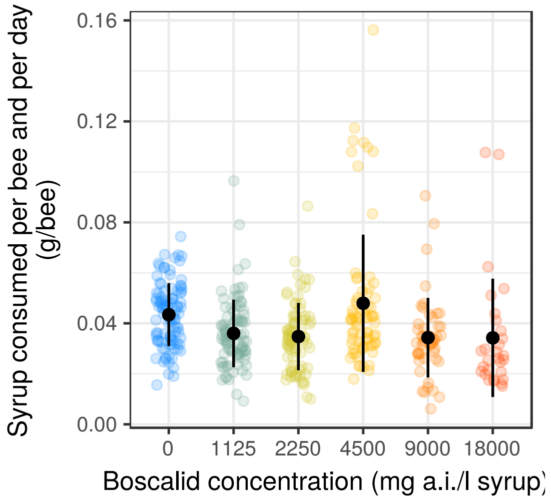

Figure 12:

We can test if there is a significant difference between the treatments with a gaussian mixed model with the concentration as fixed effect and the cage as random effect.
The overall difference test is not significant :

Table 2: Analysis of Deviance Table (Type II Wald F tests with Kenward-Roger df)

	F	Df	Df.res	Pr(>F)
**Conc**	2.289	5	12.2	0.1104

The absence of significant difference might be due to the somewhat extreme consumption values observed when the mortality is high.

Here we use only the consumption data up to 10 days as in a classical chronic test. There is still no difference (NB choosing other time point does not change the results). This is due to the high variability including the variability due to time that is not taken into account here.

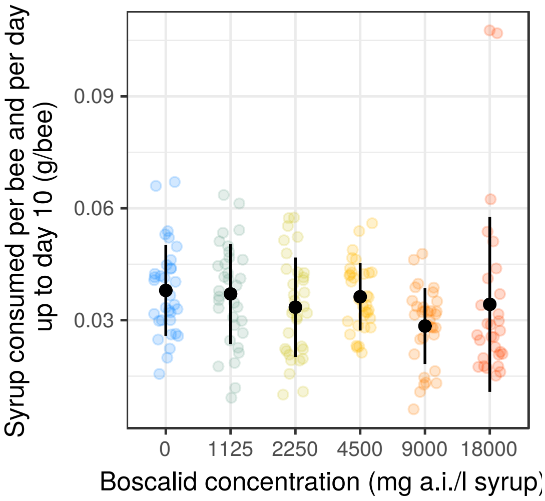

Figure 13:

Table 3: Analysis of Deviance Table (Type II Wald F tests with Kenward-Roger df)

	F	Df	Df.res	Pr(>F)
**Conc**	1.974	5	11.98	0.1553

If you keep only the data up to 50% of mortality, then you can see a significant difference between the concentrations. A post-hoc all pairwise comparison test (`multcomp` package - single step method for p-values adjustment) shows that the consumption is lower in all doses relative to the control excepted at 4500 mg/l. The significant differences are displayed on the graph with letters (concentrations sharing the same letter are not significantly different).

Table 4: Analysis of Deviance Table (Type II Wald F tests with Kenward-Roger df)

	F	Df	Df.res	Pr(>F)
**Conc**	10.65	5	12.8	0.0003313

```
##
##   Simultaneous Tests for General Linear Hypotheses
##
## Multiple Comparisons of Means: Tukey Contrasts
##
##
## Fit: lmer(formula = ConsoBee ~ Conc + (1 | Rep), data = tmp[tmp$Alive >=
##     5, ])
##
## Linear Hypotheses:
##                      Estimate Std. Error z value Pr(>|z|)
## 1125 - 0 == 0       -0.008406   0.002226  -3.776  0.00217 **
## 2250 - 0 == 0       -0.009707   0.002276  -4.266  < 0.001 ***
## 4500 - 0 == 0       -0.005339   0.002370  -2.252  0.21099
## 9000 - 0 == 0       -0.013858   0.002572  -5.388  < 0.001 ***
## 18000 - 0 == 0      -0.017503   0.002887  -6.064  < 0.001 ***
## 2250 - 1125 == 0  -0.001301   0.002337  -0.557  0.99354
## 4500 - 1125 == 0   0.003067   0.002429   1.262  0.80255
## 9000 - 1125 == 0  -0.005452   0.002626  -2.076  0.29574
## 18000 - 1125 == 0 -0.009097   0.002935  -3.100  0.02328 *
## 4500 - 2250 == 0   0.004368   0.002475   1.765  0.48395
## 9000 - 2250 == 0  -0.004150   0.002668  -1.555  0.62388
## 18000 - 2250 == 0 -0.007796   0.002973  -2.622  0.08991 .
## 9000 - 4500 == 0  -0.008519   0.002750  -3.098  0.02339 *
## 18000 - 4500 == 0 -0.012164   0.003046  -3.994  < 0.001 ***
## 18000 - 9000 == 0 -0.003646   0.003205  -1.137  0.86363
## ---
## Signif. codes:  0 '***' 0.001 '**' 0.01 '*' 0.05 '.' 0.1 ' ' 1
## (Adjusted p values reported -- single-step method)
```

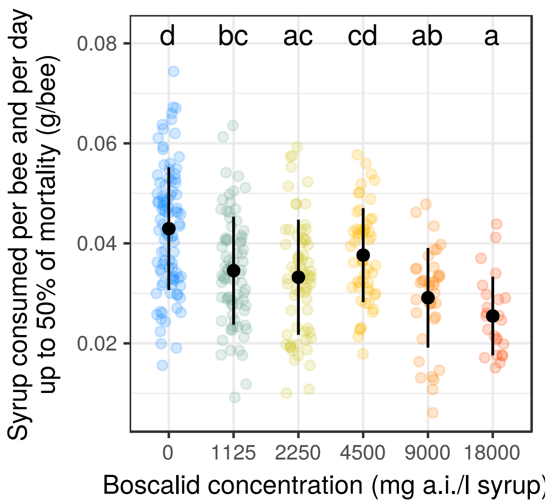

Figure 14:

3.3 Conclusions

Note : the raw consumption data are provided in the file `raw_results/raw_data_with_computed_doses.csv`

Evaporation

- The evaporation is quite variable from day to day and may also be influenced by the presence living bees (here we measured the evaporation in empty cages or cages with dead bees for only a subset of days).
- The effect of evaporation is mainly important in the evaluation of the daily consumption when there are only 1 or 2 living bees left. However the impact of the evaporation (as measured here, ie not very precisely) on the overall results (kinetics of the toxicity and kinetics of the consumption during most of the test) seem very minor.
- Taking into account the evaporation might however improve the estimation of the dose to effect statistics and reduce the day to day measured variability in consumption.

Consumption

- The daily consumption seems to increase strongly when there are only 1 or 2 living bee left. This might be partially due to the measurement error induced by evaporation but even after correcting for evaporation we still observe some large peaks of consumption when there are only a few bees left
- there is a huge day to day variation in consumption even in the control but some general patterns are nevertheless visible.
- Even when there are more than 5 bees per cage, the consumption is not stable over time. In the control, the consumption slowly increases to reach a maximum between 15 and 20 days and then decreases slowly.
- A similar pattern is observed at the different concentrations but with a different kinetics : the higher the concentration the sooner the maximum and the steeper the increase and then decrease.
- The bees tend to have a lower overall consumption in the higher concentrations at a given point in time
- These differences of consumption between treatments are only visible if you take into account the time and/or if you do not use the consumption data after 50% of mortality. Otherwise the variability of the consumption between days masks the differences.

4 Kinetics of the toxicity : LTx, LCx, LDDx, LCDx at different time or concentration

4.1 Raw mortality rates

Mortality rate over time for the different doses of boscalid/cantus, the control and toxic standard. The first vertical dashed line at day 10 shows when the test should have been stopped according to the standard protocol.
The second vertical dashed line shows the time (day 20) where the mortality rate in the control reached 15% i.e. the validity criterion of the 10 day test.
The test was stopped at 33 days when the mortality in the control reached 50%.

The mortality at the highest concentration is higher than 50% only at Day 8. Before that date it is complicated to estimate any LC50 or LDD50. On day 10 the mortality has reached almost 100% at the highest concentration while all the other doses are below 50% and most of hem close to 0%. On Day 20 when the mortality in the control has reached 15%, the mortality in the 3 highest doses is 100% or close to 100%. And on day 31, the mortality of all concentrations has reached 100% while the control is still just below 50%.

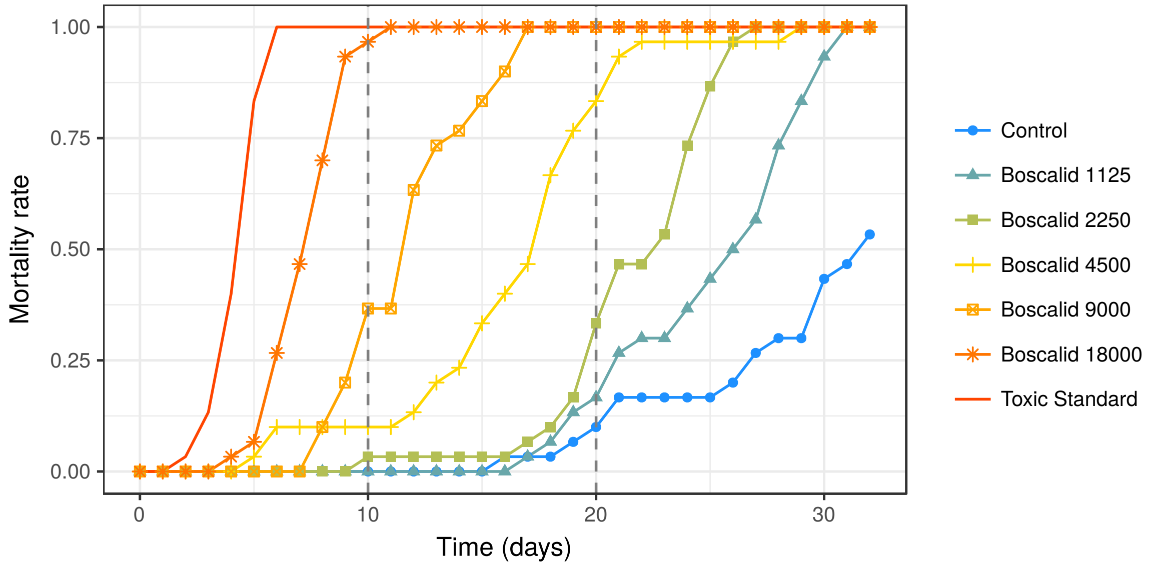

Figure 15:

4.2 LTx vs Concentration

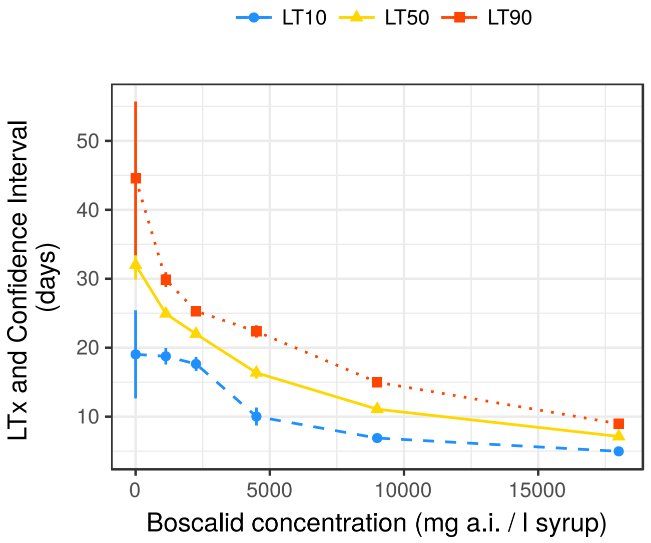

Figure 16:

4.3 LCx vs time

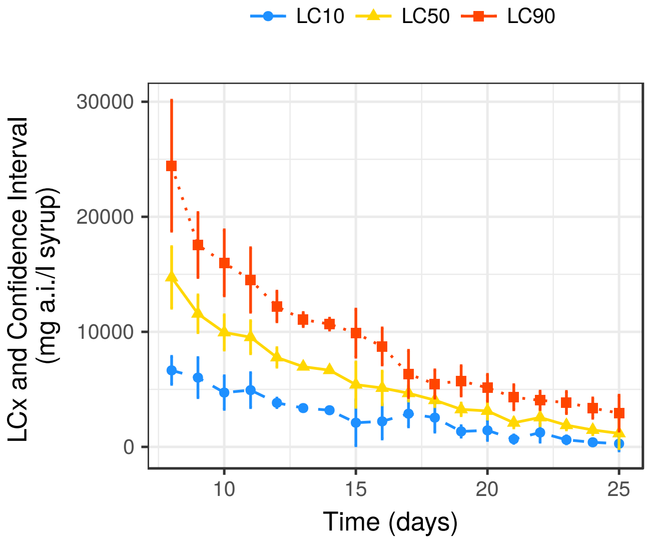

Figure 17:

4.4 LDDx vs Time

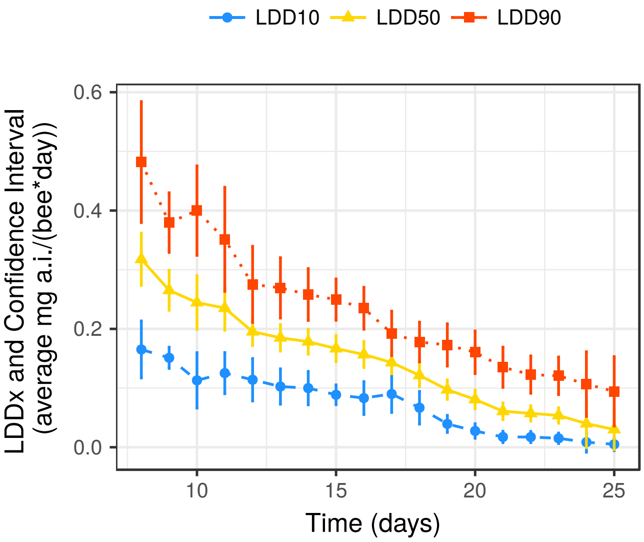

Figure 18:

4.5 LCDx vs Time

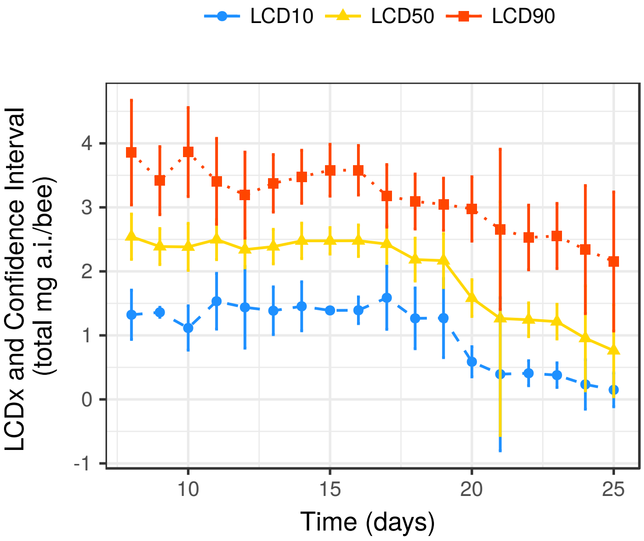

Figure 19:

4.6 Conclusion

The Lethal Times are shorter when the concentration is higher and the Lethal Concentrations and Lethal Daily Doses are decreasing when the exposure time is increasing. This is not very surprising. The question here is to know if this decrease is as expected according to Haber's rule (ie when there is no cumulative toxicity). This question is explored in section 5.1. However it seems to be not very pratical that these standard toxicity estimates depend so much on the exposure time...

The Lethal Cumulated Dose show however a different and interesting pattern : The LCDx is more or less stable up to ~ day 17 and then suddenly starts to decrease. This is a first clue that there might be some cumulative toxicity or change in the capacities of the bees to detoxify the product. The fact that the LCDx is stable during the first part of the tes seems however to be an interesting feature because it means that you can have an estimate of toxicity that is relatively independent of the test duration providing that there is no cumulative toxicity (you need however to maintain the test long enough to reach 50% of mortality in some of the doses).

5 Testing for cumulative toxicity (= "time reinforced toxicity")

We have explored three main ways to check for cumulative toxicity properties of boscalid as described in the literature :

1. Log-Log relationships between concentration and time. If there is no cumulative toxicity, the slope of this linear relationship is expected to be = -1. There are 3 main possibilities :
   - 1a log(Concentration) vs log(LTx)
   - 1b log(LCx) vs log(Time)
   - 1c log(LDx) vs log(Time)
2. Comparing cumulative doses between concentrations (EFSA protocol - EFSA 2013). If there is no cumulative toxicity, you expect to observe the same level of cumulative dose to reach a given level of mortality whatever the concentration.
3. Estimation of α and β : two exponents describing the kinetics between the concentration and the time (method proposed by Miller 2000). Their ratio is expected to be = 1 when there is no cumulative toxicity

5.1 Log-log relationship between concentration and time

According to Haber's rule, if there is no cumulative toxicity, when the concentration/dose is divided by 2 the time of exposure to reach the same level of mortality should be doubled.

There are two main traditional ways to see if the concentration ~ time relationship follows Haber's rule :

1. fit a model of log(Concentration) vs log(LTx)
2. fit a model of log(LCx) vs log(Time) or log(LDDx) vs log(Time)

If the toxicity follows Haber's rule, the slope of these models should be ~= -1. If there is cumulative toxicity, the slope should be lower than -1.

NB : strictly speaking it would be more correct to fit a model of log(LTx) vs log(Concentration) because the LTx is the value estimated with a certain error and this error should be on the y axis. However then the interpretation of the coefficient is reversed : the slope should be higher than -1 when there is cumulative toxicity. This would make the comparison and interpretation less straightforward and this is why we keep the time and LT as X axis for all approaches.

We also estimated a log(LCDx) vs log(Time) model for comparison however this should not be used in the classical way to test if the Haber's rule holds because LCD already includes time accumulation (it is a cumulative dose).
With this model, you expect a slope = 0 under Haber's rule. On the contrary, if there is cumulative toxicity, you expect a slope significantly lower than 0 (and not -1).

We have estimated the slope for each type of model and each level of expected mortality (by steps of 10%). The following graph shows the slope estimates (black dots) and their confidence interval. The vertical dashed line shows the value expected under Haber's rule. (-1).
The confidence intervals are larger (lower precision) for the log(Concentration) vs log(LTx) models because they are based on a lower number of points : 5 points (one for each dose). The control dose has been excluded. The slope is always significantly different from -1 excepted for LT90 (confidence interval : -3.378 -0.995) however the upper bound is very close to -1 and the CI is the largest of all estimates.

As expected the slopes of the log(LCDx) vs log(Time) models are not significantly less than -1 but they are significantly lower than 0.

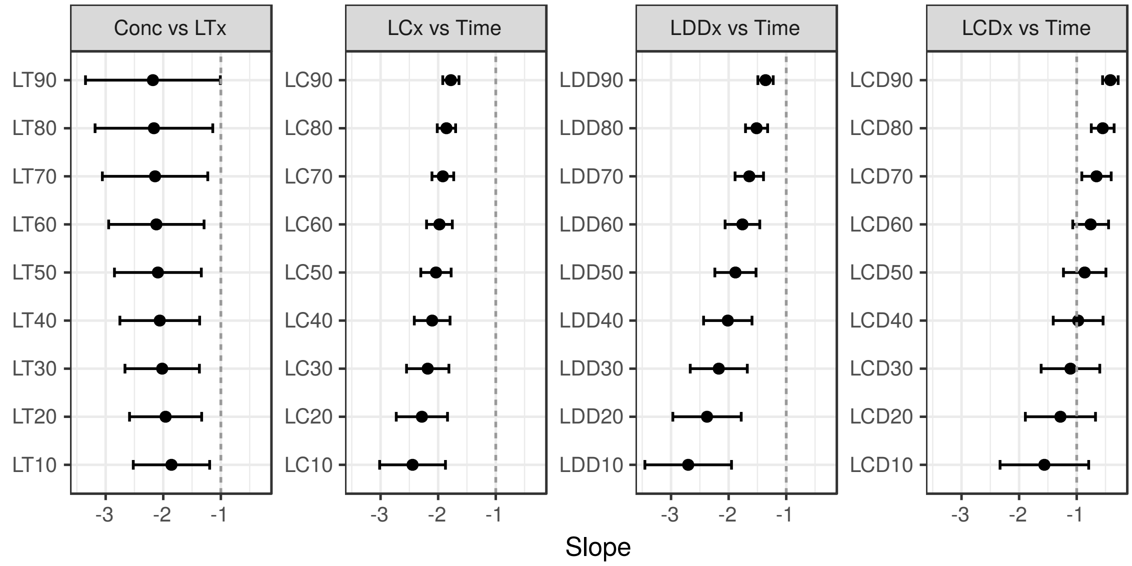

Figure 20:

In conclusion : there is little doubt based on these results that the concentration vs time relationship does not follow Haber's rule and this implies that there is some level of cumulative toxicity.

However, even if all the slopes are significantly smaller than -1, these slopes are just a rough summary of the results and again an interesting pattern appears when we plot for example the log(LDDx) vs log (Time) and compare to the line expected under Haber's rule. NB : the Haber's rule line is a linear regression with a fixed -1 slope (the model estimates only the intercept).

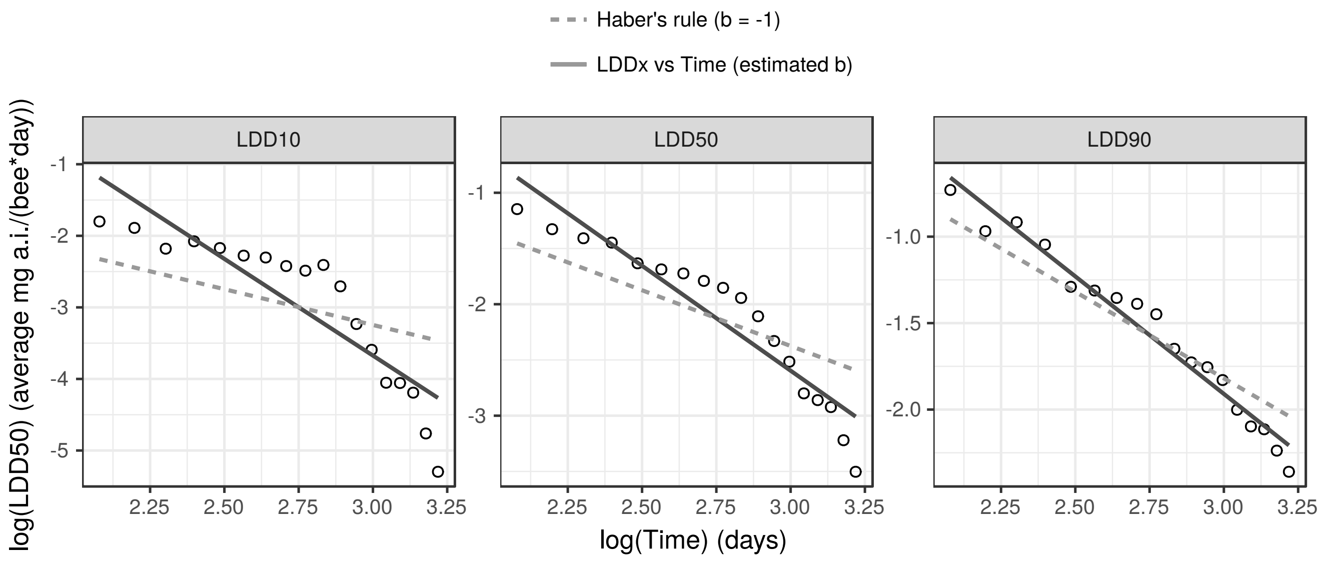

Figure 21:

The log-log LDDx vs Time relationship seems to follow closely Haber's rule up to day 17-18 with a slope of ~ -1 (i.e. when the Dietary Dose is divided by 2, the time to reach the same mortality is doubled). At day 17-18 the slope abruptly decreases and the relationship clearly deviates from Haber's rule. This pattern is more marked for lower levels of mortality (LDD10, LDD20, etc) and tend to disappear at higher levels of mortality (LDD90). For the LDD90 the points are almost perfectly aligned on a straight line without inflection point. However even for the LDD90 where the observed regression line seems to be quite close to the theoretic Haber's slope, the slope is significantly lower than the expected -1 (estimate = -1.359 with a 95% confidence interval of [-1.492,-1.226]).

This pattern is not visible on the LTx vs Concentration Log-Log graphs and is less marked on the LCx vs Time graphs. However the LTx vs Concenration regression are based on only 5 points (one for each concentration) and it might be therefore difficult to visualise the sudden change visible on the LDDx plot.

All the graphs are provided below

5.1.1 log(Concentraton) vs log(LTx)

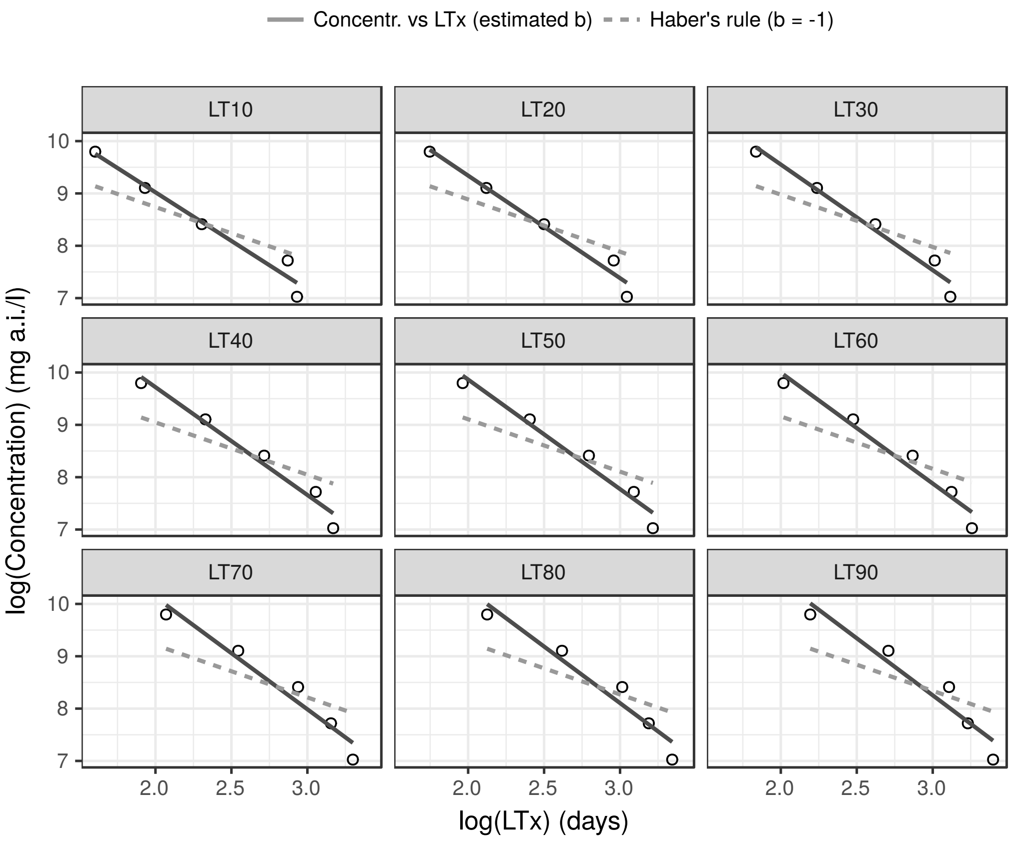

Figure 22:

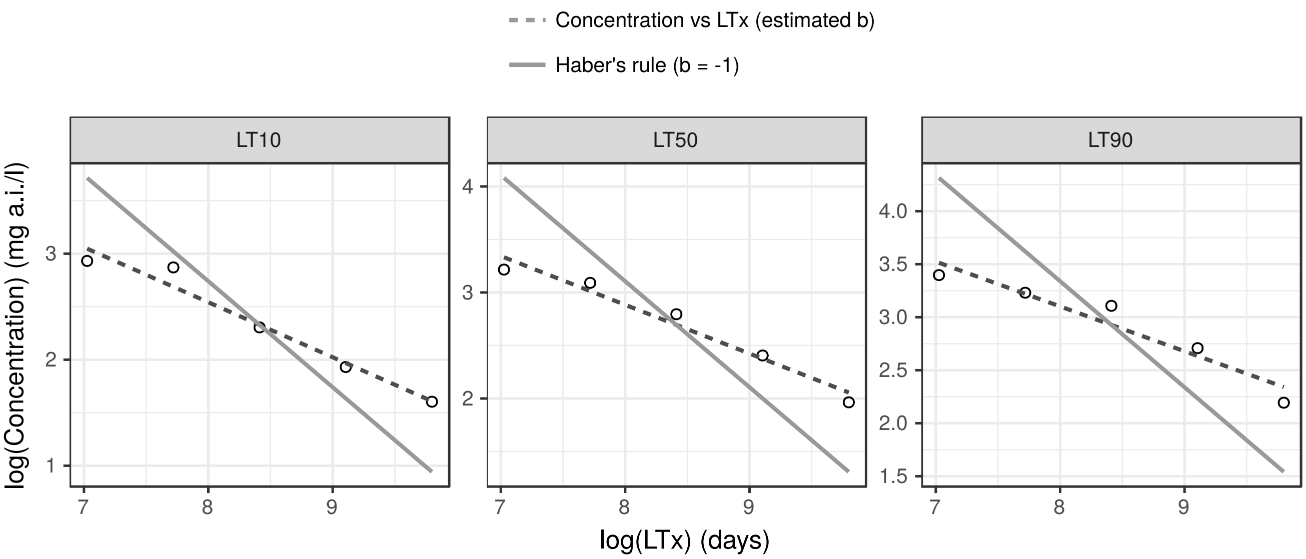

Figure 23:

5.1.2 log(LCx) vs log(Time)

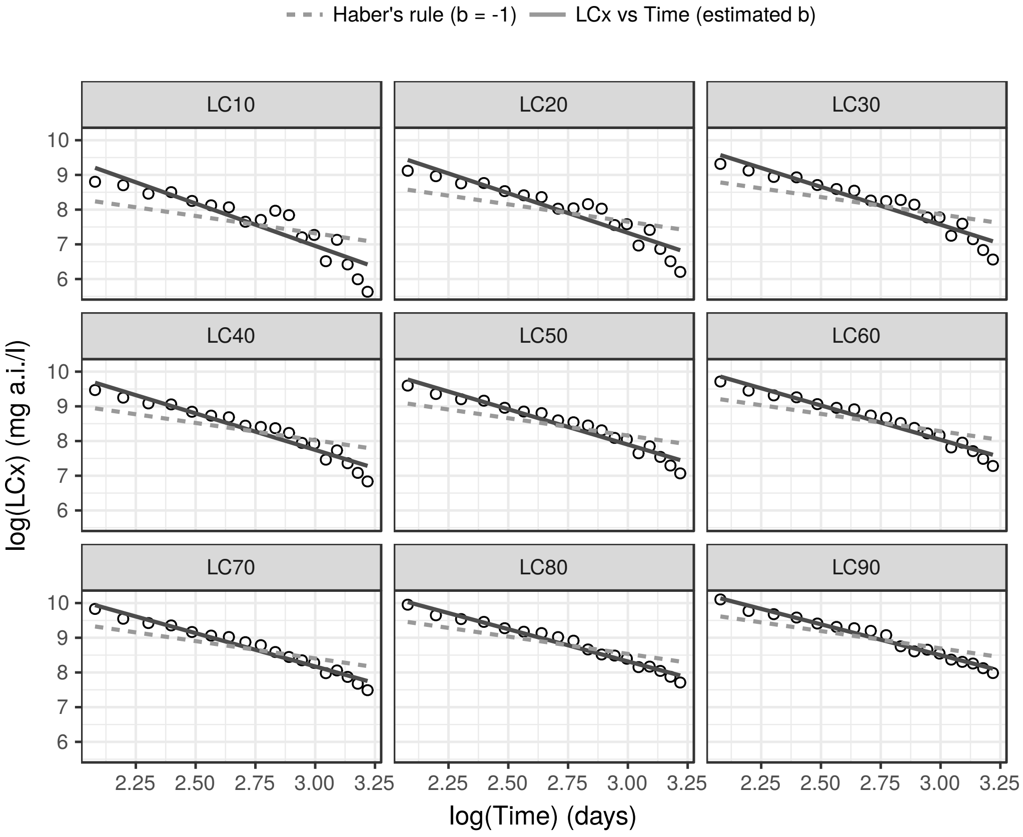

Figure 24:

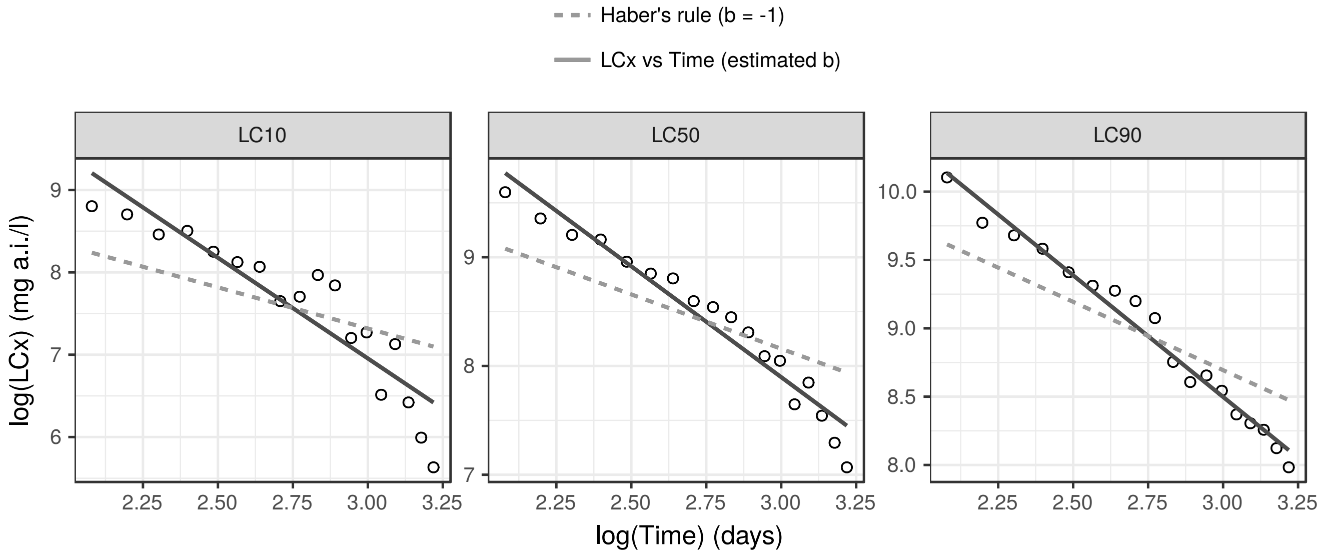

Figure 25:

5.1.3 log(LDDx) vs log(Time)

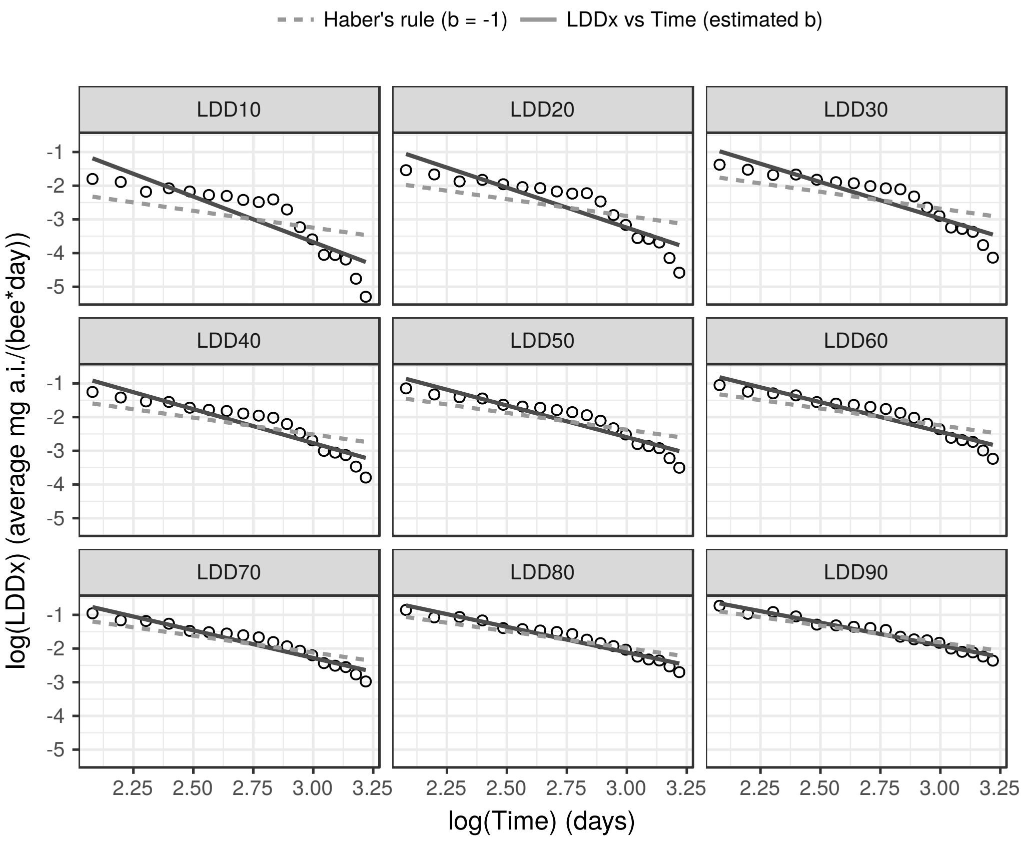

Figure 26:

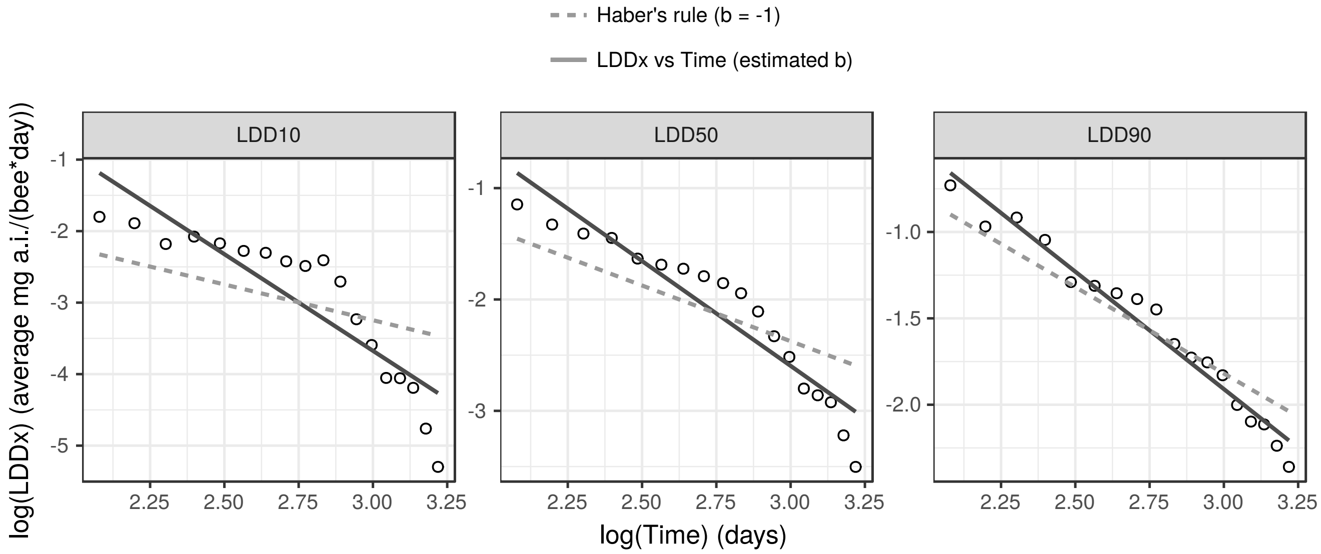

Figure 27:

5.1.4 log(LCDx) vs log(Time)

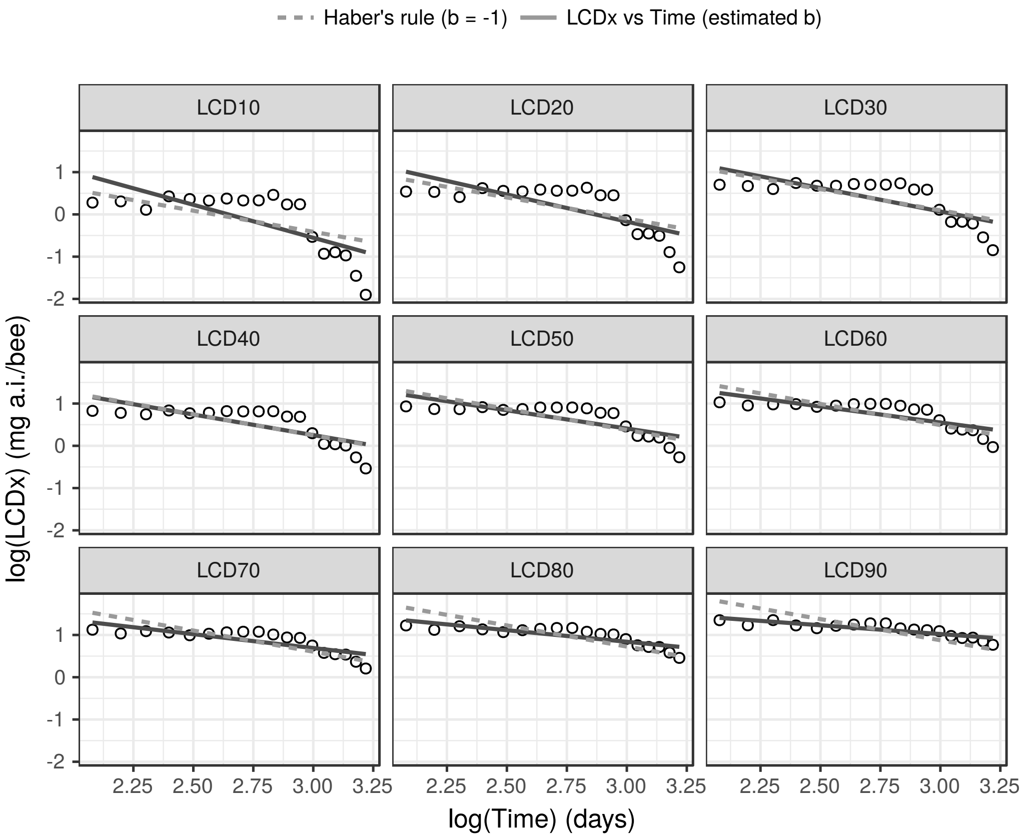

Figure 28:

Only with LCD10, LCD50 and LCD90 with free y scales

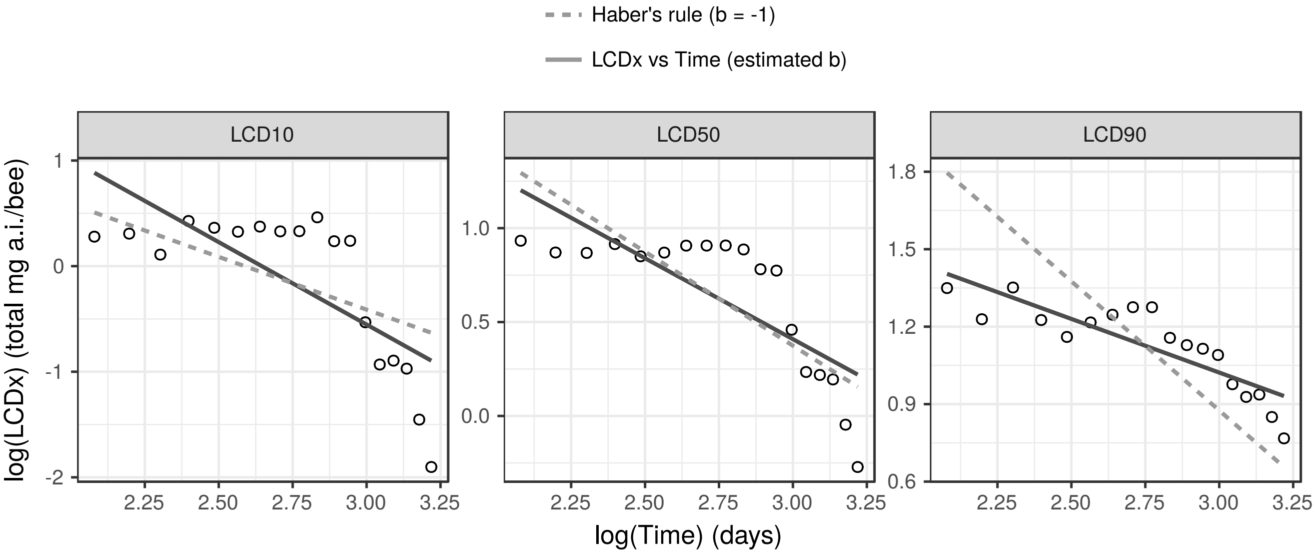

Figure 29:

Raw results of the model (slope and confidence intervals) :

Method	EDx	Slope	CIlower	CIupper
Conc vs LTx	LT10	-1.858	-2.521	-1.194
Conc vs LTx	LT20	-1.959	-2.586	-1.333
Conc vs LTx	LT30	-2.019	-2.665	-1.373
Conc vs LTx	LT40	-2.061	-2.753	-1.368
Conc vs LTx	LT50	-2.093	-2.847	-1.339
Conc vs LTx	LT60	-2.12	-2.948	-1.292
Conc vs LTx	LT70	-2.142	-3.057	-1.227
Conc vs LTx	LT80	-2.162	-3.184	-1.14
Conc vs LTx	LT90	-2.18	-3.35	-1.009
LCx vs Time	LC10	-2.445	-3.016	-1.873
LCx vs Time	LC20	-2.283	-2.728	-1.838
LCx vs Time	LC30	-2.182	-2.549	-1.814
LCx vs Time	LC40	-2.104	-2.414	-1.794
LCx vs Time	LC50	-2.038	-2.302	-1.775
LCx vs Time	LC60	-1.978	-2.202	-1.754
LCx vs Time	LC70	-1.919	-2.108	-1.73
LCx vs Time	LC80	-1.856	-2.016	-1.697
LCx vs Time	LC90	-1.779	-1.92	-1.638
LDDx vs Time	LDD10	-2.702	-3.454	-1.95
LDDx vs Time	LDD20	-2.375	-2.969	-1.782
LDDx vs Time	LDD30	-2.171	-2.667	-1.676
LDDx vs Time	LDD40	-2.015	-2.435	-1.594
LDDx vs Time	LDD50	-1.882	-2.24	-1.524
LDDx vs Time	LDD60	-1.76	-2.061	-1.459
LDDx vs Time	LDD70	-1.642	-1.888	-1.395
LDDx vs Time	LDD80	-1.515	-1.707	-1.323
LDDx vs Time	LDD90	-1.359	-1.492	-1.226
LCDx vs Time	LCD10	-1.561	-2.331	-0.792
LCDx vs Time	LCD20	-1.283	-1.891	-0.6738
LCDx vs Time	LCD30	-1.108	-1.617	-0.5995
LCDx vs Time	LCD40	-0.9749	-1.408	-0.5421
LCDx vs Time	LCD50	-0.8615	-1.23	-0.4928
LCDx vs Time	LCD60	-0.7578	-1.069	-0.447
LCDx vs Time	LCD70	-0.6564	-0.9116	-0.4012
LCDx vs Time	LCD80	-0.5485	-0.7468	-0.3503
LCDx vs Time	LCD90	-0.4155	-0.5507	-0.2803

5.2 Comparing cumulative dose between concentrations (EFSA protocol)

To test for cumulative toxicity the EFSA (2013) propose to :

- determine the LC50 at 48h
- launch a test with bees fed at a high dose = LC50 and others fed at a low dose = 0.25*LC50
- measure the total active substance consumed when 50% mortality occured for both the high dose and the low dose
- compare these values with a t test (with a power of 80% to detect a difference of 35%)
- You can express the difference as a % of the high dose consumption : 100* (high dose consumption - low dose consumption)/high dose consumption
- if there is cumulative toxicity, you expect that the low dose total consumption to reach 50% of mortality will be lower than the total consumption of the higher dose

Here the LC50 at 48h is impossible to estimate. However we can apply the same idea by comparing the cumulated dose consumed when the mortality reaches 50%.

We compare between the concentration the cumulative dose consumed per bee once the cage reached 50% mortality. We use a simple analysis of variance folowed by all pairwise post-hoc comparisons (similar to a Tukey test here using `multcomp` package single-step p-value correction method). Here the LC50 at 10 days is ~ 10000 mg ai/l (close to our 9000 dose). However if Haber's rule holds and that you want to test a quarter of this dose you expect to have to wait ~ 40 days to reach the same mortality…

If there is no cumulative toxicity, no differences in total doses between the treatments (concentrations) are expected.

5.2.1 With raw mortalities

The global differences are highly significant. If you compare the 3 highest concentrations they are all significantly different from concentrations four times lower. NB : a student test would also be significant as the student t test is more powerfull than this corrected p-value test. So applying the EFSA protocol could have worked here but the toxicity was undetectable at 2 days.

NB : these are non corrected mortalities. This could impact the results because the honey bees will finally die even with very low doses of the product.

Another potential problem is that some times (quite often in fact) you do not observe an exact mortality of 50%. For example you have 4 dead bees one day and then you have 6 or 7 dead bees the next day. You could expect that the cumulated dose from the cages with more than 5 dead bees might be higher but it does not seem to be the case here (see on the graph below)

Anova table :

Table 6: Analysis of Variance Table

	Df	Sum Sq	Mean Sq	F value	Pr(>F)
**Conc_f**	4	8.304	2.076	212	1.279e-09
**Residuals**	10	0.09795	0.009795	NA	NA

Graph : concentrations with different letters are significantly different (post-hoc Tukkey like test). Some horizontal noise has been added to the points position to avoid overplotting.

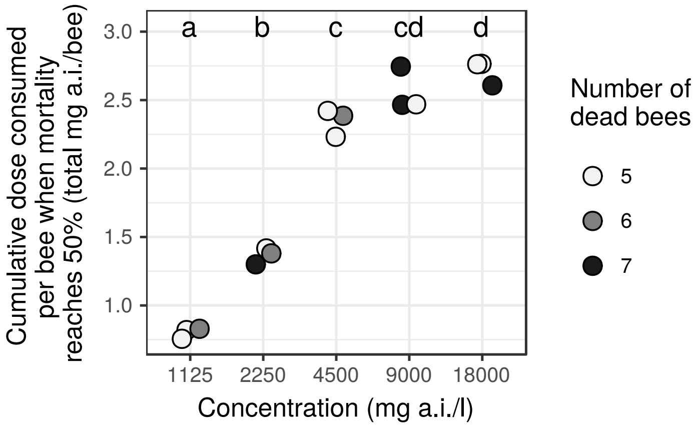

Figure 30:

Raw data for the record :

Treat	Rep	Conc	Eff	Day	Alive	CumDoseBee
CONTROL	1	0	10	D30	5	0
CONTROL	2	0	10	D30	5	0
CANTUS	1	1125	10	D24	5	0.8199
CANTUS	2	1125	10	D28	4	0.8296
CANTUS	3	1125	10	D24	5	0.7563
CANTUS	1	2250	10	D20	3	1.3
CANTUS	2	2250	10	D23	5	1.416
CANTUS	3	2250	10	D24	4	1.38
CANTUS	1	4500	10	D17	5	2.232
CANTUS	2	4500	10	D18	4	2.385
CANTUS	3	4500	10	D16	5	2.421
CANTUS	1	9000	10	D12	3	2.745
CANTUS	2	9000	10	D12	3	2.465
CANTUS	3	9000	10	D12	5	2.469
CANTUS	1	18000	10	D7	3	2.608
CANTUS	2	18000	10	D7	5	2.765
CANTUS	3	18000	10	D8	5	2.762

5.2.2 With corrected mortalities

Applying a mortality correction adequately is not straightforward.
Ideally we should group the data from the 3 replicates and have identical initial number of bees. However as here we use only the mortality rate to select when we should stop looking at cumulated consumption, one of the forms of Abbot's correction seems appropriate : `CorMortRate = (MortRate - MortRateControl)/(1-MortRateControl)` where `MortRate` is the mortalitay rate on a given day in a given replicate and `MortRateControl` is the global mortality rate of the 3 control replicates pooled on the same day.

The results are quite similar excepted that the difference of total consumption between concentration 18000 and 4500 is no more significant.
Note however that this difference would be borderline significant with a single student t test (without correction of the p-values - as recommended by the EFSA protocol) with p-value = 0.057.

Anova table :

Table 8: Analysis of Variance Table

	Df	Sum Sq	Mean Sq	F value	Pr(>F)
**Conc_f**	4	8.351	2.088	119.5	2.128e-08
**Residuals**	10	0.1747	0.01747	NA	NA

Graph : concentrations with different letters are significantly different (post-hoc Tukey like test). Some horizontal noise has been added to the points position to avoid overplotting.

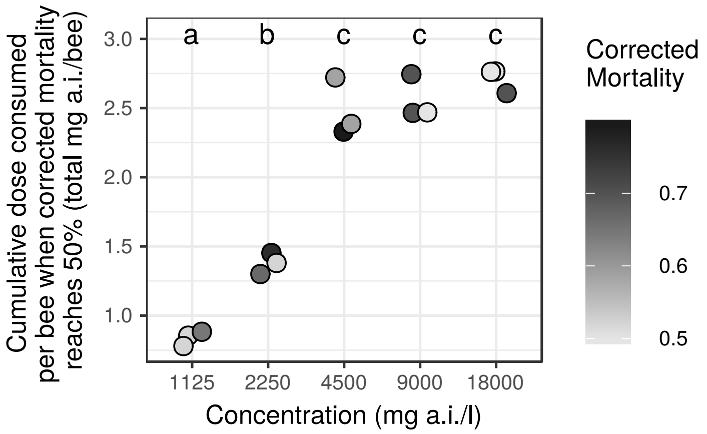

Figure 31:

Raw data for the record :

Treat	Rep	Conc	Day	Eff	Dead	CumDoseBee	MortRateControl	CorMortRate
CANTUS	1	1125	D25	10	6	0.8547	0.1667	0.52
CANTUS	2	1125	D30	10	8	0.883	0.4333	0.6471
CANTUS	3	1125	D25	10	6	0.7798	0.1667	0.52
CANTUS	1	2250	D20	10	7	1.3	0.1	0.6667
CANTUS	2	2250	D24	10	8	1.452	0.1667	0.76
CANTUS	3	2250	D24	10	6	1.38	0.1667	0.52
CANTUS	1	4500	D18	10	8	2.33	0.03333	0.7931
CANTUS	2	4500	D18	10	6	2.385	0.03333	0.5862
CANTUS	3	4500	D18	10	6	2.722	0.03333	0.5862
CANTUS	1	9000	D12	10	7	2.745	0	0.7
CANTUS	2	9000	D12	10	7	2.465	0	0.7
CANTUS	3	9000	D12	10	5	2.469	0	0.5
CANTUS	1	18000	D7	10	7	2.608	0	0.7
CANTUS	2	18000	D7	10	5	2.765	0	0.5
CANTUS	3	18000	D8	10	5	2.762	0	0.5

Mean cumulative dose for each treatment :

Conc	CumDoseBee
1125	0.8392
2250	1.378
4500	2.479
9000	2.56
18000	2.712

Another way to look at these data is to plot the cumulative doses consumed by the bees and visualize when you reach e.g. 50% mortality. The black dots show the LT50 for each concentration. If there were no cumulative toxicity, one would expect that the LT50 will be reached at similar cumulative doses (or at similar time as the control). It is not the case here : we observe that the cumulative dose leading to 50% of mortality at the lowest concentration (1125 mg a.i./ml) is less than half the cumulative dose killing 50% of the bees at 4500, 9000 and 18000 (mg a.i/ml).

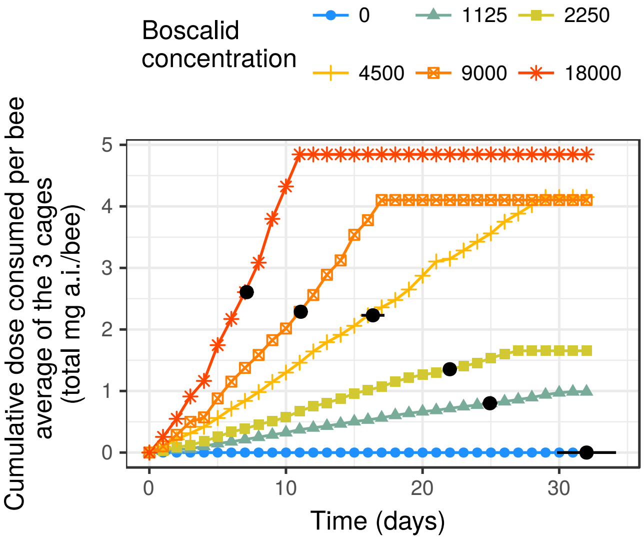
Boscalid concentration
0
1125
2250
4500
9000
18000
Cumulative dose consumed per bee
average of the 3 cages
(total mg a.i./bee)
0
1
2
3
4
5
0
10
20
30
Time (days)

Figure 32:

5.3 Estimation of α and β

The idea here is to use a more general form of the C vs t relationship :

$$C^{\alpha} t^{\beta} = k$$

That relationship can be simplified into

$$C = k^{1/\alpha} t^{-\beta/\alpha} = k' t^{\gamma}$$

where γ is equivalent to the b of the classical equation and should be $= 1$ when Haber's rule holds. According to Miller et al. (2000) we could estimate α and β with a probit model : $Y = m + \alpha log(C) + \beta log(t)$ where Y are the observed mortalities.

We used a binomial GLM with a probit link (estimated by quasilikelihood to take into account overdispersion). Because of the presence of 0 values we used a log(x+1) transformation for both Concentration and Time.

We tested other link functions (logit, cloglog which is equivalent to a weibull, cauchit) but the probit was the one that provided the best fit (lowest QAICc).

```
m <- glm(MortRate ~ log(Conc+1) + log(Time+1), weights = Eff,
         family = quasibinomial(link = "probit"),
         data = d[d$Treat %in% c("CONTROL", "CANTUS"),])

summary(m)
```

```
##
## Call:
## glm(formula = MortRate ~ log(Conc + 1) + log(Time + 1), family = quasibinomial(link = "probit")
##     data = d[d$Treat %in% c("CONTROL", "CANTUS"), ], weights = Eff)
##
## Deviance Residuals:
##     Min       1Q   Median       3Q      Max
## -4.8033  -0.8298  -0.0007   1.2270   5.0995
##
## Coefficients:
##                Estimate Std. Error t value Pr(>|t|)
## (Intercept)   -9.43785    0.37518  -25.16   <2e-16 ***
## log(Conc + 1)  0.32348    0.01737   18.63   <2e-16 ***
## log(Time + 1)  2.45941    0.10535   23.34   <2e-16 ***
## ---
## Signif. codes:  0 '***' 0.001 '**' 0.01 '*' 0.05 '.' 0.1 ' ' 1
##
## (Dispersion parameter for quasibinomial family taken to be 2.760164)
##
##     Null deviance: 6191.8  on 593  degrees of freedom
## Residual deviance: 1745.0  on 591  degrees of freedom
## AIC: NA
##
## Number of Fisher Scoring iterations: 7
```

```r
# confint(m)
# diagplot(m)
# diagplot2(m)
# coef(m)[3] / coef(m)[2] # b = beta / alpha
```

The regression coefficient for the concentration (α) is 0.323 and the regression coefficient for time (β) is 2.459 and $\gamma = b = \beta/\alpha = 7.603$. This would indicate a high level of cumulative toxicity, however this value is very different from the value calculated with the more classical approach above ($\sim$ 1.5 - 2).

However this model does not fit the real data very well. It is quite clear that this model tends to underestimate toxicity at higher concentrations and to overestimate toxicity at low concentrations. The black line should be less steep.

In the graph below the two predictive variables, Concentration and time, are represented on y and x axes and the color represent the predicted values for the response (mortality). The line represent the 50% mortality predicted values. The points (for the 3 replicates) show wen we observed 50% mortality in each cage.

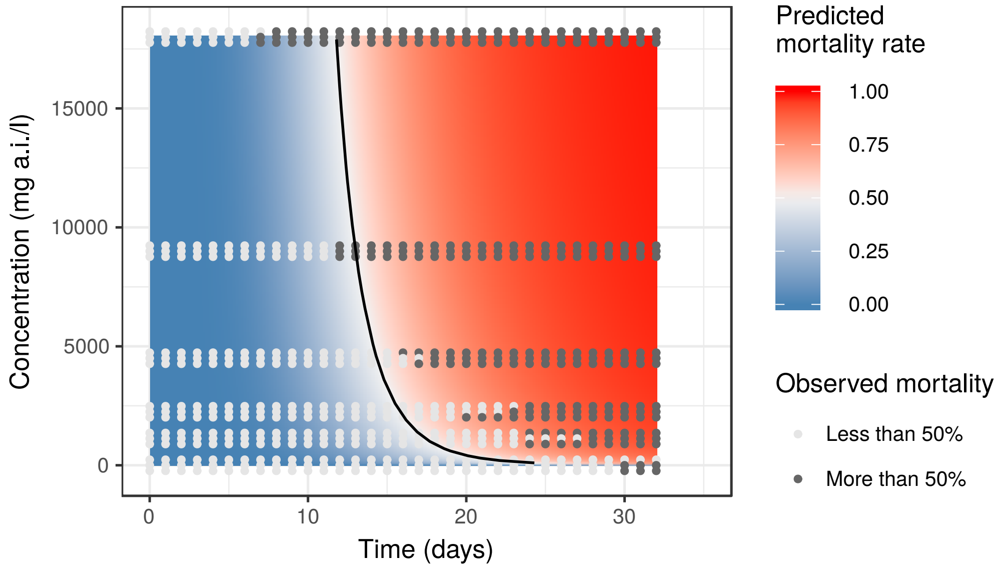

Figure 33:

We can apply the same method on the average daily dose instead of the concentration. The fit is better (lower QAICc than the previous model)

```r
m <- glm(MortRate ~ log(MeanDoseBee+1) + log(Time+1), weights = Eff,
          family = quasibinomial(link = "probit"),
         data = d[d$Treat %in% c("CONTROL", "CANTUS"),])
summary(m)
```

```
##
## Call:
## glm(formula = MortRate ~ log(MeanDoseBee + 1) + log(Time + 1),
##     family = quasibinomial(link = "probit"), data = d[d$Treat %in%
##         c("CONTROL", "CANTUS"), ], weights = Eff)
##
## Deviance Residuals:
##     Min       1Q   Median       3Q      Max
```

```
## -2.9522  -0.2933   0.0000   0.2968   4.2266
##
## Coefficients:
##                      Estimate Std. Error t value Pr(>|t|)
## (Intercept)           -12.797      2.412  -5.306 1.59e-07 ***
## log(MeanDoseBee + 1)   16.690      3.364   4.961 9.19e-07 ***
## log(Time + 1)           3.769      0.724   5.205 2.68e-07 ***
## ---
## Signif. codes:  0 '***' 0.001 '**' 0.01 '*' 0.05 '.' 0.1 ' ' 1
##
## (Dispersion parameter for quasibinomial family taken to be 38.29148)
##
##     Null deviance: 6191.84  on 593  degrees of freedom
## Residual deviance:  564.12  on 591  degrees of freedom
## AIC: NA
##
## Number of Fisher Scoring iterations: 8
```

- Concentration coefficient : $\alpha = 16.69$
- Time coefficient : $\beta = 3.769$
- $\gamma = b = \beta/\alpha = 0.226$

This is now much lower than the b value estimated before but also much lower than b=1 expected under Haber's rule…

Compared to the real data this model seems to do a better job. NB in the following graph the points might be slightly moved vertically to limit overplotting.

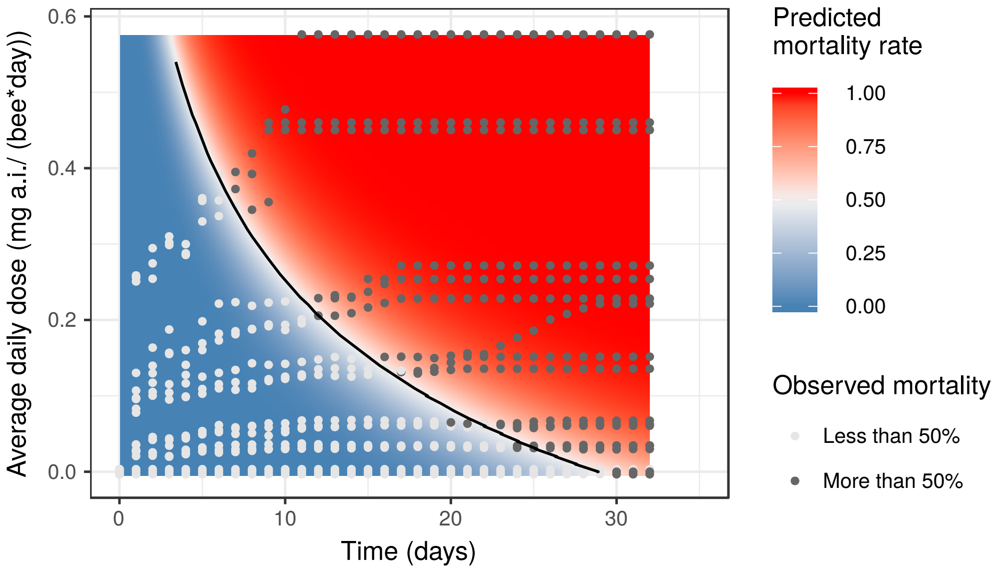

Figure 34:

Interestingly if instead of using the observed mortalities, we use the LCx data with the intended mortality rate x as the response and Time and the LC estimate instead of the concentration, the beta calculated is more similar to what could be expected.

```r
m <- glm(PctMort/100 ~ log(Estimate+1) + log(Time+1),
         family = quasibinomial(link = "probit"), data = LC[LC$Model == "Weibull2",])
summary(m)
```

```
##
## Call:
## glm(formula = PctMort/100 ~ log(Estimate + 1) + log(Time + 1),
##     family = quasibinomial(link = "probit"), data = LC[LC$Model ==
##         "Weibull2", ])
##
## Deviance Residuals:
##      Min        1Q    Median        3Q       Max
## -0.60002  -0.17560   0.01587   0.18940   0.67590
##
## Coefficients:
##                    Estimate Std. Error t value Pr(>|t|)
## (Intercept)       -21.67090    1.14794  -18.88   <2e-16 ***
## log(Estimate + 1)   1.49501    0.07808   19.15   <2e-16 ***
## log(Time + 1)       3.25344    0.18921   17.19   <2e-16 ***
## ---
## Signif. codes:  0 '***' 0.001 '**' 0.01 '*' 0.05 '.' 0.1 ' ' 1
##
## (Dispersion parameter for quasibinomial family taken to be 0.07961514)
##
##     Null deviance: 47.752  on 161  degrees of freedom
## Residual deviance: 10.337  on 159  degrees of freedom
## AIC: NA
##
## Number of Fisher Scoring iterations: 4
```

- Concentration coefficient : $\alpha = 1.495$
- Time coefficient : $\beta = 3.253$
- $\gamma = b = \beta/\alpha = 2.176$

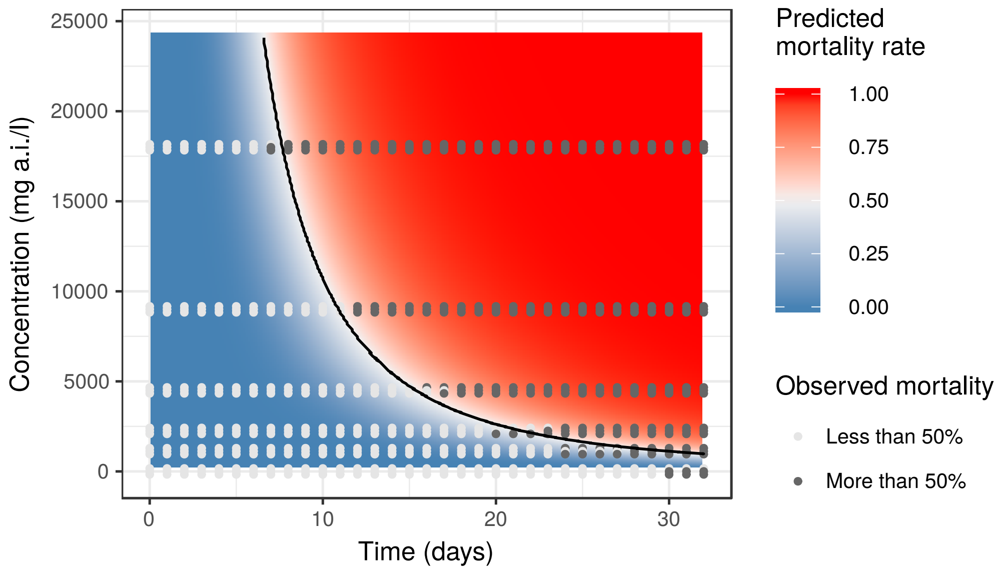

Figure 35:

Conclusion
The results obtained with this method are puzzling. Using the concentration or the dose gives very different results and both results seem to be unlikely.

There is also something strange from the theoretical point of view. If

$$C^{\alpha}t^{\beta} = k$$

then

$$\alpha log(C) + \beta log(t) = log(k)$$

(where k is a given level of mortality) but here we model instead

$$\alpha log(C) + \beta log(t) = k$$

. This would be OK if used a log link but here we use a probit (after the recommendation of Miller et al. 2000)

6 Comparison of the 4 types of models

NB : the aim of this rather long section is to explain why we choose a Weibull 2 model in all the analyses shown above.
In most of the studies the type of model is chosen *a priori* and without justification.

We compare 4 types of dose response curves (logistic, loglogistic, weibull 1 and weibull 2) for each day between D8 and D25 and for each type of effect : LC, LDD, LCD. A similar approach is used to compute the LT (using the data from all days). The aim is to choose the model with the best fit (most of the time) and best statistical properties.

We compare the quality of the fit of each model by extracting the AIC value and computing the difference between the best model (with lowest AIC) and the other AIC values for a given time (for LC, LDD, LCD) or a given concentration (for LT). We have also computed a goodness of fit test (`modelFit` function from `drc` package) for each model (available in the raw outputs). We compare also graphically the differences of LCx, LDDx, LCDx and LTx computed with each model.

Here are the formulas of the 4 models (see Ritz et al. 2010 for more details):

3 parameters Logistic Model (with d = 1) :

$$f(x) = c + \frac{d - c}{(1 + \exp(b(x - e)))}$$

3 parameters Log-Logistic Model (with d = 1)

$$f(x) = c + \frac{d - c}{(1 + \exp(b(\log(x) - \log(e))))}$$

3 parameters Weibull 1 Model (with d = 1)

$$f(x) = c + (d - c)\exp(-\exp(b(\log(x) - \log(e))))$$

3 parameters Weibull 2 Model (with d = 1)

$$f(x) = c + (d - c)(1 - \exp(-\exp(b(\log(x) - \log(e)))))$$

6.1 Main results

Here are the main conclusion for the data analysis below.

The logistic model and Weibull2 model are almost always the best models (the ones with the lowest AIC). The logistic has some times a much lower AIC than the Weibull2 model (particularly after Day 20) however logistic model is often unable to estimate the standard errors of the parameters while the Weibull2 models has almost always standard errors. In addition LCx, LDDx, LCDx and LTx estimates from both models are often very close and the standard errors are quite similar.

So we decided to the the Weibull2 model as a good compromise for the this study.

6.2 Lethal concentrations (LCx) at each time

First lines of the AIC and goodness of fit results (note that ED50 stands for LC50 here):

Time	Model	df	Chisq	p	AIC	DeltaAIC	ED50	LowerCI	UpperCI
8	Logistic	12	11.92	0.452	35.72	0	14818	NA	NA
8	Weibull2	9	11.46	0.245	36.5	0.78	14729	12056	17402
8	LogLogistic	12	12.38	0.416	37.6	1.882	14452	12115	16789
8	Weibull1	9	13.68	0.134	40.9	5.187	14787	11397	18177
9	Logistic	9	7.485	0.587	29.78	0	11556	NA	NA
9	Weibull2	9	8.076	0.527	30.27	0.496	11572	9935	13209
9	LogLogistic	12	10.72	0.553	33.47	3.69	10886	9207	12566
9	Weibull1	9	14.28	0.113	40.15	10.37	10114	8040	12187
10	Logistic	12	6.099	0.911	29.43	0	10030	NA	NA
10	Weibull2	15	7.135	0.954	29.54	0.106	9949	8423	11476
10	LogLogistic	15	10.3	0.8	32.87	3.444	9527	7872	11183
10	Weibull1	15	13.92	0.531	37.88	8.448	8823	7195	10452

First lines of the LCx results

Time	PctMort	Model	Estimate	Std..Error	Lower	Upper
8	10	Logistic	6546	NA	NA	NA
8	20	Logistic	9599	NA	NA	NA
8	30	Logistic	11628	NA	NA	NA
8	40	Logistic	13292	318	12669	13915
8	50	Logistic	14818	NA	NA	NA
8	60	Logistic	16345	NA	NA	NA
8	70	Logistic	18008	NA	NA	NA
8	80	Logistic	20038	NA	NA	NA
8	90	Logistic	23091	NA	NA	NA
8	10	LogLogistic	6569	535.5	5519	7618
8	20	LogLogistic	8788	720.3	7376	10199
8	30	LogLogistic	10663	876.6	8945	12381
8	40	LogLogistic	12495	1029	10478	14512
8	50	LogLogistic	14452	1192	12115	16789
8	60	LogLogistic	16716	1381	14009	19423

Comparison of the delta AIC values (on a log10(x+1) scale) for the 4 types of models at each day after treatment (between D8 and D25). The horizontal dotted red line is the classical threshold of difference of AIC = 2. When a dot is plotted, it means that the LC50 was estimated at that day but that no standard error (and hence no confidence interval) could be estimated by the model).

Interpretation : The logistic model is almost always the best model or very close to it however from day 8 to day 15 the standard error of the LC50 could not be estimated by the model. The Weibull2 model has generally an AIC very close to the logistic model excepted at days >15 where it regularly peaks far away from the best model.

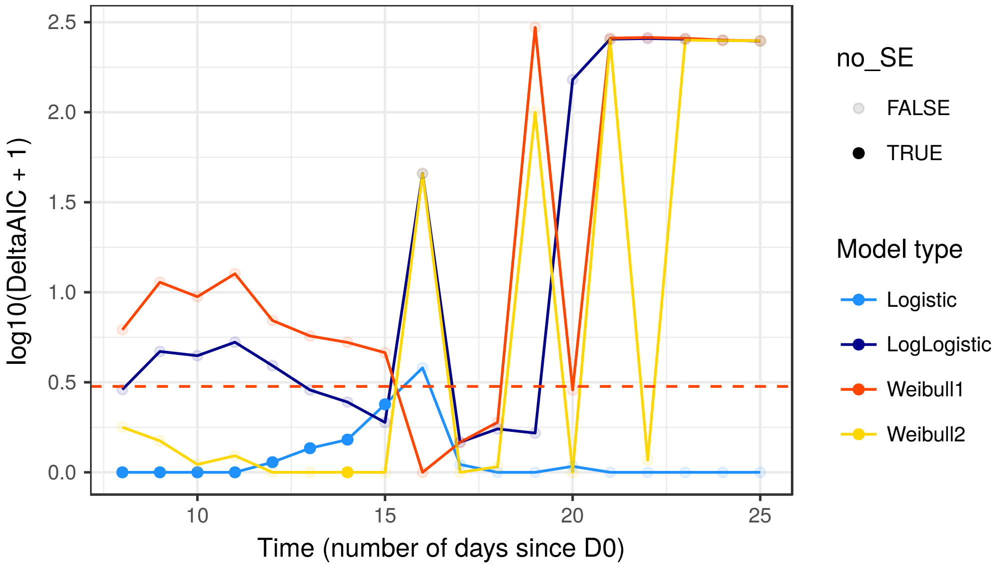

Figure 36:

If you compare the LCx estimates for each rate (LC10, LC20,. . .) for the 4 types of models you can see that the estimates of the Weibull2 model and Logistic models are very close to each other. For the LC50 all four models provide similar estimates.
The Weibull1 and LogLogistic models can deviate quite strongly from the others particularly for the estimates of LC10, LC20, LC80 and LC90.

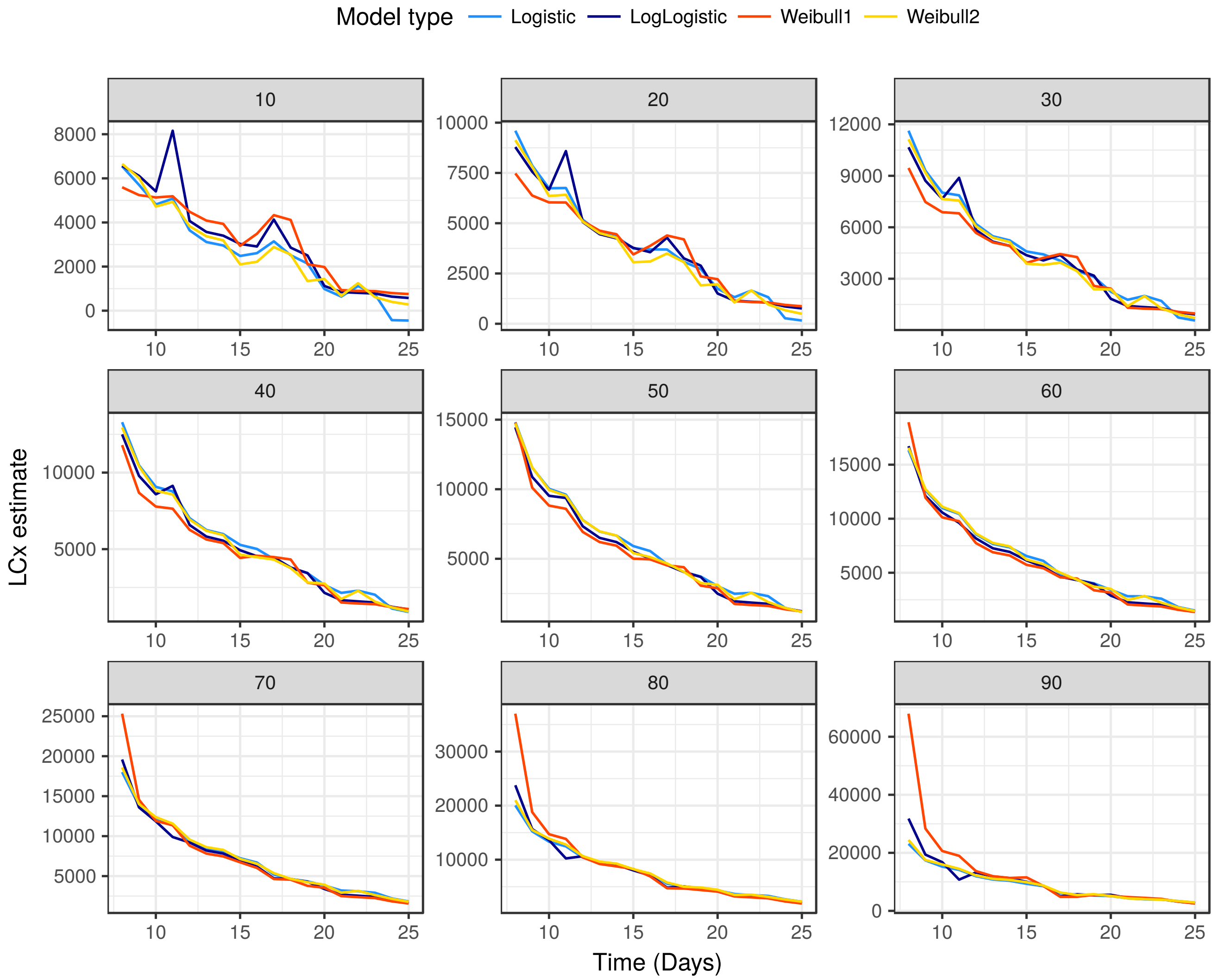

Figure 37:

Here we compare the standard errors of 3 of the models types for the LC50 estimates. You can see that even after day 15 where the Weibull2 models had some times AIC clearly higher that the Logistic model, both the estimates and their confidence intervals are very similar (and confidence intervals are not available for logistic models for days 8-15).

The Weibull 2 models seem to be a good compromise that works well in almost situations. The confidence interval is not available on day 14 and on day 13 it abnormally small. However the estimates at these dates are quite similar to the estimates of other models and within the range of the confidence interval of the next best model (Weibull1)

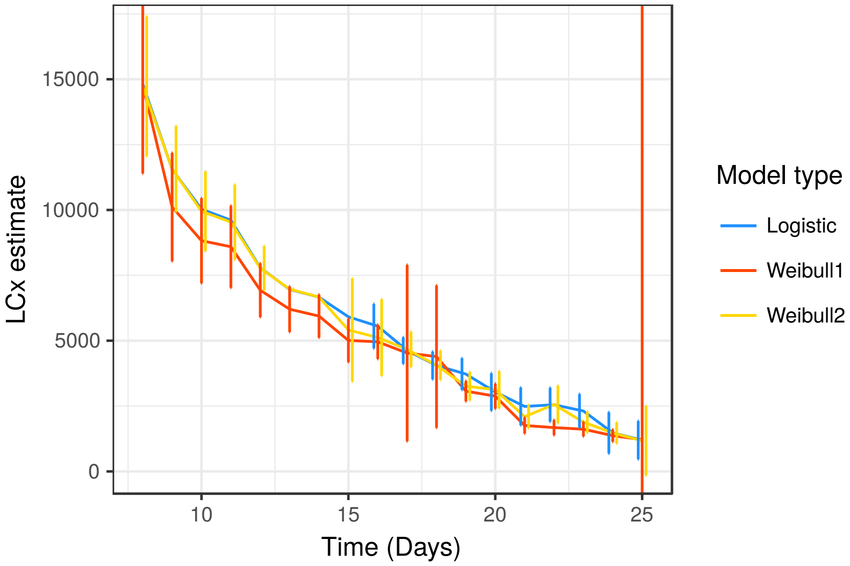

Figure 38:

Conclusion : the Weibull2 model seem to be a good compromise that can be used to estimates LC50 and other LCx at all days between D8 and D25.

6.3 Lethal dietary dose (LDDx) at each time

Similar approach but instead of the nominal concentration of each treatment, we use the effective average dose of a.i. consumed by the bees since D0.

First lines of the AIC and goodness of fit results (note that ED50 stands for LDD50 here):

Time	Model	df	Chisq	p	AIC	DeltaAIC	ED50	LowerCI	UpperCI
8	Logistic	9	6.962	0.641	30.7	0	0.316	NA	NA
8	Weibull2	9	7.479	0.587	31.29	0.591	0.318	0.273	0.362
8	LogLogistic	9	8.762	0.46	32.37	1.666	0.309	0.255	0.362
8	Weibull1	9	11.22	0.261	36.31	5.606	0.309	0.23	0.388
9	Weibull2	9	2.654	0.976	23.85	0	0.265	0.231	0.299
9	Logistic	8	2.737	0.95	24.18	0.332	0.261	0.204	0.318
9	LogLogistic	9	5.541	0.785	27.09	3.245	0.249	0.214	0.284
9	Weibull1	6	10.15	0.118	33.41	9.56	0.235	0.197	0.274
10	Logistic	11	7.528	0.755	29.77	0	0.237	NA	NA
10	Weibull2	12	8.135	0.775	30.15	0.377	0.245	0.199	0.29
10	LogLogistic	15	10.4	0.794	31.2	1.424	0.226	0.192	0.261
10	Weibull1	15	10.97	0.755	33.51	3.742	0.214	0.182	0.246

First lines of the LDDx results

Time	PctMort	Model	Estimate	Std..Error	Lower	Upper
8	10	Logistic	0.1494	0.01018	0.1294	0.1693
8	20	Logistic	0.2108	NA	NA	NA
8	30	Logistic	0.2517	NA	NA	NA
8	40	Logistic	0.2852	NA	NA	NA
8	50	Logistic	0.3159	NA	NA	NA
8	60	Logistic	0.3466	NA	NA	NA
8	70	Logistic	0.3801	NA	NA	NA
8	80	Logistic	0.421	NA	NA	NA
8	90	Logistic	0.4824	NA	NA	NA
8	10	LogLogistic	0.1637	0.02181	0.1209	0.2064
8	20	LogLogistic	0.2068	0.02084	0.166	0.2477
8	30	LogLogistic	0.2416	0.0211	0.2003	0.283
8	40	LogLogistic	0.2745	0.02306	0.2293	0.3197
8	50	LogLogistic	0.3085	0.02713	0.2554	0.3617
8	60	LogLogistic	0.3468	0.03388	0.2804	0.4133

Comparison of the delta AIC values (on a log10(x+1) scale) for the 4 types of models at each day after treatment (between D8 and D25). The horizontal dotted red line is the classical threshold of difference of AIC = 2. When a (colored) dot is plotted, it means that the LDD50 was estimated at that day but that no standard error (and hence no confidence interval) could be estimated.

Interpretation : The logistic model is almost always the best model or very close to it however for several days, the standard error of the LCD50 could not be estimated by the model. The Weibull2 model has generally an AIC very close to the logistic model excepted at days >20 where it far away from the best model.

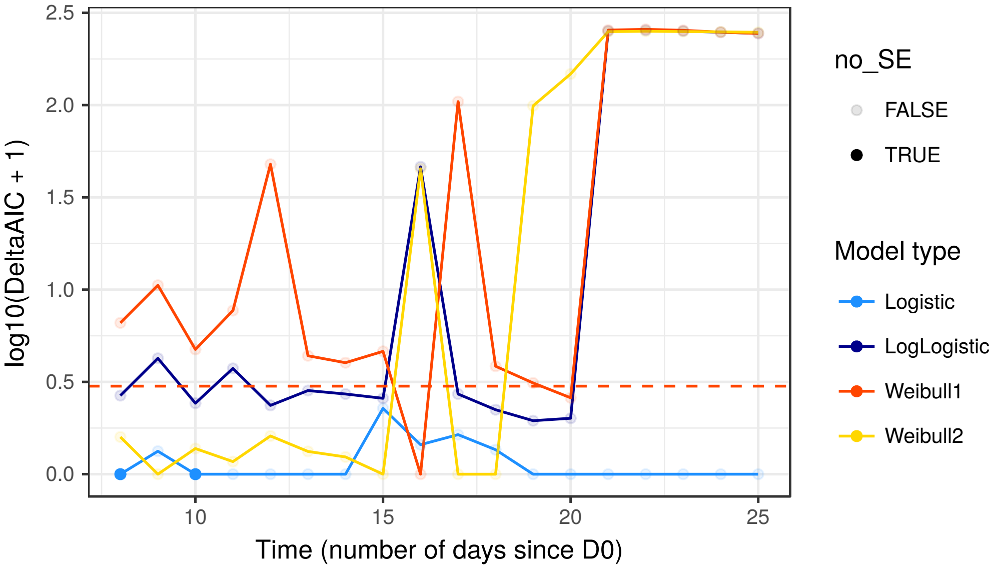

Figure 39:

The different models show very similar patterns excepted the model Weibull1 that deviates some times from the others. For the LDD10 estimates there is also more variability between the models with model Weibull1 being the closest to

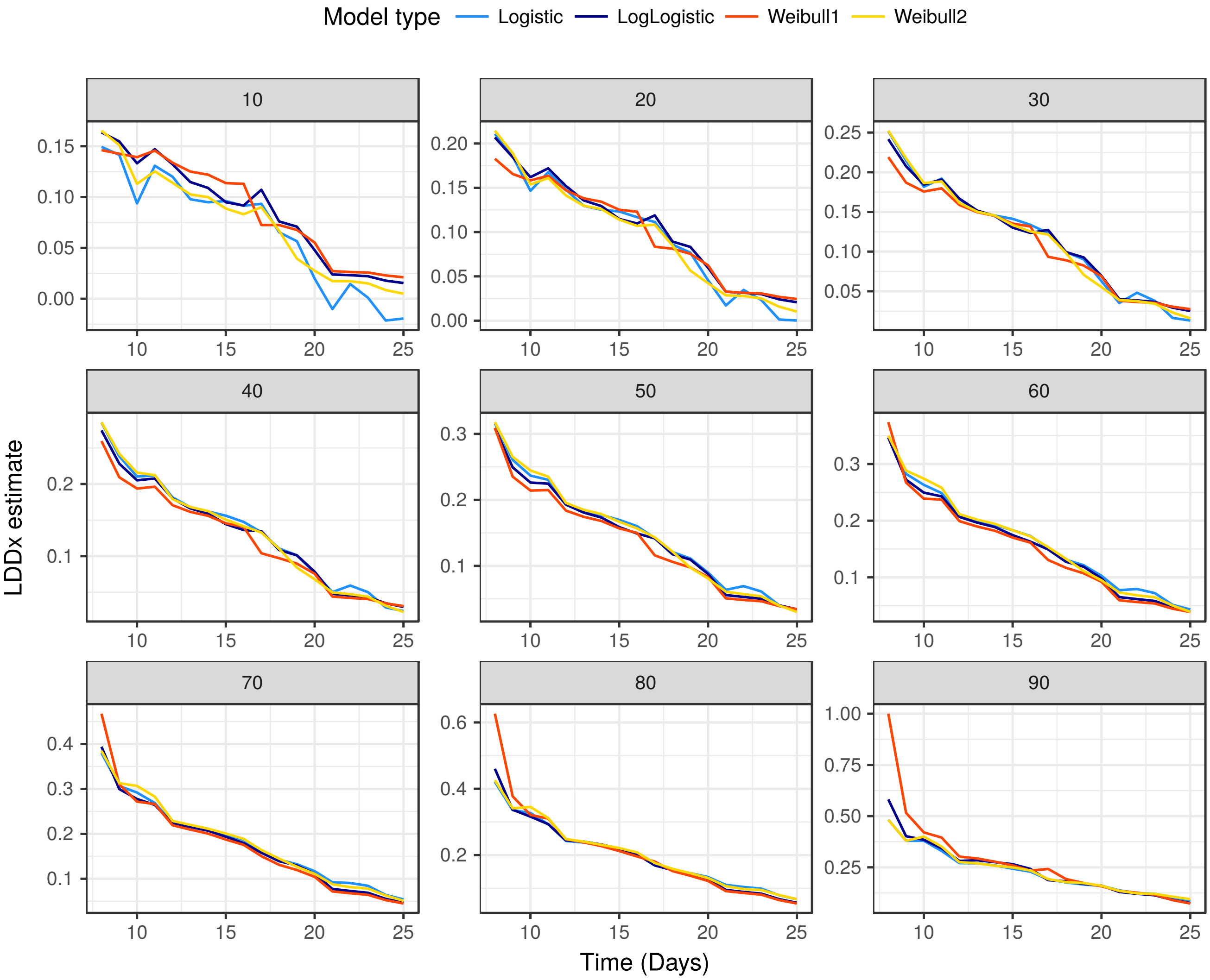

Figure 40:

Comparison of the confidence intervals

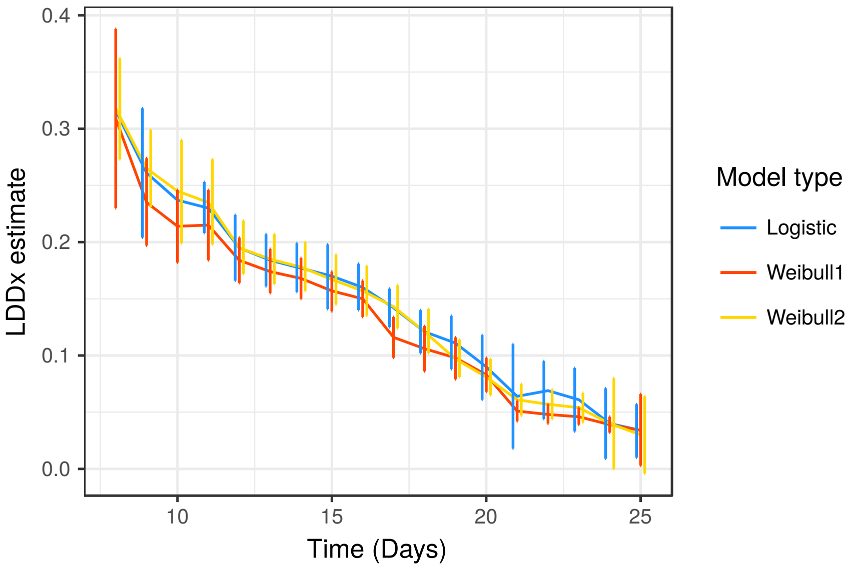

Figure 41:

Conclusion : the Weibull2 model seems to be a good compromise that can be used to estimate LDD50 and other LDDx at all days between D8 and D25. The logistic model has often a better AIC than the Weibull2 model but at several time points, the logistic model is unable to estimate the standard error of the LDD50. In addition the estimates of both models are very close to each other so using one or the other model should not change the final results.

6.4 Lethal cumulative dose (LCDx) at each time

Similar approach but instead of the nominal concentration we use the effective total dose of a.i. consumed by the bees.

First lines of the AIC and goodness of fit results (note that ED50 stands for LCD50 here):

Time	Model	df	Chisq	p	AIC	DeltaAIC	ED50	LowerCI	UpperCI
8	Logistic	9	6.962	0.641	30.7	0	2.527	2.052	3.002
8	Weibull2	9	7.479	0.587	31.29	0.591	2.542	2.187	2.896
8	LogLogistic	9	8.762	0.46	32.37	1.666	2.468	2.043	2.894
8	Weibull1	9	11.22	0.261	36.31	5.606	2.472	1.84	3.104
9	Weibull2	9	2.654	0.976	23.85	0	2.388	2.106	2.67
9	Logistic	8	2.737	0.95	24.18	0.332	2.345	1.981	2.709
9	LogLogistic	9	5.541	0.785	27.09	3.245	2.244	1.929	2.56
9	Weibull1	6	10.15	0.118	33.41	9.56	2.117	1.77	2.464
10	Logistic	11	9.521	0.574	31.07	0	2.321	1.978	2.663
10	Weibull2	12	10.53	0.57	31.9	0.83	2.382	2.015	2.75
10	LogLogistic	15	11.24	0.735	32.24	1.169	2.232	1.911	2.553
10	Weibull1	15	11.86	0.689	34.62	3.547	2.116	1.812	2.42

First lines of the LCDx results

Time	PctMort	Model	Estimate	Std..Error	Lower	Upper
8	10	Logistic	1.195	0.04403	1.109	1.281
8	20	Logistic	1.687	0.1203	1.451	1.923
8	30	Logistic	2.014	0.1681	1.684	2.343
8	40	Logistic	2.281	0.2069	1.876	2.687
8	50	Logistic	2.527	0.2423	2.052	3.002
8	60	Logistic	2.773	0.2777	2.229	3.317
8	70	Logistic	3.041	0.3162	2.421	3.661
8	80	Logistic	3.368	0.363	2.656	4.079
8	90	Logistic	3.859	0.4335	3.01	4.709
8	10	LogLogistic	1.309	0.1745	0.9673	1.651
8	20	LogLogistic	1.654	0.1667	1.328	1.981
8	30	LogLogistic	1.933	0.1688	1.602	2.264
8	40	LogLogistic	2.196	0.1845	1.834	2.557
8	50	LogLogistic	2.468	0.217	2.043	2.894
8	60	LogLogistic	2.775	0.2711	2.243	3.306

Comparison of the delta AIC values (on a log10(x+1) scale) for the 4 types of models at each day after treatment (between D8 and D25). The horizontal dotted red line is the classical threshold of difference of AIC = 2. When a dot is plotted, it means that the LCD50 was estimated at that day but that no standard error (and hence no confidence interval) could be estimated by the model).

Interpretation : The logistic model is almost always the best model or very close to it however for several days, the standard error of the LCD50 could not be estimated by the model. The Weibull2 model has generally an AIC very close to the logistic model excepted at days >20 where it far away from the best model.

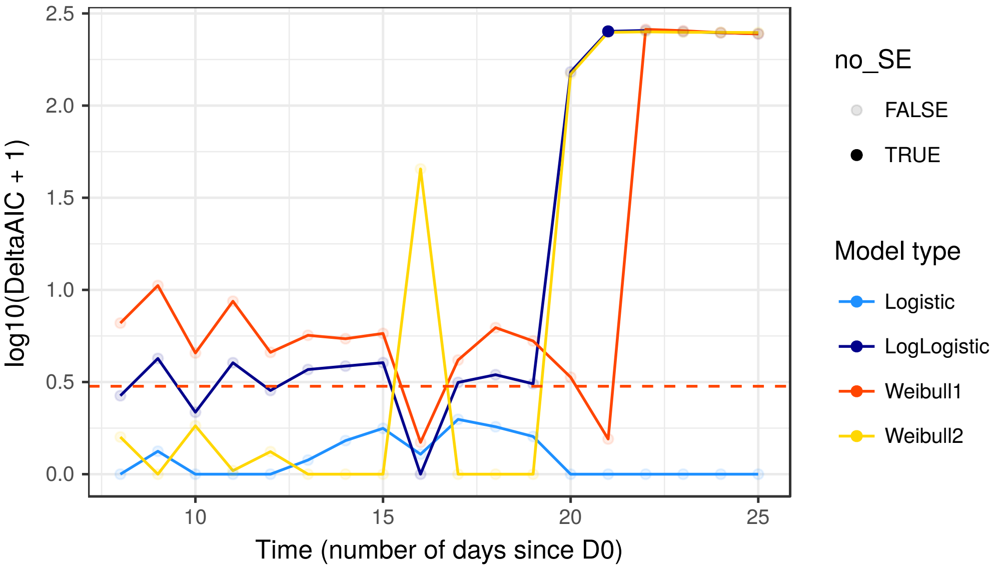

Figure 42:

The different models show similar patterns. There is a very interseting plateau of the LCDx followed by a drop. Without cumulative toxicity, you would expect to have a LCDx independent of time (flat line).

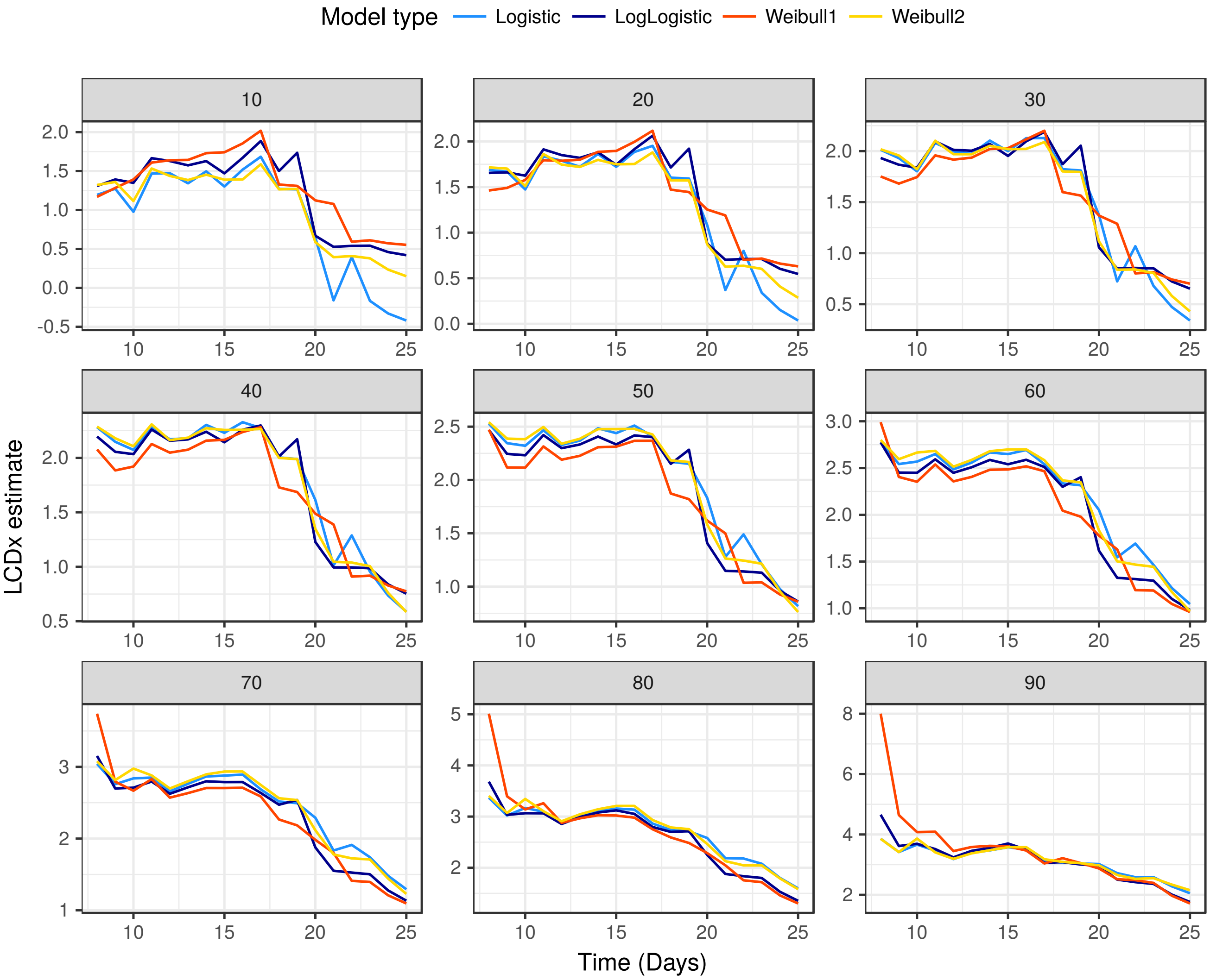

Figure 43:

Comparison of the confidence intervals

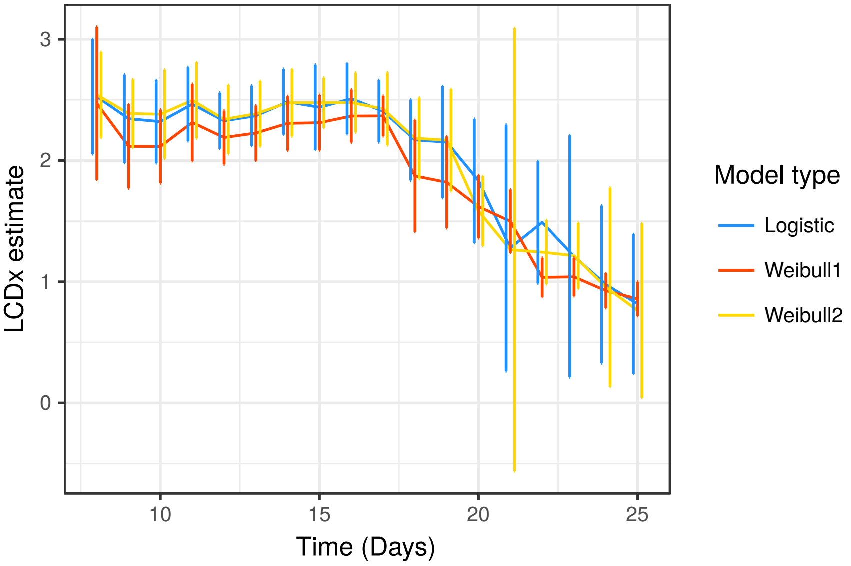

Figure 44:

Conclusion : the Weibull2 model seems to be a good compromise that can be used to estimate LCD50 and other LCDx at all days between D8 and D25.

6.5 Lethal time (LTx) at each concentration

Same approach as for the Lethal Concentration but we fit 4 types of models of the mortality vs time for each concentration First lines of the AIC and goodness of fit results (note that ED50 stands for LT50 here):

Conc	Model	df	Chisq	p	AIC	DeltaAIC	ED50	LowerCI	UpperCI
0	Weibull2	51	54.04	0.359	159.7	0	32	29.84	34.16
0	LogLogistic	51	55.35	0.314	160.3	0.521	32.39	30.06	34.72
0	Logistic	54	56.27	0.39	160.5	0.794	31.53	NA	NA
0	Weibull1	51	55.47	0.31	161.4	1.685	34.51	32.93	36.08
1125	Weibull2	51	86.44	0.001	172	0	24.93	24.38	25.47
1125	Logistic	51	91.61	0	178.8	6.84	24.68	24.33	25.03
1125	LogLogistic	51	96.16	0	183.5	11.53	24.61	23.98	25.24
1125	Weibull1	54	104.1	0	195.8	23.84	24.02	23.34	24.7
2250	Weibull2	96	78.87	0.898	141.4	0	21.99	21.48	22.5
2250	Logistic	96	82.55	0.834	148.3	6.856	21.74	21.24	22.24
2250	LogLogistic	96	85.06	0.78	153.6	12.15	21.64	21.15	22.13
2250	Weibull1	96	93.28	0.559	166.8	25.41	21.23	20.75	21.72

First lines of the LTx results

Conc	PctMort	Model	Estimate	Std..Error	Lower	Upper
0	10	Logistic	19.67	0.5174	18.66	20.68
0	20	Logistic	24.05	0.3191	23.42	24.67
0	30	Logistic	26.96	0.1871	26.59	27.32
0	40	Logistic	29.34	0.07787	29.19	29.49
0	50	Logistic	31.53	NA	NA	NA
0	60	Logistic	33.72	0.1163	33.49	33.95
0	70	Logistic	36.1	0.225	35.66	36.54
0	80	Logistic	39.01	0.3569	38.31	39.71
0	90	Logistic	43.39	0.5551	42.3	44.48
0	10	LogLogistic	19.88	0.4511	18.99	20.76
0	20	LogLogistic	23.8	0.6636	22.5	25.1
0	30	LogLogistic	26.83	0.8399	25.18	28.48
0	40	LogLogistic	29.6	1.009	27.62	31.58
0	50	LogLogistic	32.39	1.188	30.06	34.72
0	60	LogLogistic	35.44	1.39	32.72	38.17

Comparison of the delta AIC values for the 4 types of models at each concentration. The horizontal dotted red line is the classical threshold of difference of AIC = 2. When a dot is plotted, it means that the LT50 was estimated at that day but that no standard error (and hence no confidence interval) could be estimated by the model).

Interpretation : The weibull2 model is almost always the best model.

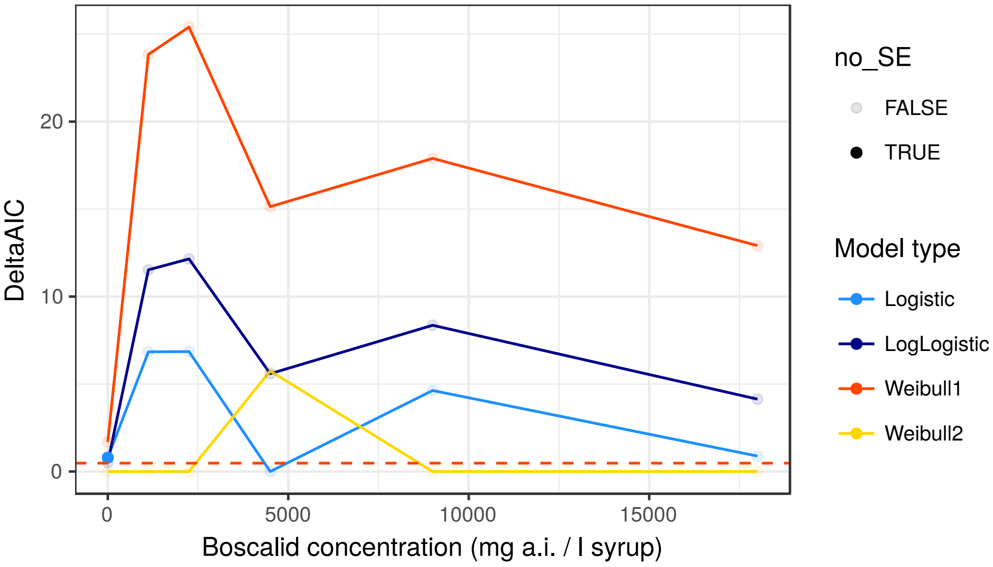

Figure 45:

The LT50 estimates are very similar for all 4 models. There are some differences for other mortality thresholds particularly for LT10.

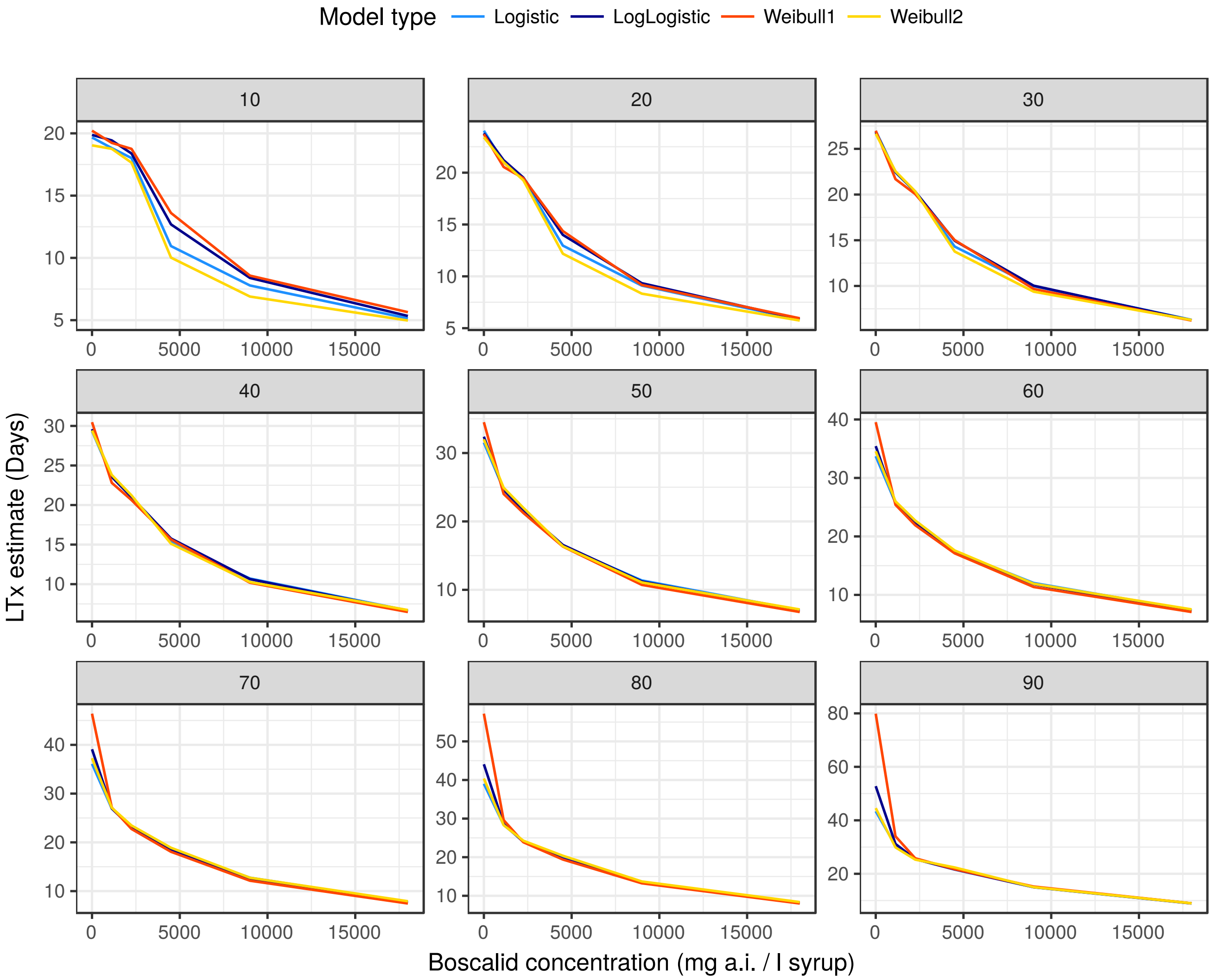

Figure 46:

Here we compare the standard errors of 3 of the models types for the LT50 estimates.

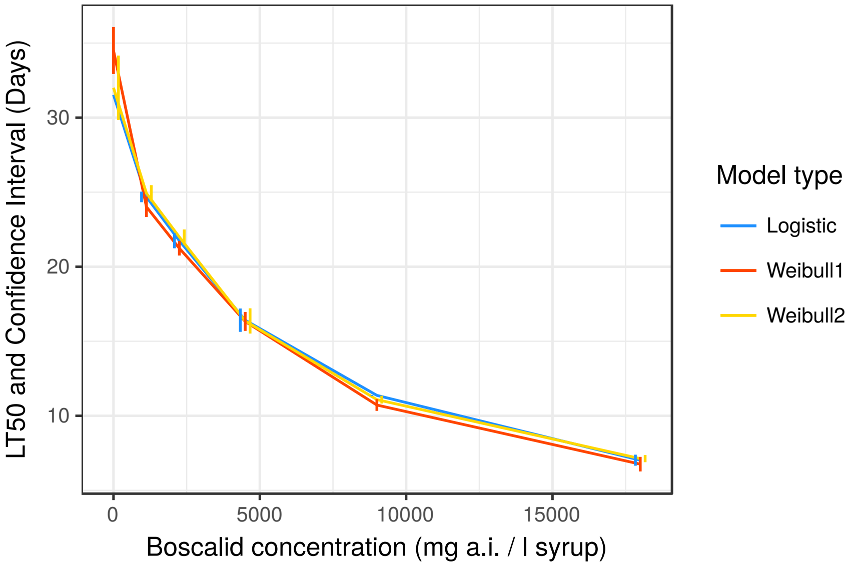

Figure 47:

The same with LT10

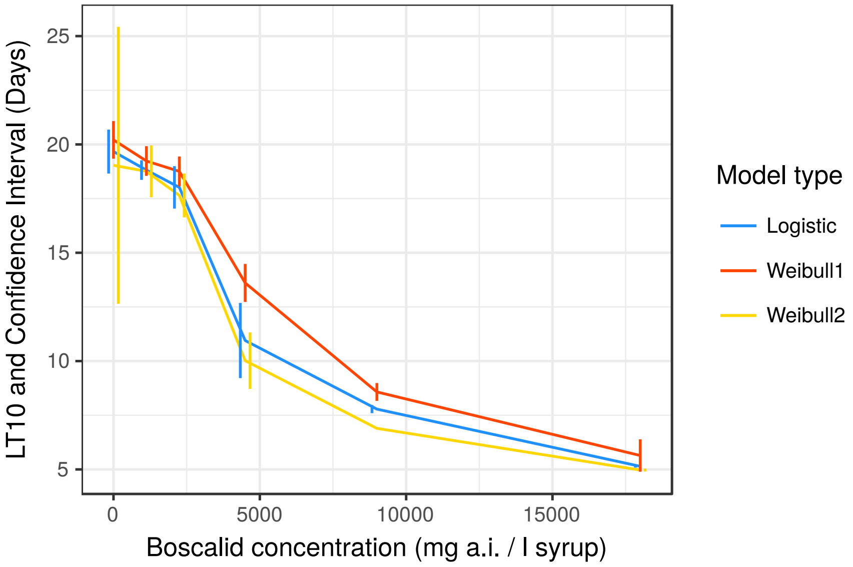

Figure 48:

Conclusion : the Weibull2 model seems to be a good compromise : it is always the best model with only one exception and the estimates are close to the other models in most circumstances with narrow Confidence Intervals.

7 Comparison of models with 2 or 3 parameters using corrected mortality or not

In the previous analyses we used 3 parameters models and uncorrected mortalities. The results of such models are identical or very similar to estimates of more classical 2 parameters models based on corrected mortalities.

We compare here the results based on these two approaches.

We have used the corrected mortalities (Abbott's formula on the % of dead) and performed 2 parameters models. This approach is not ideal for at least two reasons :

1. the corrected mortality is no more a ratio of to integers (ie because of the unbalance in the design)
2. the total number of individuals used in the model (as weights) remains constant while it should decrease when the correction is higher. However this should have mainly an impact on the standard errors and not on the estimate itself which is our main interest here.

It could be possible to correct directly the number of dead bees and the total and then compute the corrected ratio. This means however that we have to group the data from the 3 replicates, slightly change de way the consumption is calculated and the correction will be unfair for the doses with a different initial number of bees.

The comparison of the 2 approaches shows that the estimates are very close to each other particularly for the Weibull2 model used in the previous analyses.
NB1 : the black line shows a line with slope = 1 and intercept =0 (ie the line showing the perfect match).
NB2 : the few points that deviates in the LogLogistic models and Weibull1 models are due to 2 parameters models with a clear lack of fit and very large standard errors.

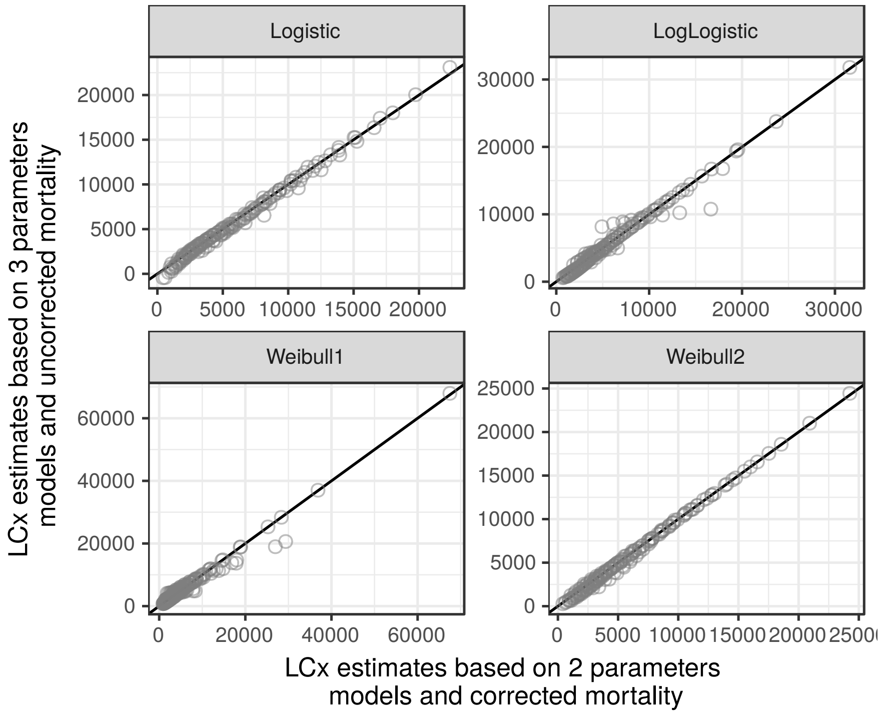

Figure 49:

8 References

Bretz, F., Hothorn, T., Westfall, P., Westfall, P.H., 2010. Multiple Comparisons Using R. CRC Press.

European Food Safety Authority, 2013. Guidance on the risk assessment of plant protection products on bees (Apis mellifera, Bombus spp. and solitary bees). EFSA Journal 2013;11(7):3295, 266 pp. doi: 10.2903/j.efsa.2013.3295.

Miller, F.J., Schlosser, P.M., Janszen, D.B., 2000. Haber's rule: a special case in a family of curves relating concentration and duration of exposure to a fixed level of response for a given endpoint. Toxicology 149, 21–34.

Ritz, C., 2010. Toward a unified approach to dose–response modeling in ecotoxicology. Environmental Toxicology and Chemistry 29, 220–229.

Automatic citation of R and all packages used :

R

R Core Team (2017). *R: A Language and Environment for Statistical Computing.* R Foundation for Statistical Computing, Vienna, Austria. <URL: https://www.R-project.org/>.

car

Fox J and Weisberg S (2011). *An R Companion to Applied Regression*, Second edition. Sage, Thousand Oaks CA. <URL: http://socserv.socsci.mcmaster.ca/jfox/Books/Companion>.

drc

Ritz C, Baty F, Streibig JC and Gerhard D (2015). "Dose-Response Analysis Using R." *PLOS ONE*, *10*(e0146021). <URL: http://journals.plos.org/plosone/article?id=10.1371/journal.pone.0146021>.

ggplot2

Wickham H (2016). *ggplot2: Elegant Graphics for Data Analysis.* Springer-Verlag New York. ISBN 978-3-319-24277-4, <URL: http://ggplot2.org>.

knitr

Xie Y (2018). *knitr: A General-Purpose Package for Dynamic Report Generation in R.* R package version 1.20, <URL: https://yihui.name/knitr/>.

Xie Y (2015). *Dynamic Documents with R and knitr*, 2nd edition. Chapman and Hall/CRC, Boca Raton, Florida. ISBN 978-1498716963, <URL: https://yihui.name/knitr/>.

Xie Y (2014). "knitr: A Comprehensive Tool for Reproducible Research in R." In Stodden V, Leisch F and Peng RD (eds.), *Implementing Reproducible Computational Research.* Chapman and Hall/CRC. ISBN 978-1466561595, <URL: http://www.crcpress.com/product/isbn/9781466561595>.

lme4

Bates D, Mächler M, Bolker B and Walker S (2015). "Fitting Linear Mixed-Effects Models Using lme4." *Journal of Statistical Software*, *67*(1), pp. 1-48. doi: 10.18637/jss.v067.i01 (URL: http://doi.org/10.18637/jss.v067.i01).

MASS

Venables WN and Ripley BD (2002). *Modern Applied Statistics with S*, Fourth edition. Springer, New York. ISBN 0-387-95457-0, <URL: http://www.stats.ox.ac.uk/pub/MASS4>.

Matrix

Bates D and Maechler M (2017). *Matrix: Sparse and Dense Matrix Classes and Methods.* R package version 1.2-11, <URL: https://CRAN.R-project.org/package=Matrix>.

multcomp
Hothorn T, Bretz F and Westfall P (2008). “Simultaneous Inference in General Parametric Models.” *Biometrical Journal*, *50*(3), pp. 346-363.

mvtnorm
Genz A, Bretz F, Miwa T, Mi X, Leisch F, Scheipl F and Hothorn T (2019). *mvtnorm: Multivariate Normal and t Distributions.* R package version 1.0-7, <URL: https://CRAN.R-project.org/package=mvtnorm>.

Genz A and Bretz F (2009). *Computation of Multivariate Normal and t Probabilities*, series Lecture Notes in Statistics. Springer-Verlag, Heidelberg. ISBN 978-3-642-01688-2.

pander
Daróczi G and Tsegelskyi R (2017). *pander: An R ‘Pandoc’ Writer.* R package version 0.6.1, <URL: https://CRAN.R-project.org/package=pander>.

reshape
Wickham H (2007). “Reshaping data with the reshape package.” *Journal of Statistical Software*, *21*(12). <URL: http://www.jstatsoft.org/v21/i12/paper>.

survival
Therneau T (2015). *A Package for Survival Analysis in S.* version 2.38, <URL: https://CRAN.R-project.org/package=survival>.

Terry M. Therneau and Patricia M. Grambsch (2000). *Modeling Survival Data: Extending the Cox Model.* Springer, New York. ISBN 0-387-98784-3.

TH.data
Hothorn T (2017). *TH.data: TH’s Data Archive.* R package version 1.0-8, <URL: https://CRAN.R-project.org/package=TH.data>.

9 Session Information

```
## R version 3.4.3 (2017-11-30)
## Platform: x86_64-pc-linux-gnu (64-bit)
## Running under: Ubuntu 16.04.4 LTS
##
## Matrix products: default
## BLAS: /usr/lib/libblas/libblas.so.3.6.0
## LAPACK: /usr/lib/lapack/liblapack.so.3.6.0
##
## locale:
##  [1] LC_CTYPE=en_GB.UTF-8       LC_NUMERIC=C               LC_TIME=en_GB.UTF-8
##  [4] LC_COLLATE=en_GB.UTF-8     LC_MONETARY=en_GB.UTF-8    LC_MESSAGES=fr_BE.UTF-8
##  [7] LC_PAPER=fr_BE.UTF-8       LC_NAME=C                  LC_ADDRESS=C
## [10] LC_TELEPHONE=C             LC_MEASUREMENT=fr_BE.UTF-8 LC_IDENTIFICATION=C
##
## attached base packages:
## [1] stats     graphics  grDevices utils     datasets  methods   base
##
## other attached packages:
##  [1] multcomp_1.4-8     TH.data_1.0-8      survival_2.41-3    mvtnorm_1.0-7
##  [5] car_2.1-6          lme4_1.1-15        Matrix_1.2-11      reshape_0.8.7
##  [9] drc_3.0-1          MASS_7.3-49        ggplot2_2.2.1.9000 pander_0.6.1
## [13] knitr_1.20
##
## loaded via a namespace (and not attached):
##  [1] Rcpp_0.12.15        lattice_0.20-35     zoo_1.8-1           gtools_3.5.0
##  [5] rprojroot_1.3-2     digest_0.6.15       plyr_1.8.4          backports_1.1.2
##  [9] acepack_1.4.1       MatrixModels_0.4-1  evaluate_0.10.1     pillar_1.1.0
## [13] rlang_0.2.0.9000    lazyeval_0.2.1.9000 minqa_1.2.4         rstudioapi_0.7
## [17] data.table_1.10.4-3 SparseM_1.77        nloptr_1.0.4        rpart_4.1-13
## [21] checkmate_1.8.5     rmarkdown_1.8       labeling_0.3        splines_3.4.3
## [25] stringr_1.3.0       foreign_0.8-69      htmlwidgets_1.0     munsell_0.4.3
## [29] compiler_3.4.3      xfun_0.1            base64enc_0.1-3     mgcv_1.8-23
## [33] htmltools_0.3.6     nnet_7.3-12         tibble_1.4.2        gridExtra_2.3
## [37] htmlTable_1.11.2    bookdown_0.7        Hmisc_4.1-1         codetools_0.2-15
## [41] withr_2.1.1.9000    grid_3.4.3          nlme_3.1-131        gtable_0.2.0
## [45] magrittr_1.5        scales_0.5.0.9000   stringi_1.1.6       latticeExtra_0.6-28
## [49] sandwich_2.4-0      Formula_1.2-2       RColorBrewer_1.1-2  tools_3.4.3
## [53] plotrix_3.7         parallel_3.4.3      pbkrtest_0.4-7      yaml_2.1.16
## [57] colorspace_1.3-2    cluster_2.0.6       quantreg_5.35
```
